# Design, Synthesis of Novel Tetrandrine-14-l-Amino Acid and Tetrandrine-14-l-Amino Acid-Urea Derivatives as Potential Anti-Cancer Agents

**DOI:** 10.3390/molecules25071738

**Published:** 2020-04-09

**Authors:** Sheng-Cao Hu, Jin Yang, Chao Chen, Jun-Rong Song, Wei-Dong Pan

**Affiliations:** 1 College of Pharmacy, Zunyi Medical University, Zunyi 563000, China; hushengcao0221@163.com (S.-C.H.); jinyangtrcwsys@sina.com (J.Y.); 2 State Key Laboratory of Functions and Applications of Medicinal Plants, Guizhou Medical University, Guiyang 550014, China; cc283818640@163.com; 3 The Key Laboratory of Chemistry for Natural Products of Guizhou Province and Chinese Academy of Sciences, Guiyang 550014, China

**Keywords:** tetrandrine derivatives, l-amino acid, urea, anti-cancer activity

## Abstract

Tetrandrine, a dibenzyltetrahydroisoquinoline alkaloid isolated from the root of the traditional Chinese medicinal plant *Stephania tetrandra* S. Moore, a member of the Menispermaceae, showed anti-cancer activity by inhibiting cell proliferation, preventing cell cycle progress and induction of cell death and autophagy. In this study, twelve tetrandrine-l-amino acid derivatives and twelve tetrandrine-14-l-amino acid-urea derivatives were designed and synthesized, using C14-aminotetrandrine as raw material. Then the preliminary in vitro anti-cancer activities of these derivatives against human breast cancer cell line MDA-MB-231, human leukemia cell lines HEL and K562 were evaluated. The in vitro cytotoxicity results showed that these derivatives exhibited potent inhibitory effects on cancer cell growth, and the primary structure-activity relationships were evaluated. Notably, compound **3f** exhibited satisfactory anticancer activity against all three cancer cell lines, especially the HEL cell line, with the IC_50_ value of 0.23 µM. Further research showed that **3f** could induce G1/S cycle arrest and apoptosis in a dose- and time- dependent manner on the leukemia cell line HEL. The results suggested that **3f** may be used as a potential anti-cancer agent for human leukemia.

## 1. Introduction

Cancer is one of the most serious disease threats to human health worldwide. Based on the report of the International Agency for Research on Cancer (IARC), it was estimated that there were 18.1 million new cancer cases and 9.6 million cancer deaths in 2018 [1]. Cancer is the first or second leading cause of death for people under 70 years old across 91 countries at the global level [2]. Chemotherapy has one of the most important ways to fight back against cancer since the 1940s when nitrogen mustard and antifolates were introduced to treat non-Hodgkin’s lymphoma and pediatric acute leukemia [3,4,5]. More than 200 chemotherapeutic drugs have been approved by the FDA for treating cancers, and 75% of them are derived from natural products [6]. Over the past decades, natural products isolated from microorganisms and plants such as doxorubicin, mitomycin C, camptothecin, vincristine, taxol and podophyllotoxin as well as their structurally modified derivatives have been used as approved chemotherapeutic drugs [7,8,9,10].

**Figure 1:**
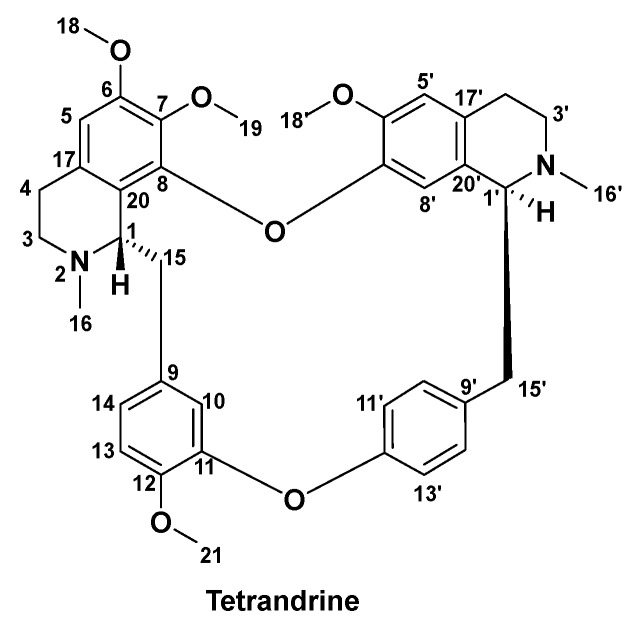
The structure of tetrandrine.

Tetrandrine (Figure 1), a bisbenzylisoquinoline (BBI) alkaloid isolated from the dried roots of the traditional Chinese medicinal herb *Stephania tetrandra* S. Moore [11], has been used as a antiphlogistic, antalgic, calcium channel antagonistic, anti-radical and anticancer agent [12,13,14]. Recent research indicated that the anticancer mechanism of tetrandrine was multifarious. Tetrandrine is used as a potential CDKs inhibitor that directly inhibits CDK4, CDK2-CycE to arrest the cell cycle in the G1/S phase [15,16,17], and then the effects of tetrandrine on controlling the cancer-associated gene (GAGE) expression are able to activate the apoptosis and autophagy pathway in cancer cells [18,19,20]. Aside from the aforesaid anticancer effects, tetrandrine increases the sensibility to other chemotherapeutic drugs and reverses the MDR [21] by regulating ABC transporter activity and reversal of P-g expression [22] and inhibiting the functions of P-gp [23].

As a potential anticancer agent with multiple mechanisms of action, the structural modification of tetrandrine is an attractive subject for many research groups. Since the 21st century, structural modifications have mainly focused on introducing halogens and alkyl groups at the C_5_ and C_14_ positions of tetrandrine [24,25,26], or quaternary ammonium salts at the N_2_ and N_1_ positions [27,28]. Recently, our group prepared a serious of C_14_-amino substituted tetrandrine derivatives which exhibited satisfactory inhibitory effects on human hepatocellular carcinoma (HCC), human leukemia (HEL and K562), human breast carcinoma (MDA-MD-231), human PCa (PC3), and human melanoma (WM9) cell lines [29,30,31]. Even though these derivatives are reported as potential anticancer agents, their poor water solubility and low bioavailability limits their application for developing lead anticancer compounds [32,33].

**Figure 2:**
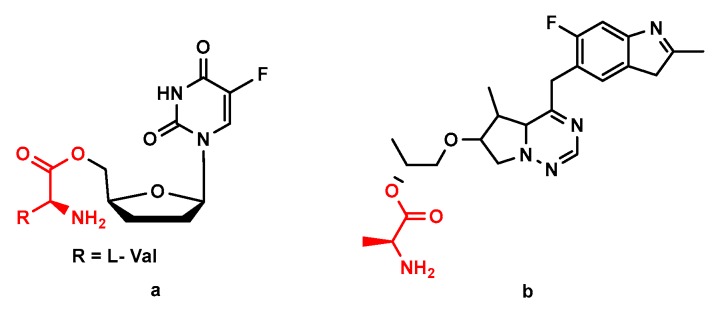
The structures of floxuridine prodrug (**a**) and brivanib (**b**).

Amino acid functional groups often used for development of antiviral, antiparasitic, antibacterial and anticancer drugs [34,35], in order to improve the oral absorption, sensitivity, physiochemical property and pharmacology of drugs [36]. Further studies showed that certain cancer cells were rich in oligopeptide transporters on their cytomembrane [37,38], so the amino acid fragment was promising for the improvement of the selectivity of anticancer drugs [39], such as floxuridine and brivanib (Figure 2) [40,41]. In addition to amino acid fragments, the aryl urea moiety was also proved to be good fragment for anticancer agents [42]. Based on this background, we have now designed and synthesized a series of tetrandrine derivatives with amino acid and urea groups at the C_14_-position and evaluated their in vitro anticancer bioactivity. Primary SAR and mechanistic studies were also performed in this study.

## 2. Results and Discussion

### 2.1. Chemistry

**Scheme 1:**
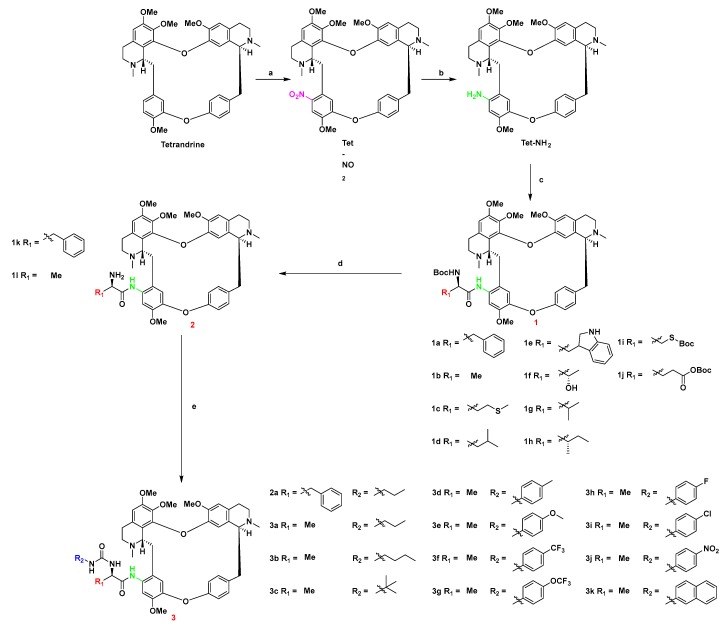
The synthetic routes of tetrandrine derivatives*. Reagents and Conditions*: (**a**) mixed acid (20 eq, HNO_3_: acetic anhydride = 7:10 *v*/*v*), DCM, 0 °C to r.t., 4 h (93%); (**b**) Pd/C (5%), hydrazine hydrate (80 eq), MeOH, 65 °C, 3.5 h (84%); (**c**) Boc-l-amino acid (1.1 eq), EDCI (1.1 eq), HOBT (0.4 eq), DCM, r.t., 1.5–3 h (78-88%); (**d**) TFA (1.0 eq), DCM, 0 °C to r.t., 4 h (97%); (**e**) isocyanate (1.1 eq), triethylamine (0.2 eq), DCM, r.t., 0.5–1.5 h (90–95%).

The synthetic route of tetrandrine derivatives **1a**–**3k** is shown in Scheme 1. The mixture of concentrated nitric acid and acetic anhydride at low temperature was used as nitration reagent to obtain C14-nitro-tetrandrine selectively. The nitrotetrandrine could be restored to amino-substituted tetrandrine by using hydrazine hydrate as reducing agent in a methanol reaction medium containing palladium on carbon [43]. The C_14_-amino-tetrandrine was then reacted with Boc-l-amino acids in the presence of EDCI and HOBT to give tetrandrine-l-amino acid derivatives **1** in good yield. The tert-butyl carbonate groups of **1a** and **1b** were removed in a mixed solvent of CH_2_Cl_2_ and TFA at room temperature to obtain compounds **1k** and **1l**, which were then reacted with isocyanate to give tetrandrine-l-amino acid-urea derivatives **2a**–**3k** in satisfactory yield.

### 2.2. Biological Evaluation

#### 2.2.1. In Vitro Cytotoxicity Assay

Twenty-seven tetrandrine derivatives were tested for their cytotoxicity against a human leukemia cell line (HEL), K562 and a breast cancer cell line (MDA-MB-231). The IC_50_ values of the tetrandrine derivatives, positive control vinblastine, the parent compounds tetrandrine and fangchinoline for 48 h were determined by the MTT assay [44], as presented in Table 1.

Compared with vinblastine, tetrandrine and fangchinoline, most of the tetrandrine derivatives showed better in vitro anti-cancer activities on all the three human cancer cell lines and the IC_50_ values were as follows: 0.230-13.856 µM for HEL, 0.392-15.025 µM for K562, 0.812-9.088 µM for MDA-MB-231, respectively. Among the derivatives, six of them (**1c**, **1i**, **3f**-**3i**) showed better inhibitory effects on HEL cell line with IC_50_ values of 0.631, 0.821, 0.230, 0.261, 0.386 and 0.940 µM, respectively. The compound **3f** showed the strongest cytotoxic activity, so it was chosen for further mechanistic studies.

#### 2.2.2. Structure-Activity Relationship Study

Based on the MTT results, a preliminary Structure-Activity Relationship (SAR study could be performed. The substitution of L-amino acid and L-amino acid-urea, which are supposed to introduce a pivotal pharmacophore at the C_14_-position of tetrandrine, could enhance the anti-cancer activities of the derivatives.

Compared with the cytotoxicity of the tetrandrine-l-amino acid derivatives on all three cell lines, tetrandrine-l-amino acid-urea derivatives showed better anti-cancer activities. For compounds **1a**–**1l**, when the R_1_ substituents are electron-withdrawing side chains (i.e., compounds **1i**, **1j**), these compounds showed worse in vitro anti-cancer activities than those compounds whose R_1_ substituents contained electron-donating side chains (**1a**, **1c**, **1e**). Longer branched aliphatic side chain substituents at R_1_ were able to improve the inhibitory effects of the compounds (**1d**, **1g**, **1h**), as these compounds showed better activities than compound **1b**, whose R_1_ substituent was a methyl. The anti-cancer activities of compounds **1a** and **1k** didn’t display prominent differences on the three cancer cell lines and the same situation happened between compounds **1b** and **1l**, so it followed that the *tert*-butyl carbonate group on the L-amino acid substituent was not essential for anti-cancer activity.

Compounds **2a**–**3k** showed better inhibitory effects on HEL and MDA-MB-231 cell lines than K562 cell line. The change of R_1_ substituent in the tetrandrine-l-amino acid-urea derivatives could influence their inhibitory effects, on account of the different R_1_ substituents, compounds **2a** and **3a** showed prominent differences in anti-cancer activity. Compound **2a**, whose R_1_ substituent was benzyl, showed better activities on HEL and K562 cell line with IC_50_ values of 1.171 µM and 1.616 µM, which were 2-fold and 9-fold higher than compound **3a,** whose R_1_ substituent was a methyl. The probable cause of the different activities between compounds **2a** and **3a** was the electronic effect of the R_1_ substituent. The electron accepting effect of the R_2_ substituent could also affect the anti-cancer activities of tetrandrine-l-amino acid-urea derivatives. When the R_2_ substituent was a phenyl with electron-withdrawing groups (-F, -CF_3_, -OCF_3_, -Cl) in the *para*-position, the products showed increased antiproliferative activities (**3f**–**3i**).

#### 2.2.3. The Effect of Compound **3f** on Cell Proliferation

**Figure 3:**
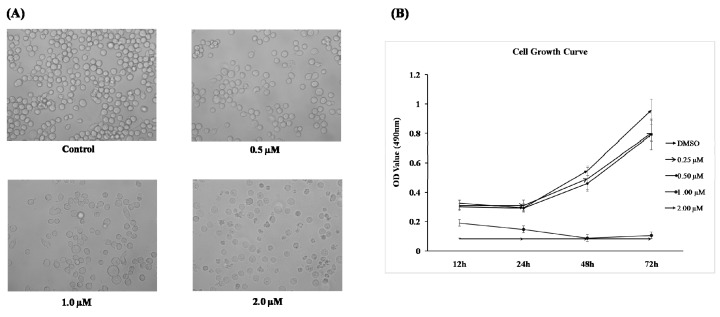
The inhibitory activity on proliferation of human leukemia HEL cell of **3f**. (**A**) Cellular morphological alteration of HEL cell at different concentrations of **3f** after 24 h of drug treatment. (**B**) The inhibition of **3f** on HEL cell growth after 72 h.

Microscopic examination was used to evaluate morphological changes within HEL cells. Cell growth curves were observed by measuring the OD value at 12, 24, 48 and 72 h using the MTT method, where the OD value is proportional to the cell viability. Compared with the control group, the microscopy examination (Figure 3A) showed that the number of HEL cells was significantly reduced and cells had obviously died and dispersed, with the appearance of apoptotic bodies. The cell growth curve (Figure 3B) showed that compound **3f** exerted inhibitory activity on the proliferation of the HEL cell line in a time and dose dependent manner (Figure 3).

#### 2.2.4. Compound **3f** Induced Cell Apoptosis on HEL Cell Line

**Figure 4:**
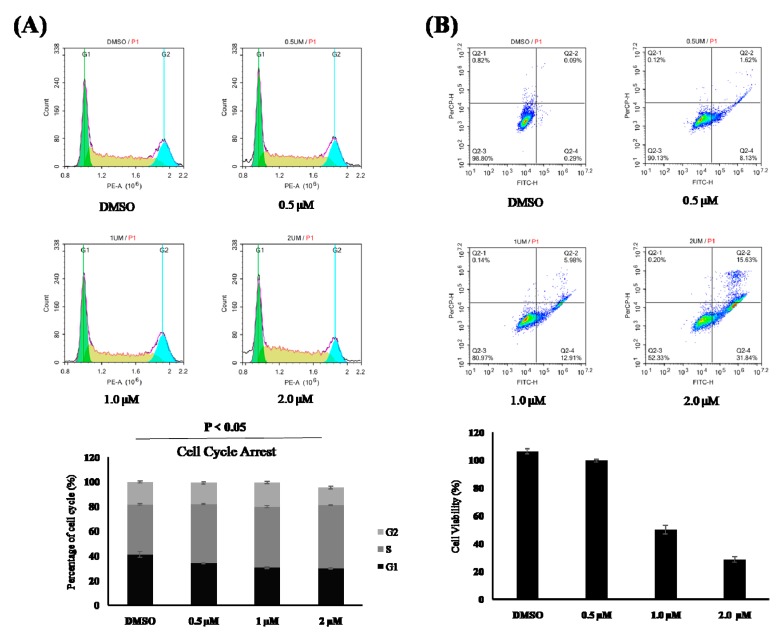
Apoptosis induced by compound **3f** in HEL cell line. (**A**) Compound **3f** had effect in retardation of cell cycle progression in HEL cell line. The cell cycle progression was retarded in the G1/S phase. HEL cell line was treated with compound **3f** for 24 h. (**B**) Compound **3f** induced apoptosis in HEL cell line. The HEL cell line was treated with compound **3f** for 24 h and analyzed by flow cytometry, using Annexin V/PI staining.

Depending on the effects of **3f** on cell cycle progression, it was shown that the treatment of compound **3f** led to the cell cycle arrest of the HEL cell line in the G1/S phase (Figure 4A). Because the **3f** treatment led to cellular morphological transformation and cell death, the effects of compound **3f** on cell apoptosis were tested as well. Flow cytometry analysis showed that **3f** treatment significantly increased the proportion of early apoptotic cells from 0.29% to 8.13%, 12.91% and 31.84%, and the proportion of late apoptotic cells was also increased from 0.09% to 1.62%, 5.98% and 15.63% after **3f** treatment (Figure 4B) in a dose dependent manner. From these results, it could be suggested that compound **3f** might induce cancer cell apoptosis in a dose dependent manner.

## 3. Materials and Methods

### 3.1. Instruments and Materials

Tetrandrine was obtained with purity ≥ 98%. The reagents and solvents were purchased from Adamas (Shanghai, China), J&K Chemical (Chengdu, China), Energy Chemical (Shanghai, China) and other local commercial dealers. All the reagents and solvents were commercially analytical or guaranteed purity products and used without further purification. Column chromatography was performed on silica gel (Qingdao Haiyang Chemical, Qingdao, China 200-300 mesh) using the indicated eluents. Thin-layer (0.25 mm, GF254) chromatography was carried out on silica gel plates (Qingdao Haiyang Chemical, Qingdao, China). ^1^H-NMR spectra were recorded on 600 MHz (Bruker, Boston, MA, USA) and 400 MHz (Varian, Palo Alto, CA, USA) spectrometers in appropriate solvents using TMS as internal standard or the solvent signals as secondary standards and the chemical shifts are shown in δ scales. Multiplicities of NMR signals are designated as s (singlet), d (doublet), t (triplet), br (broad), and m (multiplet, for unresolved lines). ^13^C-NMR spectra were recorded at 150 and 100 MHz. High-resolution mass spectra were obtained by using an ESI-QTOF mass spectrometer (Bruker, Beijing, China). All the NMR spectra can be found in Supplementary Materials (Figures S1–S52). Melting points (uncorrected) were determined on a WRX-4 micro melting point apparatus (Tansoole, Shanghai, China).

### 3.2. Methods of Synthesis

#### 3.2.1. General Procedure for the Preparation of 14-Nitrotetrandrine (**Tet-NO_2_**)

Under the protection of an argon atmosphere, concentrated HNO_3_ (69%, 1.4 mL, 22.4 mmol) was slowly added dropwise into (CH_3_CO)_2_O (2.0 mL, 21.3 mmol) in an ice-salt bath and stirred for 10 min. Then, the tetrandrine (0.7 g, 1.12 mmol) dissolved in dry DCM (4 mL) was added dropwise into the reaction mixture and stirred in an ice-salt bath. TLC was used to monitor reaction. Upon completion, the reaction mixture was quenched with saturated aqueous solution of sodium bicarbonate, extracted with DCM (3 × 15 mL), dried over anhydrous sodium sulfate and filtered. The solvent was removed under reduced pressure. The residue was purified by silica gel chromatography from DCM/MeOH (30/1 v/v, 0.5% TEA) to afford the compound **Tet-NO_2_**. Light yellow amorphous solid, yield: 93%. Mp: 176–177 °C. ^1^H-NMR (400 MHz, CDCl_3_) *δ* 7.42 (1H, s), 7.37 (1H, dd, *J* = 2.0, 8.0 Hz), 7.12 (1H, dd, *J* = 2.4, 8.0 Hz), 6.77 (1H, dd, *J* = 2.8, 8.4 Hz), 6.54 (1H, s), 6.52 (1H, s), 6.30 (1H, s), 6.28 (1H, d, *J* = 2.0 Hz), 5.98 (1H, s), 3.98 (3H, s), 3.91 (1H, dd, *J* = 6.0, 10.8 Hz), 3.75 (3H, s), 3.69–3.63 (1H, m), 3.52–3.49 (2H, m), 3.38 (3H, s), 3.30–3.25 (1H, m), 3.18 (3H, s), 2.96–2.73 (7H, m), 2.63 (3H, s), 2.53 (1H, d, *J* = 12.8 Hz), 2.35 (1H, m), 2.21 (3H, s). ^13^C-NMR (CDCl_3_, 100 MHz) *δ* 152.3, 152.1, 151.5, 148.7, 148.2, 146.5, 144.2, 143.5, 137.5, 136.4, 133.1, 130.5, 130.4, 128.9, 128.1, 127.6, 121.6, 121.4, 121.3, 119.9, 117.2, 112.5, 108.2, 105.8, 63.6, 61.7, 60.3, 56.3, 55.8, 55.7, 45.3, 43.2, 42.8, 41.5, 37.9, 36.8, 25.3, 21.6. HRMS (ESI) calcd. for C_38_H_42_N_3_O_8_: 668.2972 [M + H]^+^, found: 668.2965.

#### 3.2.2. General Procedure for the Preparation of 14-Aminotetrandrine (**Tet-NH_2_**)

To a mixture of **Tet-NO_2_** (400.0 mg, 0.60 mmol) and palladium on carbon (5%, 40 mg) were added analytical methanol (20 mL) and hydrazine hydrate (85%, 0.18 mL, 4.80 mmol). The mixture was stirred at 65 °C for about 4 h before it was filtered by celite under reduced pressure. The filter was quenched with saturated sodium chloride solution, extracted with DCM (5 × 20 mL), dried over anhydrous sodium sulfate and filtered. The solvent was removed under reduced pressure. The crude product was recrystallized from cyclohexane and acetone (2/7, *v*/*v*) to give **Tet-NH_2_**. White amorphous solid, yield: 84%. Mp: 164–166 °C. ^1^H-NMR (400 MHz, CDCl_3_) *δ* 7.28 (1H, d, *J* = 9.6 Hz), 7.18 (1H, dd, *J* = 2.0, 8.0 Hz), 6.60 (1H, dd, *J* = 2.0, 8.4 Hz), 6.50 (1H, s), 6.46 (1H, s), 6.31 (1H, s), 6.29 (1H, s), 6.12 (1H, dd, *J* = 1.6, 8.0 Hz), 5.87 (1H, s), 3.94 (1H, d, *J* = 9.2 Hz), 3.87 (3H, s), 3.80 (1H, dd, *J* = 5.2, 11.2 Hz), 3.73 (3H, s), 3.64 (1H, m), 3.42 (1H, m), 3.35 (3H, s), 3.26 (1H, dd, *J* = 5.2, 12.4 Hz), 3.11 (3H, s), 2.88 (7H, m), 2.61 (3H, s), 2.42 (3H, s), 2.35 (2H, m). ^13^C-NMR (CDCl_3_, 100 MHz) *δ* 156.6, 151.6, 149.4, 148.7, 148.5, 144.2, 142.0, 140.8, 138.0, 133.2, 132.6, 129.3, 128.0, 127.6, 127.4, 122.6, 122.1, 121.3, 120.9, 120.5, 120.2, 112.3, 105.8, 100.6, 64.2, 61.5, 59.9, 56.1, 55.6, 55.5, 44.9, 43.2, 42.3, 40.8, 40.0, 38.7, 24.6, 20.6. HRMS (ESI) calcd. for C_38_H_44_N_3_O_6_: 638.3230 [M + H]^+^, found: 638.3233.

#### 3.2.3. General Procedure for the Preparation of Compounds **1a**–**1k**

To a mixture of **Tet-NH_2_** (100 mg, 0.16 mmol), HOBT (8.47 mg, 0.63 mmol), EDCI (27.3 mg, 0.17 mmol) and Boc-l-amino acid (0.17 mmol, 1.1 eq) was added DCM (2.0 mL) under the protection of argon atmosphere, and stirred at room temperature for 1.5 to 3 h. The reaction mixture was quenched with saturated aqueous solution of sodium bicarbonate, extracted with DCM (3 × 10 mL), dried over anhydrous sodium sulfate and filtered. The solvent was removed under reduced pressure, and the residue was purified by silica gel chromatography eluated with DCM/MeOH (40/1 *v*/*v*, 0.5% TEA) to afford compounds **1a**–**1k**.

*14-((R)-2-(N-(tert-butoxycarbonyl)amino)-3-phenylpropanamido)tetrandrine* (**1a**). White to light yellow amorphous solid, yield: 85%. Mp: 136–137 °C. ^1^H-NMR (600 MHz, CDCl_3_) *δ* 12.20 (s, 1H), 7.58 (s, 1H), 7.36–7.29 (m, 5H), 7.26 (t, *J* = 7.2 Hz, 1H), 7.23 (dd, *J* = 7.8, 2.4 Hz, 1H), 6.63 (dd, *J* = 8.4, 2.4 Hz, 1H), 6.57 (s, 1H), 6.49 (s, 1H), 6.32 (s, 1H), 6.16 (dd, *J* = 8.4, 1.8 Hz, 1H), 5.91 (s, 1H), 5.44 (d, *J* = 12.0 Hz, 1H), 4.47 (m, 1H), 3.94 (d, *J* = 9.0 Hz, 4H), 3.83 (dd, *J* = 10.8, 5.4 Hz, 1H), 3.76 (s, 3H), 3.58 (m, 1H), 3.47 (m, 1H), 3.36 (s, 3H), 3.27 (m, 2H), 3.16–3.08 (m, 5H), 3.01–2.88 (m, 4H), 2.79 (t, *J* = 12.0 Hz, 1H), 2.70 (dd, *J* = 16.2, 5.4 Hz, 1H), 2.62 (s, 3H), 2.49 (dd, *J* = 17.4, 4.2 Hz, 1H), 2.40 (d, *J* = 17.4 Hz, 4H), 1.46 (s, 9H). ^13^C-NMR (150 MHz, CDCl_3_) *δ* 169.2, 155.8, 155.2, 152.2, 149.4, 148.6, 148.1, 145.6, 144.2, 138.2, 136.6, 134.2, 132.9, 131.5, 129.7, 129.6, 128.6, 128.5, 127.8, 127.2, 127.0, 125.8, 121.4, 121.1, 121.1, 120.8, 120.6, 112.3, 106.9, 105.8, 79.6, 77.3, 77.1, 76.8, 64.2, 61.4, 60.1, 56.3, 56.2, 55.8, 55.6, 53.4, 45.1, 43.2, 42.5, 40.7, 40.0, 39.6, 38.9, 29.7, 28.4, 24.8, 20.7. HRMS (ESI) calcd. for C_52_H_61_N_4_O_9_: 885.4429 [M + H]^+^, found 885.4433.

*14-((R)-2-(N-(tert-butoxycarbonyl)amino)-propanamido)*tetrandrine (**1b**). White to light yellow amorphous solid, yield: 78%. Mp: 149–150 °C. ^1^H-NMR (600 MHz, CDCl_3_) *δ* 12.35 (s, 1H), 7.84 (s, 1H), 7.32 (dd, *J* = 7.8, 1.8 Hz, 1H), 7.23 (dd, *J* = 7.8, 2.4 Hz, 1H), 6.61–6.56 (m, 2H), 6.48 (s, 1H), 6.33 (s, 1H), 6.15 (dd, *J* = 8.4, 1.8 Hz, 1H), 5.91 (s, 1H), 5.57 (d, *J* = 7.8 Hz, 1H), 4.31 (m, 1H), 4.02 (d, *J* = 9.0 Hz, 1H), 3.97 (s, 3H), 3.82 (dd, *J* = 11.4, 5.4 Hz, 1H), 3.76 (s, 3H), 3.68 (m, 1H), 3.46 (m, 1H), 3.37 (s, 3H), 3.25 (dd, *J* = 12.0, 5.4 Hz, 1H), 3.16 (dd, *J* = 13.8, 5.4 Hz, 1H), 3.12 (s, 3H), 3.06 (dd, *J* = 15.0, 9.6 Hz, 1H), 3.02–2.86 (m, 3H), 2.78 (t, *J* = 11.8 Hz, 1H), 2.69 (dd, *J* = 16.2, 4.8 Hz, 1H), 2.62 (s, 3H), 2.55–2.49 (m, 4H), 2.45 (d, *J* = 15.0 Hz, 1H), 1.51 (d, *J* = 7.2 Hz, 12H). ^13^C-NMR (150 MHz, CDCl_3_) *δ* 170.8, 156.1, 155.2, 152.2, 149.5, 148.6, 148.4, 145.1, 144.2, 138.3, 134.0, 132.9, 132.2, 129.6, 128.6, 127.8, 127.1, 125.2, 121.5, 121.2, 121.1, 121.0, 120.6, 112.3, 106.2, 105.9, 79.5, 64.2, 61.2, 60.1, 56.2, 55.8, 55.5, 53.4, 50.6, 45.1, 43.1, 42.5, 40.7, 39.9, 38.9, 28.4, 24.8, 20.4. HRMS (ESI) calcd. for C_46_H_57_N_4_O_9_: 809.4117 [M + H]^+^, found 809.4120.

*14-((R)-2-(N-(tert-butoxycarbonyl)amino)-4-methylthio-butylamido)*tetrandrine (**1c**). White to light yellow amorphous solid, yield: 83%. Mp: 133-134 °C. ^1^H-NMR (600 MHz, CDCl_3_) *δ* 12.41 (s, 1H), 7.75 (s, 1H), 7.32 (dd, *J* = 8.1, 2.0 Hz, 1H), 7.23 (dd, *J* = 8.1, 2.5 Hz, 1H), 6.59 (d, *J* = 8.7 Hz, 2H), 6.48 (s, 1H), 6.33 (s, 1H), 6.15 (dd, *J* = 8.4, 2.0 Hz, 1H), 5.91 (s, 1H), 5.50 (d, *J* = 8.2 Hz, 1H), 4.38 (m, 1H), 4.03–3.99 (m, 1H), 3.96 (s, 3H), 3.84 (dd, *J* = 11.1, 5.6 Hz, 1H), 3.76 (s, 3H), 3.67 (m, 1H), 3.52–3.44 (m, 1H), 3.37 (s, 3H), 3.25 (m, 2H), 3.11 (s, 3H), 3.06 (dd, *J* = 14.8, 9.5 Hz, 1H), 3.03–2.88 (m, 3H), 2.78 (t, *J* = 11.8 Hz, 1H), 2.73–2.63 (m, 3H), 2.62 (s, 3H), 2.55–2.50 (m, 4H), 2.44 (d, *J* = 14.8 Hz, 1H), 2.3–2.16 (m, 1H), 2.15 (s, 3H), 2.05–2.00 (m, 1H), 1.47 (s, 9H). ^13^C-NMR (150 MHz, CDCl_3_) *δ* 169.6, 156.0, 155.5, 152.2, 149.4, 148.7, 148.31, 145., 4144.3, 138.3, 134.0, 132.9, 131.9, 131.9, 129.7, 128.5, 127.2, 125.7, 121.5, 121.1, 121.0, 120.7, 112.3, 106.6, 105.9, 79.7, 77.3, 77.1, 76.9, 64.2, 61.1, 60.1, 56.3, 55.8, 55.6, 54.3, 45.0, 43.1, 42.4, 40.8, 39.8, 38.8, 34.1, 30.2, 29.7, 28.4, 24.7, 20.6, 15.8. HRMS (ESI) calcd. for C_48_H_61_N_4_O_9_S: 869.4152 [M + H]^+^, found 869.4154.

*14-((R)-3-methyl-2-(N-(tert-butoxycarbonyl)amino)-amylamido)*tetrandrine (**1d**). White to light yellow amorphous solid, yield: 81%. Mp: 145–146 °C. ^1^H-NMR (600 MHz, CDCl_3_) *δ* 12.16 (s, 1H), 7.72 (s, 1H), 7.33 (dd, *J* = 8.4, 2.4 Hz, 1H), 7.24 (dd, *J* = 7.8, 2.4 Hz, 1H), 6.60 (d, *J* = 7.2 Hz, 2H), 6.49 (s, 1H), 6.34 (s, 1H), 6.15 (dd, *J* = 8.4, 1.8 Hz, 1H), 5.91 (s, 1H), 5.29 (d, *J* = 9.0 Hz, 1H), 4.29 (m, 1H), 4.01 (d, *J* = 9.6 Hz, 1H), 3.96 (s, 3H), 3.85 (dd, *J* = 11.4, 5.4 Hz, 1H), 3.77 (s, 3H), 3.70 (td, *J* = 13.8, 13.2, 4.8 Hz, 1H), 3.53–3.46 (m, 1H), 3.38 (s, 3H), 3.29 (dd, *J* = 12.0, 5.4 Hz, 1H), 3.22 (dd, *J* = 14.4, 6.0 Hz, 1H), 3.12 (s, 3H), 3.08 (dd, *J* = 15.0, 9.6 Hz, 1H), 3.08–2.88 (m, 3H), 2.79 (t, *J* = 12.0 Hz, 1H), 2.72 (dd, *J* = 15.6, 5.4 Hz, 1H), 2.63 (s, 3H), 2.53 (s, 4H), 2.43 (d, *J* = 15.0 Hz, 1H), 1.84 (m, 1H), 1.70 (t, *J* = 7.2 Hz, 2H), 1.46 (s, 9H), 1.07 (d, *J* = 6.6 Hz, 3H), 1.02 (d, *J* = 6.6 Hz, 3H). ^13^C-NMR (150 MHz, CDCl_3_) *δ* 171.1, 156.0, 155.4, 152.2, 149.4, 148.7, 148.3, 145.3, 144.3, 138.3, 133.9, 132.9, 131.9, 129.7, 128.3, 127.3, 125.8, 121.5, 121.3, 121.1, 121.0, 120.6, 112.3, 106.8, 105.9, 79.4, 64.2, 61.4, 60.1, 56.3, 55.8, 55.6, 53.8, 45.0, 43.4, 43.2, 42.3, 40.9, 39.8, 38.9, 29.7, 28.4, 24.8, 24.7, 23.3, 22.7, 20.7. HRMS (ESI) calcd. for C_49_H_63_N_4_O_9_: 851.4590 [M + H]^+^, found 851.4590.

*14-((R)-3-(indolyl-3)-2-(N-(tert-butoxycarbonyl)amino)-propanamido)tetrandrine* (**1e**). White to light yellow amorphous solid, yield: 84%. Mp: 152–153 °C. ^1^H-NMR (600 MHz, CDCl_3_) *δ* 12.06 (s, 1H), 8.16 (s, 1H), 7.72 (d, *J* = 7.8 Hz, 1H), 7.50 (s, 1H), 7.35 (d, *J* = 8.4 Hz, 1H), 7.32 (dd, *J* = 8.4, 2.1 Hz, 1H), 7.22 (dd, *J* = 8.4, 2.4 Hz, 1H), 7.21–7.18 (m, 1H), 7.17–7.11 (m, 2H), 6.64–6.61 (m, 1H), 6.55 (s, 1H), 6.48 (s, 1H), 6.30 (s, 1H), 6.16 (dd, *J* = 8.4, 2.4 Hz, 1H), 5.90 (s, 1H), 5.51 (d, *J* = 8.4 Hz, 1H), 4.59–4.52 (m, 1H), 3.92–3.81 (m, 5H), 3.75 (s, 3H), 3.46 (m, 3H), 3.33 (d, *J* = 18.6 Hz, 4H), 3.28 (dd, *J* = 12.0, 5.4 Hz, 1H), 3.11 (s, 3H), 3.02 (dd, *J* = 13.8, 6.0 Hz, 1H), 2.97–2.83 (m, 4H), 2.79 (t, *J* = 11.4 Hz, 1H), 2.71 (dd, *J* = 15.6, 5.4 Hz, 1H), 2.62 (s, 3H), 2.44–2.34 (m, 2H), 2.14 (s, 3H), 1.48 (s, 9H). ^13^C-NMR (150 MHz, CDCl_3_) *δ* 169.8, 155.8, 155.3, 155.3, 152.1, 149.3, 148.0, 145.5, 144.3, 138.1, 136.2, 132.9, 131.5, 129.7, 127.9, 127.3, 125.9, 125.9, 122.8, 122.1, 121.4, 121.2, 120.8, 120.5, 119.6, 119.1, 112.3, 111.1, 110.9, 110.8, 107.2, 105.8, 79.5, 64.2, 61.3, 60.1, 56.3, 55.8, 55.7, 55.6, 45.0, 43.0, 42.3, 40.3, 39.6, 38.9, 29.7, 29.5, 29.5, 28.4, 24.7, 20.7. HRMS (ESI) calcd. for C_54_H_62_N_5_O_9_: 924.4537 [M + H]^+^, found 924.4542.

*14-((R)-3-hydroxy-2-(N-(tert-butoxycarbonyl)amino)-butylamido)*tetrandrine (**1f**). White to light yellow amorphous solid, yield: 79%. Mp: 163–165 °C. ^1^H-NMR (600 MHz, CDCl_3_) *δ* 12.46 (s, 1H), 7.81 (s, 1H), 7.32 (dd, *J* = 8.4, 2.4 Hz, 1H), 7.23 (dd, *J* = 7.8, 2.4 Hz, 1H), 6.61 (s, 1H), 6.59 (dd, *J* = 8.4, 2.4 Hz, 1H), 6.49 (s, 1H), 6.33 (s, 1H), 6.15 (dd, *J* = 8.4, 2.4 Hz, 1H), 5.90 (s, 1H), 5.60 (d, *J* = 9.0 Hz, 1H), 4.25–4.19 (m, 1H), 4.15–4.11 (m, 1H), 4.01 (d, *J* = 9.0 Hz, 1H), 3.96 (s, 3H), 3.83 (dd, *J* = 11.4, 5.4 Hz, 1H), 3.76 (s, 3H), 3.66 (m, 1H), 3.50–3.45 (m, 1H), 3.38 (s, 3H), 3.26 (dd, *J* = 12.6, 5.4 Hz, 1H), 3.21 (dd, *J* = 12.6, 6.0 Hz, 1H), 3.11 (s, 4H), 3.03–2.86 (m, 4H), 2.79 (t, *J* = 12.0 Hz, 1H), 2.71 (dd, *J* = 16.2, 5.4 Hz, 1H), 2.62 (s, 3H), 2.51 (s, 4H), 2.45 (d, *J* = 14.8 Hz, 1H), 1.48 (s, 9H), 1.34 (d, *J* = 6.0 Hz, 3H). ^13^C-NMR (150 MHz, CDCl_3_) *δ* 169.5, 156.2, 156.1, 152.2, 149.4, 148.6, 148.3, 145.5, 144.3, 138.3, 134.0, 132.9, 131.7, 129.7, 128.5, 127.7, 127.3, 125.8, 121.6, 121.2, 121.0, 120.6, 112.3, 106.7, 105.9, 79.9, 77.3, 77.0, 76.8, 69.3, 64.2, 61.3, 60.1, 59.8, 56.3, 55.8, 55.6, 45.1, 43.3, 42.4, 40.8, 40.0, 38.8, 29.7, 28.4, 24.8, 20.8, 19.7. HRMS (ESI) calcd. for C_47_H_59_N_4_O_10_: 839.4230 [M + H]^+^, found 839.4226.

*14-((R)-3-methyl-2-(N-(tert-butoxycarbonyl)amino)-butylamido)*tetrandrine (**1g**). White to light yellow amorphous solid, yield: 84%. Mp: 143–144 °C. ^1^H-NMR (600 MHz, CDCl_3_) *δ* 12.28 (s, 1H), 7.78 (s, 1H), 7.32 (dd, *J* = 8.1, 1.9 Hz, 1H), 7.24 (dd, *J* = 7.8, 2.4 Hz, 1H), 6.60 (d, *J* = 9.8 Hz, 2H), 6.48 (s, 1H), 6.34 (s, 1H), 6.17–6.13 (m, 1H), 5.91 (s, 1H), 5.42 (d, *J* = 9.0 Hz, 1H), 4.09 (dd, *J* = 9.0, 6.0 Hz, 1H), 4.03 (d, *J* = 9.3 Hz, 1H), 3.96 (s, 3H), 3.82 (dd, *J* = 11.1, 5.5 Hz, 1H), 3.77 (s, 3H), 3.69 (m, 1H), 3.51–3.44 (m, 1H), 3.38 (s, 3H), 3.26 (dd, *J* = 12.3, 5.5 Hz, 1H), 3.21 (dd, *J* = 14.0, 5.9 Hz, 1H), 3.12 (s, 3H), 3.07 (dd, *J* = 14.8, 9.5 Hz, 1H), 3.03–2.85 (m, 3H), 2.79 (t, *J* = 11.8 Hz, 1H), 2.72–2.67 (m, 1H), 2.62 (s, 3H), 2.57–2.49 (m, 4H), 2.44 (d, *J* = 14.8 Hz, 1H), 2.12 (m, 1H), 1.47 (s, 9H), 1.11 (d, *J* = 6.8 Hz, 3H), 1.03 (d, *J* = 6.7 Hz, 3H). ^13^C-NMR (150 MHz, CDCl_3_) *δ* 170.1, 156.1, 156.0, 152.2, 149.5, 148.6, 148.3, 145.2, 144.2, 138.3, 134.1, 132.9, 132.0, 129.7, 128.6, 127.2, 125.6, 121.6, 121.2, 121.2, 121.0, 120.6, 112.3, 106.4, 105.9, 79.3, 64.2, 61.3, 60.3, 60.1, 56.3, 55.8, 55.5, 53.4, 45.1, 43.2, 42.5, 40.9, 39.8, 38.9, 32.9, 28.4, 24.8, 20.6, 19.9, 17.8. HRMS (ESI) calcd. for C_48_H_61_N_4_O_9_: 837.4421 [M + H]^+^, found 837.4433.

*14-((2R,3R)-3-methyl-2-(N-(tert-butoxycarbonyl)amino)-amylamido)*tetrandrine (**1h**). White to light yellow amorphous solid, yield: 79%. Mp: 158–159 °C. ^1^H-NMR (600 MHz, CDCl_3_) *δ* 12.25 (s, 1H), 7.79 (s, 1H), 7.33 (dd, *J* = 7.8, 1.8 Hz, 1H), 7.24 (dd, *J* = 7.8, 2.4 Hz, 1H), 6.60 (d, *J* = 8.4 Hz, 2H), 6.49 (s, 1H), 6.34 (s, 1H), 6.14 (dd, *J* = 8.4, 1.8 Hz, 1H), 5.91 (s, 1H), 5.38 (d, *J* = 9.0 Hz, 1H), 4.12–4.07 (m, 1H), 4.03 (d, *J* = 9.6 Hz, 1H), 3.96 (s, 3H), 3.85 (dd, *J* = 11.4, 5.4 Hz, 1H), 3.77 (s, 3H), 3.69 (m, 1H), 3.52–3.46 (m, 1H), 3.38 (s, 3H), 3.28 (dd, *J* = 12.0, 5.4 Hz, 1H), 3.21 (dd, *J* = 13.8, 6.0 Hz, 1H), 3.12 (s, 3H), 3.08 (dd, *J* = 15.0, 9.6 Hz, 1H), 3.03–2.89 (m, 3H), 2.78 (t, *J* = 12.0 Hz, 1H), 2.72 (dd, *J* = 15.6, 5.4 Hz, 1H), 2.63 (s, 3H), 2.55–2.50 (m, 4H), 2.44 (d, *J* = 15.0 Hz, 1H), 1.87 (m, 1H), 1.66 (m, 1H), 1.46 (s, 9H), 1.26–1.20 (m, 1H), 1.09 (d, *J* = 6.6 Hz, 3H), 0.97 (t, *J* = 7.2 Hz, 3H). ^13^C-NMR (150 MHz, CDCl_3_) *δ* 170.3, 156.1, 155.9, 152.2, 149.4, 148.7, 148.3, 145.2, 144.3, 138.3, 133.9, 132.9, 132.0, 129.7, 128.4, 127.4, 127.3, 125.6, 121.6, 121.3, 121.2, 121.0, 120.6, 112.3, 106.5, 105.9, 79.3, 77.3, 77.1, 76.8, 64.1, 61.3, 60.1, 59.9, 56.3, 55.8, 55.5, 45.0, 43.2, 42.3, 40.9, 39.9, 39.3, 38.9, 29.7, 28.4, 24.5, 20.6, 16.0, 11.6. HRMS (ESI) calcd. for C_49_H_63_N_4_O_9_ [M + H]^+^: 851.4583, found 851.4590.

*14-((R)-3-(S-(tert-butoxycarbonyl)sulfydryl)-propanamido)tetrandrine* (**1i**). White to light yellow amorphous solid, yield: 82%. Mp: 147–149 °C. ^1^H-NMR (600 MHz, CDCl_3_) *δ* 12.48 (s, 1H), 7.75 (s, 1H), 7.32 (dd, *J* = 8.4, 2.4 Hz, 1H), 7.23 (dd, *J* = 7.8, 2.4 Hz, 1H), 6.61–6.57 (m, 2H), 6.49 (s, 1H), 6.33 (s, 1H), 6.16 (dd, *J* = 8.4, 2.4 Hz, 1H), 5.91 (s, 1H), 5.58 (d, *J* = 8.4 Hz, 1H), 4.44 (m, 1H), 4.01 (d, *J* = 9.6 Hz, 1H), 3.96 (s, 3H), 3.82 (dd, *J* = 10.8, 5.4 Hz, 1H), 3.76 (s, 3H), 3.64 (m, 1H), 3.51–3.44 (m, 1H), 3.38 (s, 4H), 3.27–3.19 (m, 3H), 3.12 (s, 3H), 3.05 (dd, *J* = 15.0, 9.6 Hz, 1H), 3.02–2.86 (m, 4H), 2.79 (t, *J* = 12.0 Hz, 1H), 2.70 (dd, *J* = 16.2, 5.4 Hz, 1H), 2.62 (s, 3H), 2.55 (s, 3H), 2.51 (dd, *J* = 16.8, 4.8 Hz, 1H), 2.44 (d, *J* = 14.4 Hz, 1H), 1.50 (s, 9H), 1.47 (s, 9H). ^13^C-NMR (150 MHz, CDCl_3_) *δ* 168.5, 168.1, 156.0, 155.1, 152.18, 149.44, 148.61, 148.24, 145.45, 144.20, 138.25, 134.11, 132.92, 131.73, 129.66, 128.58, 127.77, 127.3, 125.7, 121.5, 121.2, 121.0, 120.9, 120.7, 112.3, 106.9, 105.8, 85.1, 79.8, 64.2, 61.3, 60.1, 56.3, 55.8, 55.6, 54.9, 53.5, 43.1, 42.5, 40.8, 39.9, 38.9, 34.3, 28.4, 28.2, 24.9, 20.7. HRMS (ESI) calcd. for C_51_H_65_N_4_O_11_S: 941.4366 [M + H]^+^, found 941.4365.

*14-((R)-4-(C-(tert-butoxycarbonyl)carboxyl)-2-(N-(tert-butoxycarbonyl)amino)-butylamido)tetrandrine* (**1j**) White to light yellow amorphous solid, yield: 87%. Mp: 131–132 °C. ^1^H-NMR (600 MHz, CDCl_3_) *δ* 12.19 (s, 1H), 7.98 (s, 1H), 7.31 (dd, *J* = 7.8, 1.8 Hz, 1H), 7.23 (dd, *J* = 7.8, 2.4 Hz, 1H), 6.59 (s, 1H), 6.58–6.55 (m, 1H), 6.49 (s, 1H), 6.33 (s, 1H), 6.13 (dd, *J* = 8.4, 1.8 Hz, 1H), 5.90 (s, 1H), 5.29 (d, *J* = 8.4 Hz, 1H), 4.25 (m, 1H), 3.99 (d, *J* = 9.0 Hz, 1H), 3.96 (s, 3H), 3.83 (dd, *J* = 11.2, 5.4 Hz, 1H), 3.77 (s, 3H), 3.62 (td, *J* = 12.6, 3.6 Hz, 1H), 3.53–3.47 (m, 1H), 3.38 (s, 3H), 3.29 (dd, *J* = 12.0, 4.8 Hz, 1H), 3.11 (s, 3H), 3.09–2.88 (m, 5H), 2.78 (t, *J* = 12.0 Hz, 1H), 2.72 (dd, *J* = 15.0, 4.8 Hz, 1H), 2.63 (s, 3H), 2.51 (s, 5H), 2.42 (dd, *J* = 23.6, 12.0 Hz, 2H), 2.32 (m, 1H), 2.07 (m, 1H), 1.51 (s, 9H), 1.45 (s, 9H). ^13^C-NMR (150 MHz, CDCl_3_) *δ* 171.6, 170.4, 156.5, 155.7, 152.2, 149.5, 148.7, 148.4, 144.6, 144.3, 138.3, 133.6, 132.9, 132.9, 129.6, 128.4, 127.1, 124.7, 121.6, 121.4, 120.9, 120.5, 112.3, 106.0, 105.9, 82.1, 79.6, 77.3, 77.1, 76.8, 64.3, 61.1, 60.1, 56.2, 55.8, 55.6, 54.2, 53.4, 45.1, 43.1, 42.4, 40.6, 40.2, 38.9, 34.0, 28.9, 28.4, 28.0, 24.7, 20.7. HRMS (ESI) calcd. for C_53_H_67_N_4_O_13_: 967.4699 [M + H]^+^, found 967.4671.

#### 3.2.4. General Procedure for the Preparation of Compounds **1k** and **1l**

Trifluoroacetic acid (0.1 mL) was slowly added dropwise to a solution of **1k** (160 mg, 0.16 mmol) or **1l** (130 mg, 0.16 mmol) in DCM (2 mL) at 0 °C. After 10 min, the reaction mixture was warmed up to room temperature, and stirred for 0.5 to 1.5 h before the reaction finished. The reaction mixture was quenched with saturated aqueous solution of sodium bicarbonate, extracted with DCM (3 × 10 mL), dried over anhydrous sodium sulfate and filtered. The solvent was removed under reduced pressure. The residue was purified by silica gel chromatography from DCM/MeOH (30/1 v/v, 0.5 % TEA) to afford the pure compounds **1k** and **1l**.

*14-((R)-2-amino-3-phenyl-propanamido)tetrandrine* (**1k**). White amorphous solid, yield: 76%. Mp: 140–141 °C. ^1^H-NMR (600 MHz, CDCl_3_) *δ* 11.83 (s, 1H), 7.74 (s, 1H), 7.37–7.29 (m, 5H), 7.28–7.25 (m, 1H), 7.23 (dd, *J* = 8.4, 2.4 Hz, 1H), 6.63 (dd, *J* = 8.4, 2.4 Hz, 1H), 6.58 (s, 1H), 6.49 (s, 1H), 6.32 (s, 1H), 6.16 (dd, *J* = 8.4, 2.4 Hz, 1H), 5.92 (s, 1H), 4.24 (m, 1H), 4.14 (m, 1H), 3.98 (s, 3H), 3.93 (d, *J* = 9.6 Hz, 1H), 3.84 (dd, *J* = 11.2, 5.4 Hz, 1H), 3.76 (s, 3H), 3.65 (dd, *J* = 7.8, 6.0 Hz, 1H), 3.56–3.42 (m, 2H), 3.37 (s, 3H), 3.27 (dd, *J* = 12.6, 6.0 Hz, 1H), 3.21 (dd, *J* = 13.8, 6.0 Hz, 1H), 3.12 (s, 3H), 3.01–2.87 (m, 6H), 2.79 (t, *J* = 12.0 Hz, 1H), 2.71 (dd, *J* = 16.2, 5.4 Hz, 1H), 2.63 (s, 3H), 2.51–2.45 (m, 1H), 2.41 (d, *J* = 14.4 Hz, 1H), 2.37 (s, 3H). ^13^C-NMR (150 MHz, CDCl_3_) *δ* 173.4, 155.7, 152.1, 149.4, 148.6, 148.0, 145.4, 144.2, 138.3, 137.9, 134.1, 132.9, 131.73, 129.7, 129.4, 128.7, 128.5, 127.7, 127.2, 126.8, 125.8, 121.5, 121.4, 121.2, 120.8, 120.4, 112.4, 107.1, 105.8, 64.2, 61.44, 60.2, 58.2, 56.3, 55.8, 55.6, 45.1, 43.9, 42.5, 42.3, 41.0, 39.9, 38.8, 24.9, 21.0. HRMS (ESI) calcd. for C_47_H_53_N_4_O_7_: 785.3904 [M + H]^+^, found 785.3909.

*14-((R)-2-amino-propanamido)tetrandrine.* (**1l**). White to light yellow amorphous solid, yield: 83%. Mp: 137–139 °C. ^1^H-NMR (600 MHz, CDCl_3_) *δ* 11.96 (s, 1H), 7.88 (s, 1H), 7.32 (dd, *J* = 8.2, 2.2 Hz, 1H), 7.23 (dd, *J* = 7.8, 2.4 Hz, 1H), 6.60 (d, *J* = 6.0 Hz, 2H), 6.49 (s, 1H), 6.33 (s, 1H), 6.15 (dd, *J* = 8.4, 2.4 Hz, 1H), 5.91 (s, 1H), 4.02 (s, 1H), 3.97 (s, 3H), 3.83 (dd, *J* = 10.8, 5.4 Hz, 1H), 3.76 (s, 3H), 3.63 (m, 1H), 3.53 (m, 1H), 3.49–3.43 (m, 1H), 3.38 (s, 3H), 3.26 (dd, *J* = 12.6, 5.4 Hz, 1H), 3.11 (s, 3H), 3.06–2.87 (m, 7H), 2.78 (t, *J* = 11.8 Hz, 1H), 2.73–2.68 (m, 1H), 2.62 (s, 3H), 2.51 (s, 4H), 2.45 (d, *J* = 14.8 Hz, 1H), 1.46 (d, *J* = 7.2 Hz, 3H). ^13^C-NMR (150 MHz, CDCl_3_) *δ* 174.9, 156.0, 152.2, 149.5, 148.6, 148.3, 145.0, 144.2, 138.4, 133.9, 132.9, 132.3, 129.7, 128.5, 127.7, 127.1, 125.2, 121.6, 121.4, 121.0, 120.6, 112.3, 106.5, 105.9, 64.2, 61.2, 60.1, 56.2, 55.8, 55.6, 52.0, 45.0, 43.6, 42.4, 40.9, 40.0, 38.8, 29.7, 24.8, 22.0, 20.8. HRMS (ESI) calcd. for C_41_H_49_N_4_O_7_: 704.3589 [M + H]^+^, found 704.3596.

#### 3.2.5. General Procedure for the Preparation of Compounds **2a**–**3k**

Compound **1l** (100 mg, 0.14 mmol) was dissolved in DCM (2.0 mL), triethylamine (98 %, 4.0 µL, 0.03 mmol) and isocyanate (98%, 0.16 mmol) were added into the solution in turn, and the reaction mixture was stirred for 0.5 to 1.5 h. Upon completion, the solvent was removed under reduced pressure. The residue was purified by silica gel chromatography from DCM/MeOH (50/1 v/v, 0.5 % TEA) to afford the pure compounds **2a**, **2b** and **2c**. The compounds **3a**-**3k** were obtained using the same method.

*14-((R)-2-(3-propylureido)-3-phenylpropanamido)tetrandrine* (**2a**). White amorphous solid, yield: 91%. Mp: 156–157 °C. ^1^H-NMR (600 MHz, CDCl_3_) *δ* 12.39 (s, 1H), 7.52 (s, 1H), 7.33 (d, *J* = 4.8 Hz, 5H), 7.28–7.19 (m, 2H), 6.63 (dd, *J* = 8.4, 2.4 Hz, 1H), 6.56 (s, 1H), 6.49 (s, 1H), 6.32 (s, 1H), 6.17 (dd, *J* = 8.4, 1.8 Hz, 1H), 5.91 (s, 1H), 5.83 (d, *J* = 7.4 Hz, 1H), 4.92 (s, 1H), 4.71 (m, 1H), 3.94 (s, 3H), 3.92 (d, *J* = 9.6 Hz, 1H), 3.84 (dd, *J* = 11.4, 5.4 Hz, 1H), 3.76 (s, 3H), 3.67–3.62 (m, 1H), 3.48 (dd, *J* = 13.8, 8.4 Hz, 1H), 3.37 (s, 3H), 3.26 (dd, *J* = 13.2, 7.2 Hz, 2H), 3.20 (dd, *J* = 13.8, 6.0 Hz, 1H), 3.11 (s, 3H), 3.11–2.87 (m, 7H), 2.80 (t, *J* = 12.0 Hz, 1H), 2.71 (dd, *J* = 15.6, 5.4 Hz, 1H), 2.63 (s, 3H), 2.51 (dd, *J* = 17.4, 4.8 Hz, 1H), 2.42–2.35 (m, 4H), 1.35 (m, 2H), 0.83 (t, *J* = 7.2 Hz, 3H). ^13^C-NMR (150 MHz, CDCl_3_) *δ* 170.5, 157.5, 155.8, 152.1, 149.3 148.64=, 148.1, 145.7, 144.2, 138.1, 133.0, 131.4, 129.7, 129.7, 128.4, 127.4, 126.8, 126.3, 121.4, 121.2, 120.9, 120.8, 120.7, 112.3, 106.9, 105.9, 64.2, 61.4, 60.1, 56.3, 55.7, 55.7, 55.6, 45.1, 43.1, 42.4, 42.1, 40.7, 40.7, 39.5, 39.0, 24.7, 23.4, 20.6, 11.3. HRMS (ESI) calcd. for C_51_H_60_N_5_O_8_: 870.4429 [M + H]^+^, found 870.4436.

*14-((R)-2-(3-propylureido)propanamido)tetrandrine* (**3a**). White amorphous solid, yield: 94%. Mp: 151–152 °C. ^1^H-NMR (600 MHz, CDCl_3_) *δ* 12.62 (s, 1H), 7.76 (s, 1H), 7.32 (dd, *J* = 8.4, 2.4 Hz, 1H), 7.23 (dd, *J* = 7.8, 2.4 Hz, 1H), 6.60 (s, 1H), 6.58 (dd, *J* = 8.4, 2.4 Hz, 1H), 6.48 (s, 1H), 6.33 (s, 1H), 6.15 (dd, *J* = 8.4, 2.4 Hz, 1H), 6.06–5.99 (m, 1H), 5.90 (s, 1H), 5.15–5.06 (m, 1H), 4.55 (m, 1H), 4.01 (d, *J* = 9.6 Hz, *1*H), 3.96 (s, 3H), 3.81 (dd, *J* = 11.4, 5.4 Hz, 1H), 3.76 (s, 3H), 3.68 (m, 1H), 3.45 (m, 1H), 3.37 (s, 3H), 3.23 (m, 2H), 3.11 (s, 3H), 3.05 (dd, *J* = 15.0, 9.6 Hz, 2H), 3.01–2.86 (m, 4H), 2.78 (t, *J* = 12.0 Hz, 1H), 2.72–2.65 (m, 3H), 2.61 (s, 3H), 2.53 (s, 3H), 2.53–2.49 (m, 1H), 2.44 (d, *J* = 15.0 Hz, 1H), 1.55 (d, *J* = 7.2 Hz, 3H), 1.13 (t, *J* = 7.2 Hz, 3H). ^13^C-NMR (150 MHz, CDCl_3_) *δ* 172.3, 157.8, 157.7, 156.0, 152.2, 149.4, 148.6, 148.4, 145.4, 144.2, 138.2, 134.2, 133.0, 132.0, 129.7, 128.6, 127.9, 127.3, 125.8, 125.8, 121.5, 121.3, 121.0, 121.0, 120.6, 112.3, 106.3, 105.9, 77.3, 77.1, 76.8, 64.2, 61.2, 60.1, 56.3, 55.7, 55.6, 50.2, 46.1, 45.1, 43.0, 42.5, 42.1, 40.6, 39.8, 38.8, 24.9, 23.4, 22.7, 21.1, 20.5. HRMS (ESI) calcd. for C_45_H_56_N_5_O_8_: 794.4123 [M + H]^+^, found 794.4117.

*14-((R)-2-(3-butylureido)propanamido)tetrandrine* (**3b**). Light yellow amorphous solid, yield: 92%. Mp: 160–161 °C. ^1^H-NMR (600 MHz, CDCl_3_) *δ* 12.60 (s, 1H), 7.78 (s, 1H), 7.32 (dd, *J* = 8.4, 2.4 Hz, 1H), 7.23 (dd, *J* = 7.8, 2.4 Hz, 1H), 6.61 (s, 1H), 6.59 (dd, *J* = 8.4, 2.4 Hz, 1H), 6.49 (s, 1H), 6.33 (s, 1H), 6.15 (dd, *J* = 8.4, 2.4 Hz, 1H), 5.91 (s, 1H), 5.89 (d, *J* = 7.2 Hz, 1H), 4.89 (s, 1H), 4.55 (m, 1H), 4.02 (d, *J* = 9.0 Hz, 1H), 3.96 (s, 3H), 3.82 (dd, *J* = 11.4, 5.4 Hz, 1H), 3.76 (s, 3H), 3.71–3.66 (m, 1H), 3.47 (m, 1H), 3.38 (s, 3H), 3.27–3.20 (m, 2H), 3.12 (s, 4H), 3.09–2.86 (m, 5H), 2.79 (t, *J* = 12.0 Hz, 1H), 2.72–2.67 (m, 1H), 2.62 (s, 3H), 2.54 (s, 4H), 2.45 (d, *J* = 15.0 Hz, 1H), 1.55 (d, *J* = 7.2 Hz, 3H), 1.34–1.26 (m, 4H), 0.87 (t, *J* = 7.2 Hz, 3H). ^13^C-NMR (150 MHz, CDCl_3_) *δ* 172.1, 157.6, 156.1, 152.2, 149.4, 148.6, 148.4, 145.3, 144.2, 138.2, 134.2, 133.0, 132.03, 129.7, 128.6, 127.8, 127.3, 125.7, 121.5, 121.4, 121.0, 121.0, 120.6, 112.3, 106.3, 105.9, 64.2, 61.2, 60.1, 56.3, 55.7, 55.6, 50.2, 45.1, 43.1, 42.5, 40.7, 40.2, 39.8, 38.9, 32.3, 24.8, 21.1, 20.6, 20.1, 13.8. HRMS (ESI) calcd. for C_46_H_58_N_5_O_8_: 808.4280 [M + H]^+^, found 808.4272.

*14-((R)-2-(3-tert-butylureido)propanamido)tetrandrine* (**3c**). White to light yellow amorphous solid, yield: 90%. Mp: 215–216 °C. ^1^H-NMR (600 MHz, CDCl_3_) *δ* 12.40 (s, 1H), 7.82 (s, 1H), 7.33 (dd, *J* = 8.4, 2.4 Hz, 1H), 7.24 (dd, *J* = 7.8, 2.4 Hz, 1H), 6.60 (s, 1H), 6.60–6.58 (m, 1H), 6.49 (s, 1H), 6.34 (s, 1H), 6.15 (dd, *J* = 8.4, 2.4 Hz, 1H), 5.91 (s, 1H), 5.47 (d, *J* = 7.4 Hz, 1H), 4.51 (m, 1H), 4.45 (s, 1H), 4.01 (d, *J* = 9.0 Hz, 1H), 3.97 (s, 3H), 3.83 (dd, *J* = 10.8, 5.4 Hz, 1H), 3.77 (s, 3H), 3.69 (m, 1H), 3.51–3.45 (m, 1H), 3.37 (s, 3H), 3.30–3.25 (m, 1H), 3.21 (dd, *J* = 13.8, 6.0 Hz, 1H), 3.12 (s, 3H), 3.06 (dd, *J* = 15.0, 9.6 Hz, 1H), 2.95 (m, 3H), 2.79 (t, *J* = 12.0 Hz, 1H), 2.71 (dd, *J* = 15.6, 6.0 Hz, 1H), 2.63 (s, 3H), 2.53 (s, 4H), 2.45 (d, *J* = 15.0Hz, 1H), 1.52 (d, *J* = 7.2 Hz, 3H), 1.33 (s, 9H). ^13^C-NMR (150 MHz, CDCl_3_) *δ* 171.9, 156.7, 156.1, 152.2, 149.4, 148.6, 148.3, 145.2, 144.2, 138.2, 134.1, 132.9, 132.2, 129.7, 128.6, 127.8, 127.3, 125.5, 121.5, 121.2, 121.1, 121.0, 120.6, 112.3, 106.4, 105.9, 64.2, 61.2, 60.1, 56.2, 55.7, 55.5, 50.3, 49.9, 45.1, 43.1, 42.5, 40.7, 39.8, 38.9, 29.5, 24.8, 21.0, 20.5. HRMS (ESI) calcd. for C_46_H_58_N_5_O_8_: 808.4280 [M + H]^+^, found 808.4272.

*14-((R)-2-(3-(p-tolyl)ureido)propanamido)*tetrandrine (**3d**). White amorphous solid, yield: 90%. Mp: 165–167 °C. ^1^H-NMR (600 MHz, CDCl_3_) *δ* 12.71 (s, 1H), 7.73 (s, 1H), 7.33 (dd, *J* = 8.4 2.4 Hz, 1H), 7.27 (s, 1H), 7.23 (dd, *J* = 7.8, 2.4 Hz, 1H), 7.10–7.00 (m, 4H), 6.61 (s, 1H), 6.60–6.53 (m, 2H), 6.49 (s, 1H), 6.33 (s, 1H), 6.15 (dd, *J* = 8.4, 2.4 Hz, 1H), 5.92 (s, 1H), 4.66 (m, 1H), 4.03 (d, *J* = 9.6 Hz, 1H), 3.84 (s, 4H), 3.77 (s, 3H), 3.71 (m, 1H), 3.48 (m, 1H), 3.38 (s, 3H), 3.25 (m, 2H), 3.12 (s, 4H), 3.02–2.87 (m, 3H), 2.79 (t, *J* = 11.4 Hz, 1H), 2.73–2.68 (m, 1H), 2.62 (s, 3H), 2.55 (s, 3H), 2.52 (dd, *J* = 16.8, 4.8 Hz, 1H), 2.46 (d, *J* = 15.0 Hz, 1H), 2.30 (s, 3H), 1.61 (d, *J* = 7.2 Hz, 3H). ^13^C-NMR (150 MHz, CDCl_3_) *δ* 172.0, 156.0, 155.4, 152.2, 149.4, 148.6, 148.5, 145.6, 144.2, 138.2, 136.2, 134.2, 133.0, 131.8, 129.7, 129.6, 128.7, 127.9, 127.3, 125.9, 121.5, 121.3, 121.0, 120.6, 120.5, 112.3, 106.5, 105.9, 64.2, 61.3, 60.1, 56.2, 55.7, 55.6, 50.2, 46.1, 45.1, 43.1, 42.5, 40.7, 39.8, 38.9, 24.9, 21.0, 20.8, 20.5. HRMS (ESI) calcd. for C_49_H_56_N_5_O_8_: 842.4116 [M + H]^+^, found 842.4123.

*14-((R)-2-(3-(4-methoxyphenyl)ureido)propanamido)*tetrandrine (**3e**). White amorphous solid, yield: 90%. Mp: 183–184 °C. ^1^H-NMR (600 MHz, CDCl_3_) *δ* 12.69 (s, 1H), 7.71 (s, 1H), 7.32 (dd, *J* = 8.4, 2.4 Hz, 1H), 7.23 (dd, *J* = 7.8, 2.4 Hz, 1H), 7.10 (t, *J* = 6.0 Hz, 3H), 6.82–6.78 (m, 2H), 6.60 (s, 1H), 6.57 (dd, *J* = 8.4, 2.4 Hz, 1H), 6.49 (s, 1H), 6.41 (d, *J* = 7.8 Hz, 1H), 6.33 (s, 1H), 6.15 (dd, *J* = 8.4, 2.0 Hz, 1H), 5.91 (s, 1H), 4.64 (m, 1H), 4.02 (d, *J* = 9.6 Hz, 1H), 3.85 (s, 3H), 3.84–3.81 (m, 1H), 3.80 (s, 3H), 3.76 (s, 3H), 3.73–3.67 (m, 1H), 3.46 (m, 1H), 3.38 (s, 3H), 3.24 (m, 2H), 3.12 (s, 3H), 3.08 (dd, *J* = 15.0, 9.6 Hz, 1H), 3.01–2.86 (m, 3H), 2.79 (t, *J* = 11.4 Hz, 1H), 2.72–2.66 (m, 1H), 2.62 (s, 3H), 2.54 (s, 3H), 2.53–2.48 (m, 1H), 2.45 (d, *J* = 15.0 Hz, 1H), 1.60 (d, *J* = 6.6 Hz, 3H). ^13^C-NMR (150 MHz, CDCl_3_) *δ* 171.9, 156.0, 155.8, 152.3, 149.4, 148.6, 148.5, 145.5, 144.2, 138.2, 134.2, 133.0, 131.8, 131.4, 129.7, 128.7, 127.9, 127.2, 125.8, 123.3, 121.5, 121.3, 121.0, 120.6, 114.5, 114.4, 112.3, 106.5, 105.9, 64.2, 61.3, 60.1, 56.2, 55.74, 55.6, 55.5, 50.2, 45.1, 43.1, 42.5, 40.7, 39.8, 38.9, 24.9, 21.0, 20.5. HRMS (ESI) calcd. for C_49_H_56_N_5_O_9_: 858.4067 [M + H]^+^, found 858.4073.

*14-((R)-2-(3-(4-(trifluoromethyl)phenyl)ureido)propanamido)tetrandrine* (**3f**). Light yellow amorphous solid, yield: 95%. Mp: 156–157 °C. ^1^H-NMR (600 MHz, CDCl_3_) *δ* 12.93 (s, 1H), 8.09 (s, 1H), 7.62 (s, 1H), 7.36 (dd, *J* = 8.4, 2.4 Hz, 1H), 7.30 (s, 1H), 7.26–7.22 (m, 2H), 6.98 (d, *J* = 8.4 Hz, 2H), 6.66 (s, 1H), 6.59 (dd, *J* = 8.4, 2.4 Hz, 1H), 6.51 (s, 1H), 6.34 (s, 1H), 6.16 (dd, *J* = 8.4, 2.0 Hz, 1H), 5.95 (s, 1H), 4.74 (m, 1H), 4.05 (d, *J* = 9.6 Hz, 1H), 3.85 (dd, *J* = 10.8, 5.4 Hz, 1H), 3.79 (s, 3H), 3.77 (s, 3H), 3.75–3.70 (m, 1H), 3.48 (m, 1H), 3.40 (s, 3H), 3.27 (m, 2H), 3.14 (s, 4H), 3.06–2.87 (m, 4H), 2.80 (t, *J* = 11.4 Hz, 1H), 2.71 (dd, *J* = 16.6, 5.4 Hz, 1H), 2.63 (s, 3H), 2.60–2.57 (m, 3H), 2.56 (d, *J* = 18.0 Hz, 1H), 2.52 (d, *J* = 15.0 Hz, 1H), 1.70 (d, *J* = 7.2 Hz, 3H). ^13^C-NMR (150 MHz, CDCl_3_) *δ* 172.7, 155.6, 154.8, 152.3, 149.3, 148.6, 148.5, 146.5, 144.2, 142.6, 138.2, 134.5, 133.0, 131.1, 129.9, 128.8, 127.9, 127.2, 126.8, 125.8, 125.8, 121.4, 121.1, 121.0, 120.7, 120.6, 117.8, 112.3, 107.4, 105.9, 64.2, 61.6, 60.2, 56.3, 55.8, 55.6, 50.0, 46.1, 45.1, 43.3, 42.5, 40.7, 39.6, 38.9, 24.9, 21.1, 20.6. HRMS (ESI) calcd. for C_49_H_53_F_3_N_5_O_8_: 896.3833 [M + H]^+^, found 896.3841.

*14-((R)-2-(3-(4-(trifluoromethoxy)phenyl)ureido)propanamido)tetrandrine* (**3g**). Light yellow amorphous solid, yield: 91%. Mp: 173–174 °C. ^1^H-NMR (600 MHz, CDCl_3_) *δ* 12.90 (s, 1H), 7.86 (s, 1H), 7.65 (s, 1H), 7.35 (dd, *J* = 8.2, 2.2 Hz, 1H), 7.23 (dd, *J* = 8.1, 2.6 Hz, 1H), 7.04–6.94 (m, 5H), 6.63 (s, 1H), 6.58 (dd, *J* = 8.4, 2.6 Hz, 1H), 6.50 (s, 1H), 6.33 (s, 1H), 6.15 (dd, *J* = 8.4, 2.2 Hz, 1H), 5.93 (s, 1H), 4.71 (m, 1H), 4.04 (d, *J* = 9.4 Hz, 1H), 3.85 (dd, *J* = 11.2, 5.6 Hz, 1H), 3.80 (s, 3H), 3.77 (s, 3H), 3.72 (m, 1H), 3.48 (m, 1H), 3.39 (s, 3H), 3.29–3.21 (m, 2H), 3.13 (s, 4H), 3.03–2.88 (m, 3H), 2.80 (t, *J* = 11.8 Hz, 1H), 2.71 (dd, *J* = 16.1, 5.4 Hz, 1H), 2.63 (s, 3H), 2.57 (s, 3H), 2.54 (dd, *J* = 17.3, 4.7 Hz, 1H), 2.50 (s, 1H), 1.67 (d, *J* = 7.0 Hz, 3H). ^13^C-NMR (150 MHz, CDCl_3_) *δ* 172.6, 155.8, 155.1, 152.3, 149.3, 148.6, 148.5, 146.2, 144.2, 143.8, 138.2, 138.0, 134.3, 133.0, 131.3, 129.8, 128.7, 127.8, 127.2, 126.6, 121.5, 121.4, 121.2, 121.0, 120.7, 120.6, 119.9, 119.7, 112.3, 107.2, 105.9, 64.1, 61.5, 60.1, 56.3, 55.7, 55.6, 50.0, 45.8, 45.0, 43.2, 42.4, 40.7, 39.6, 38.8, 29.7, 24.8, 21.1, 20.6. HRMS (ESI) calcd. for C_49_H_53_F_3_N_5_O_9_: 912.3783 [M + H]^+^, found 912.3790.

*14-((R)-2-(3-(4-fluorophenyl)ureido)propanamido)tetrandrine* (**3h**). Light yellow amorphous solid, yield: 91%. Mp: 139–140 °C. ^1^H-NMR (600 MHz, CDCl_3_) *δ* 12.79 (s, 1H), 7.67 (s, 1H), 7.38 (s, 1H), 7.36–7.33 (m, 1H), 7.25 (dd, *J* = 8.4, 3.0 Hz, 1H), 7.03 (dd, *J* = 9.0, 4.8 Hz, 2H), 6.87 (t, *J* = 8.4 Hz, 2H), 6.68 (d, *J* = 7.2 Hz, 1H), 6.61 (s, 1H), 6.58 (dd, *J* = 8.4, 2.4 Hz, 1H), 6.50 (s, 1H), 6.33 (s, 1H), 6.15 (dd, *J* = 8.4, 2.4 Hz, 1H), 5.93 (s, 1H), 4.67 (m, 1H), 4.03 (d, *J* = 9.6 Hz, 1H), 3.89–3.85 (m, 1H), 3.84 (s, 3H), 3.77 (s, 3H), 3.69 (d, *J* = 15.0 Hz, 1H), 3.53–3.47 (m, 1H), 3.39 (s, 3H), 3.29 (dd, *J* = 7.8, 1.8 Hz, 1H), 3.24 (dd, *J* = 14.4, 6.0 Hz, 1H), 3.13 (s, 3H), 3.09 (dd, *J* = 15.0, 9.6 Hz, 1H), 3.03–2.92 (m, 3H), 2.79 (t, *J* = 11.4 Hz, 1H), 2.73 (d, *J* = 16.8 Hz, 1H), 2.64 (s, 3H), 2.56 (s, 3H), 2.53 (dd, *J* = 18.0, 4.2 Hz, 1H), 2.47 (d, *J* = 14.4 Hz, 1H), 1.64 (d, *J* = 7.2 Hz, 3H). ^13^C-NMR (150 MHz, CDCl_3_) *δ* 179.7, 170.6, 160.5, 156.1, 152.3, 149.4, 148.6, 148.4, 145.5, 144.2, 138.3, 134.2, 132.9, 131.6, 129.7, 128.7, 127.8, 127.4, 127.3, 127.2, 125.7, 121.5, 121.4, 121.0, 1209, 120.6, 117.0, 116.9, 112.3, 106.1, 105.9, 64.2, 61.2, 60.1, 56.3, 55.8, 55.6, 55.1, 46.0, 45.0, 43.3, 42.4, 40.7, 39.9, 38.9, 24.8, 20.6, 20.1. HRMS (ESI) calcd. for C_48_H_53_FN_5_O_8_: 846.3873 [M + H]^+^, found 846.3877.

*14-((R)-2-(3-(4-chlorophenyl)ureido)propanamido)tetrandrine* (**3i**). Light yellow amorphous solid, yield: 91%. Mp: 186–187 °C. ^1^H-NMR (600 MHz, CDCl_3_) *δ* 12.83 (s, 1H), 7.79 (s, 1H), 7.63 (d, *J* = 1.9 Hz, 1H), 7.36 (dd, *J* = 7.8, 2.4 Hz, 1H), 7.28–7.26 (m, 1H), 7.06 (dd, *J* = 9.0, 1.8 Hz, 2H), 7.00 (d, *J* = 7.8 Hz, 1H), 6.92 (dd, *J* = 9.0, 2.4 Hz, 2H), 6.63 (d, *J* = 1.8 Hz, 1H), 6.59 (dd, *J* = 8.4, 2.4 Hz, 1H), 6.50 (s, 1H), 6.34 (s, 1H), 6.16 (dd, *J* = 8.4, 2.4 Hz, 1H), 5.94 (d, *J* = 1.8 Hz, 1H), 4.70 (m, 1H), 4.03 (d, *J* = 9.6 Hz, 1H), 3.85–3.81 (m, 4H), 3.77 (d, *J* = 1.8 Hz, 3H), 3.70 (d, *J* = 16.8 Hz, 1H), 3.50 – 3.45 (m, 1H), 3.39 (s, 3H), 3.25 (m, 2H), 3.16–3.13 (m, 3H), 3.10 (dd, *J* = 13.8, 9.0 Hz, 1H), 2.96 (m, 3H), 2.80 (t, *J* = 12.0 Hz, 1H), 2.70 (dd, *J* = 16.2, 4.8 Hz, 1H), 2.62 (s, 3H), 2.57 (s, 3H), 2.55–2.52 (m, 1H), 2.49 (d, *J* = 15.0 Hz, 1H), 1.66 (d, *J* = 6.6 Hz, 3H). ^13^C-NMR (150 MHz, CDCl_3_) *δ* 172.7, 155.7, 155.1, 152.3, 149.3, 148.7, 148.5, 146.2, 144.2, 138.2, 137.97, 134.3, 133.0, 131.2, 129.8, 128.6, 128.6, 127.7, 127.3, 126.9, 126.6, 121.4, 121.2, 121.0, 120.7, 120.6, 120.0, 112.3, 107.2, 105.9, 64.1, 61.5, 60.1, 56.3, 55.7, 55.6, 50.0, 45.7, 45.0, 43.2, 42.4, 40.7, 39.6, 38.8, 24.8, 21.1, 20.6. HRMS (ESI) calcd. for C_48_H_53_Cl_3_N_5_O_8_: 852.3568 [M + H]^+^, found 852.3577.

*14-((R)-2-(3-(4-nitrophenyl)ureido)propanamido)tetrandrine* (**3j**). Yellow amorphous solid, yield: 94%. Mp: 175–176 °C. ^1^H-NMR (600 MHz, CDCl_3_) *δ* 13.03 (s, 1H), 8.43 (s, 1H), 7.93 (d, *J* = 9.0 Hz, 2H), 7.52 (s, 1H), 7.48 (d, *J* = 7.8 Hz, 1H), 7.40 (d, *J* = 7.4 Hz, 1H), 7.32 (dd, *J* = 8.4, 2.4 Hz, 1H), 6.89 (d, *J* = 8.4 Hz, 2H), 6.71 (s, 1H), 6.60 (dd, *J* = 8.4, 2.4 Hz, 1H), 6.52 (s, 1H), 6.35 (s, 1H), 6.19–6.15 (m, 1H), 5.98 (s, 1H), 4.77 (p, *J* = 7.2, 6.6 Hz, 1H), 4.05 (d, *J* = 9.6 Hz, 1H), 3.88 (dd, *J* = 10.4, 5.4 Hz, 1H), 3.79 (s, 3H), 3.77 (s, 3H), 3.73 (dd, *J* = 12.6, 3.6 Hz, 1H), 3.50 (m, 1H), 3.41 (s, 3H), 3.28 (m, 2H), 3.18–3.11 (m, 4H), 3.08–3.01 (m, 1H), 2.95 (m, 2H), 2.81 (t, *J* = 11.4 Hz, 1H), 2.73 (dd, *J* = 16.2, 5.4 Hz, 1H), 2.65 (s, 3H), 2.60 (s, 3H), 2.56 (d, *J* = 14.4 Hz, 2H), 1.72 (d, *J* = 7.2 Hz, 3H). ^13^C-NMR (150 MHz, CDCl_3_) *δ* 172.8, 155.2, 154.2, 152.4, 149.3, 148.6, 148.5, 147.0, 145.8, 144.1, 141.7, 138.2, 134.8, 133.1, 130.6, 130.1, 128.8, 127.9, 127.3, 127.1, 124.9, 121.4, 121.3, 120.7, 120.7, 120.2, 117.1, 112.3, 107.7, 105.8, 77.3, 77.1, 76.8, 64.1, 61.7, 60.3, 56.3, 55.8, 55.6, 49.9, 46.0, 45.1, 43.3, 42.5, 40.8, 39.4, 38.8, 24.9, 21.2, 20.6. HRMS (ESI) calcd. for C_48_H_53_N_6_O_10_: 873.3811[M + H]^+^, found 873.3818.

*14-((R)-2-(3-(1-naphthalenyl)ureido)propanamido)tetrandrine* (**3k**). Light yellow amorphous solid, yield: 90%. Mp: 164–166 °C. ^1^H-NMR (600 MHz, CDCl_3_) δ 12.63 (s, 1H), 8.09 (d, *J* = 8.4 Hz, 1H), 7.89–7.84 (m, 2H), 7.76–7.71 (m, 2H), 7.59 (s, 1H), 7.50 (t, *J* = 7.8 Hz, 1H), 7.48–7.45 (m, 1H), 7.40 (t, *J* = 7.8 Hz, 1H), 7.31 (dd, *J* = 8.4, 2.4 Hz, 1H), 7.20 (dd, *J* = 8.4, 2.4 Hz, 1H), 6.81–6.69 (m, 1H), 6.51 (dd, *J* = 8.4, 2.4 Hz, 1H), 6.47 (d, *J* = 5.4 Hz, 2H), 6.28 (s, 1H), 6.11 (dd, *J* = 8.4, 2.4 Hz, 1H), 5.88 (s, 1H), 4.67 (m, 1H), 3.95 (d, *J* = 9.6 Hz, 1H), 3.81 (dd, *J* = 11.4, 5.4 Hz, 1H), 3.76 (s, 3H), 3.64 (s, 3H), 3.60 (m, 1H), 3.46 (m, 1H), 3.36 (s, 3H), 3.24 (dd, *J* = 12.5, 5.4 Hz, 1H), 3.10 (s, 3H), 3.07 (dd, *J* = 14.4, 6.0 Hz, 1H), 2.99 (dd, *J* = 14.4, 9.6 Hz, 1H), 2.95–2.85 (m, 2H), 2.81–2.74 (m, 2H), 2.68 (dd, *J* = 15.6, 5.4 Hz, 1H), 2.61 (s, 3H), 2.41 (s, 3H), 2.38–2.32 (m, 2H), 1.61 (d, *J* = 7.2 Hz, 3H). ^13^C-NMR (150 MHz, CDCl_3_) *δ* 171.7, 156.4, 156.4, 156.1, 152.2, 149.4, 148.6, 148.2, 145.2, 144.2, 138.2, 134.4, 134.0, 133.7, 132.9, 131.8, 129.6, 128.6, 128.3, 127.,8 127.2, 126.1, 126.1, 125.9, 125.6, 125.4, 122.0, 121.6, 121.2, 121.0, 120.9, 120.6, 112.3, 106.1, 105.9, 64.2, 61.2, 60.0, 55.9, 55.7, 55.5, 50.4, 46.0, 45.1, 43.0, 42.5, 40.5, 39.8, 38.9, 24.8, 21.0, 20.4. HRMS (ESI) calcd. for C_52_H_56_N_5_O_8_: 878.4115 [M + H]^+^, found 878.4123.

### 3.3. Cell Lines and Cell Culture

Human leukemic cell lines (HEL and K562) and breast cell line MDA-MB-231 were obtained from the University of Toronto (Toronto, ON, Canada). Cells cultured in RPMI (HEL and K562) or DMEM (MDA-MB-231) medium (high glucose) supplemented with 5% fetal bovine serum FBS (HyClone, GE Healthcare, Sydney, Australia) and maintained in a humidified incubator of 5% CO_2_ at 37 °C. When the growing cells reached approximately 70-90% confluence, they were treated with **3f**.

### 3.4. In Vitro Cytotoxicity Assay

The cells were cultured in 96-wells plates as density of 1 × 10^4^/well. The plates were incubated for 12 h to allow cell to adapt growing circumstance before the test compounds were added. After the adding of compounds in different doses, the cells were incubated for another two days. Then, each well was added with 20 μL diphenyltetrazolium bromide (MTT) and incubated for 4 h, medium removed and 200 μL of dimethyl sulfoxide (DMSO) was added. The IC_50_ was detected by measuring the absorbance at 490 nm on a plate reader (BioTek, Winooski, VT, USA). All experiments were in triplicates and repeated at least three times.

### 3.5. Cell Growth Curve Assay

The compound **3f** was prepared to original solution (20 µM) by DMSO and stored at −20 °C. The human leukemic cell line HEL was cultured in 96-wells plates as density of 1 × 10^4^/well. The plates were incubated for 12 h to allow cells to adapt growing circumstance. Cells then treated with **3f** for 12 h, 24 h, 48 h and 72 h. The cell viability was measured by the MTT method.

### 3.6. Apoptosis Analysis by Annexin V and Propidium Iodide STAINING

HEL cells (3 × 10^5^/well) were cultured in 6 well-plates and treated with **3f** or DMSO as a vehicle control for 24 h. The treated cells were gathered and washed with cold PBS for three times, then redistributed in binding buffer and stained with annexin V and PI, according to manufacturer instruction (BD Biosciences, Franklin Lakes, NJ, USA). Apoptotic cells were analyzed by flow cytometer (ACEA Biosciences Inc, San Diego, CA, USA).

### 3.7. Cell Cycle Analysis by Flow Cytometry

HEL cells (3 × 10^5^/well) were cultured in 6 well-plates and treated with **3f** or DMSO. The treated cells were collected and washed with cold PBS, then dealt with iced 70% ethanol and stored at 4 °C overnight. After that, the cells were centrifuged and washed with PBS for three times, then redistributed in PBS (0.5 mL) containing 100 μg/mL RNase and 50 μg/mL PI. After it was let sit for 1 h in the dark at 37 °C, the cellular DNA content was analyzed by flow cytometry.

### 3.8. Statistical Analysis

The experimental data for all in vitro anticancer experiments were repeated in triplicates at least in three independent times. The t-test was used to determine statistical differences between treated groups and controls, and *P* < 0.05** was considered statistically significant. The values were presented as mean ± SD of the number of experiments. The significance level was calculated using one-way analysis of variance to assess the differences between experimental groups.

## 4. Conclusions

In conclusion, twenty-four tetrandrine derivatives were designed and synthesized. All the derivatives were obtained efficiently under mild reaction conditions. The anti-cancer activity tests of these derivatives against the HEL, K562 and MDA-MB-231 cell lines showed that they exhibited better inhibitory effects than the original compound tetrandrine and the positive control vinblastine. Among these derivatives, compounds **3f** and **3g** showed the strongest cytotoxic effect against the HEL cell line, with IC_50_ values of 0.23 µM and 0.26 µM, which were 85-fold and 24-fold lower than those of tetrandrine, and 65-fold and 36-fold lower than those of vinblastine. Meanwhile, the preliminary mechanistic study results exhibited that compound **3f** could induce cell cycle arrest in the G1/S phase of the HEL cell line. **3f** could also induced HEL cell death through apoptosis. The results thus showed that **3f** could be a potential agent for the treatment of leukemia, but further mechanistic and toxicologic researches should be performed to confirm this.

## Figures and Tables

**Table 1:** The yields and IC_50_ values of **1a**–**1m, 2a**–**2c, 3a**–**3k** against MDA-MB-231, HEL and K562 cell lines.

Compounds	Yield (%)	IC_50_ (µM)		
		MDA-MB-231	HEL	K562
**1a**	85	2.867 ± 0.237	1.941 ± 0.094	1.87 ± 0.061
**1b**	78	5.182 ± 0.449	4.383 ± 0.306	4.900 ± 0.301
**1c**	83	2.206 ± 0.081	0.631 ± 0.059	0.392 ± 0.337
**1d**	81	2.374 ± 0.192	1.864 ± 0.177	0.793 ± 0.032
**1e**	84	2.921 ± 0.221	2.453 ± 0.119	2.590 ± 0.201
**1f**	79	4.758 ± 0.257	2.969 ± 0.255	4.677 ± 0.442
**1g**	84	4.514 ± 0.380	2.410 ± 0.189	2.263 ± 0.019
**1h**	79	2.137 ± 0.169	0.821 ± 0.030	2.421 ± 0.107
**1i**	82	3.934 ± 0.229	2.288 ± 0.176	2.749 ± 0.209
**1j**	87	5.652 ± 0.405	5.386 ± 0.477	3.494 ± 0.253
**1k**	76	4.949 ± 0.398	2.233 ± 0.116	2.081 ± 0.117
**1l**	83	5.747 ± 0.548	4.716 ± 0.231	5.183 ± 0.227
**2a**	91	1.118 ± 0.049	1.171 ± 0.068	1.616 ± 0.108
**3a**	94	0.812 ± 0.090	3.369 ± 0.228	15.025 ± 1.036
**3b**	92	1.088 ± 0.037	1.467 ± 0.136	8.726 ± 0.802
**3c**	90	5.606 ± 0.500	3.273 ± 0.307	6.734 ± 0.638
**3d**	90	4.499 ± 0.443	1.507 ± 0.118	4.214 ± 0.366
**3e**	90	9.091 ± 0.840	1.878 ± 0.174	6.822 ± 0.674
**3f**	95	1.119 ± 0.049	0.230 ± 0.019	2.887 ± 0.260
**3g**	91	1.066 ± 0.105	0.261 ± 0.070	2.943 ± 0.020
**3h**	91	1.725 ± 0.137	0.386 ± 0.058	5.037 ± 0.402
**3i**	91	1.271 ± 0.106	0.940 ± 0.270	3.095 ± 0.291
**3j**	94	1.401 ± 0.106	1.362 ± 0.134	3.560 ± 0.126
**3k**	90	2.256 ± 0.204	1.762 ± 0.146	4.136 ± 0.327
**vinblastine**		17.744 ± 0.653	15.980 ± 1.023	9.494 ± 0.750
**tetrandrine**		18.452 ± 1.271	19.742 ± 1.301	6.433 ± 0.806
**fangchinoline**		58.607 ± 1.765	22.709 ± 1.353	5.935 ± 0.771

Note: Result of MTT assays after 48 h of drug treatment; the values are averaged for at least three independent experiments; variation ± 10%.

## Supplementary Materials

The following are available online, Figure S1. ^1^H-NMR Spectra of Tet-NO2 in CDCl_3_, Figure S2. ^1^H-NMR Spectra of Tet-NH2 in CDCl_3_, Figure S3. ^1^H-NMR Spectra of 1a in CDCl_3_, Figure S4. ^1^H-NMR Spectra of 1b in CDCl_3_, Figure S5. ^1^H-NMR Spectra of 1c in CDCl_3_, Figure S6. ^1^H-NMR Spectra of 1d in CDCl_3_, Figure S7. ^1^H-NMR Spectra of 1e in CDCl_3_, Figure S8. ^1^H-NMR Spectra of 1f in CDCl_3_, Figure S9. ^1^H-NMR Spectra of 1g in CDCl_3_, Figure S10. ^1^H-NMR Spectra of 1h in CDCl_3_, Figure S11. ^1^H-NMR Spectra of 1i in CDCl_3_, Figure S12. ^1^H-NMR Spectra of 1j in CDCl_3_, Figure S13. ^1^H-NMR Spectra of 1k in CDCl_3_, Figure S14. ^1^H-NMR Spectra of 1l in CDCl_3_, Figure S15. ^1^H-NMR Spectra of 2a in CDCl_3_, Figure S16. ^1^H-NMR Spectra of 3a in CDCl_3_, Figure S17. ^1^H-NMR Spectra of 3b in CDCl_3_, Figure S18. ^1^H-NMR Spectra of 3c in CDCl3, Figure S19. ^1^H-NMR Spectra of 3d in CDCl_3_, Figure S20. ^1^H-NMR Spectra of 3e in CDCl_3_, Figure S21. ^1^H-NMR Spectra of 3f in CDCl_3_, Figure S22. ^1^H-NMR Spectra of 3g in CDCl_3_, Figure S23. ^1^H-NMR Spectra of 3h in CDCl_3_, Figure S24. ^1^H-NMR Spectra of 3i in CDCl_3_, Figure S25. ^1^H-NMR Spectra of 3j in CDCl_3_, Figure S26. ^1^H-NMR Spectra of 3k in CDCl_3_, Figure S27. ^13^C-NMR Spectra of Tet-NO2 in CDCl_3_, Figure S28. ^13^C-NMR Spectra of Tet-NH2 in CDCl_3_, Figure S29. ^13^C-NMR Spectra of 1a in CDCl_3_, Figure S30. ^13^C-NMR Spectra of 1b in CDCl_3_, Figure S31. ^13^C-NMR Spectra of 1c in CDCl_3_, Figure S32. ^13^C-NMR Spectra of 1d in CDCl_3_, Figure S33. ^13^C-NMR Spectra of 1e in CDCl_3_, Figure S34. ^13^C-NMR Spectra of 1f in CDCl_3_, Figure S35. ^13^C-NMR Spectra of 1g in CDCl_3_, Figure S36. ^13^C-NMR Spectra of 1h in CDCl_3_, Figure S37. ^13^C-NMR Spectra of 1i in CDCl_3_, Figure S38. ^13^C-NMR Spectra of 1j in CDCl_3_, Figure S39. ^13^C-NMR Spectra of 1k in CDCl_3_, Figure S40. ^13^C-NMR Spectra of 1l in CDCl_3_, Figure S41. ^13^C-NMR Spectra of 2a in CDCl_3_, Figure S42. ^13^C-NMR Spectra of 3a in CDCl_3_, Figure S43. ^13^C-NMR Spectra of 3b in CDCl_3_, Figure S44. ^13^C-NMR Spectra of 3c in CDCl_3_, Figure S45. ^13^C-NMR Spectra of 3d in CDCl_3_, Figure S46. ^13^C-NMR Spectra of 3e in CDCl_3_, Figure S47. ^13^C-NMR Spectra of 3f in CDCl_3_, Figure S48. ^13^C-NMR Spectra of 3g in CDCl_3_, Figure S49. ^13^C-NMR Spectra of 3h in CDCl_3_, Figure S50. ^13^C-NMR Spectra of 3i in CDCl_3_, Figure S51. ^13^C-NMR Spectra of 3j in CDCl_3_, Figure S52. ^13^C-NMR Spectra of 3k in CDCl_3_.

## References

[1] Bray F., Ferlay J., Soerjomataram I., Siegel R.L., Torre L.A., Jemal A. (2018). Global cancer statistics 2018: GLOBOCAN estimates of incidence and mortality worldwide for 36 cancers in 185 countries. CA Cancer J. Clin..

[2] Miller K.D., Siegel R.L., Lin C.C., Mariotto A.B., Kramer J.L., Rowland J.H., Stein K.D., Alteri R., Jemal A. (2016). Cancer treatment and survivorship statistics, 2016. CA Cancer J. Clin..

[3] Chabner B.A., Roberts T.G. (2005). Timeline: Chemotherapy and the war on cancer. Nat. Rev. Cancer.

[4] Sternberg C.N., Donat S.M., Bellmunt J., Millikan R.E., Stadler W., De Mulder P., Sherif A., von der Maase H., Tsukamoto T., Soloway M.S. (2007). Chemotherapy for bladder cancer: Treatment guidelines for neoadjuvant chemotherapy, bladder preservation, adjuvant chemotherapy, and metastatic cancer. Urology.

[5] Zagouri F., Peroukidis S., Tzannis K., Kouloulias V., Bamias A. (2015). Hellenic Genito-Urinary Cancer, G., Current clinical practice guidelines on chemotherapy and radiotherapy for the treatment of non-metastatic muscle-invasive urothelial cancer: A systematic review and critical evaluation by the Hellenic Genito-Urinary Cancer Group (HGUCG). Crit. Rev. Oncol. Haematol..

[6] Newman D.J., Cragg G.M., Snader K.M. (2003). Natural products as sources of new drugs over the period 1981-2002. J. Nat. Prod..

[7] Mann J. (2002). Natural products in cancer chemotherapy: Past, present and future. Nat. Rev. Cancer.

[8] Cragg G.M., Newman D.J. (2005). Plants as a source of anti-cancer agents. J. Ethnopharmacol..

[9] Surh Y.J. (2003). Cancer chemoprevention with dietary phytochemicals. Nat. Rev. Cancer.

[10] Chikara S., Nagaprashantha L.D., Singhal J., Horne D., Awasthi S., Singhal S.S. (2018). Oxidative stress and dietary phytochemicals: Role in cancer chemoprevention and treatment. Cancer lett..

[11] Fabricant D.S., Farnsworth N.R. (2001). The value of plants used in traditional medicine for drug discovery. Environ. Health perspect..

[12] Bhagya N., Chandrashekar K.R. (2016). Tetrandrine--A molecule of wide bioactivity. Phytochemistry.

[13] Liu T., Zhang Z., Yu C., Zeng C., Xu X., Wu G., Huang Z., Li W. (2017). Tetrandrine antagonizes acute megakaryoblastic leukaemia growth by forcing autophagy-mediated differentiation. Br. J. Pharmacol..

[14] Wong V.K.W., Zeng W., Chen J., Yao X.J., Leung E.L.H., Wang Q.Q., Chiu P., Ko B.C.B., Law B.Y.K. (2017). Tetrandrine, an activator of autophagy, induces autophagic cell death via PKC-alpha inhibition and mTOR-dependent mechanisms. Front. Pharmacol..

[15] Meng L.H., Zhang H., Hayward L., Takemura H., Shao R.G., Pommier Y. (2004). Tetrandrine induces early G1 arrest in human colon carcinoma cells by down-regulating the activity and inducing the degradation of G1-S-specific cyclin-dependent kinases and by inducing p53 and p21Cip1. Cancer Res..

[16] Xiao W., Jiang Y., Men Q., Yuan L., Huang Z., Liu T., Li W., Liu X. (2015). Tetrandrine induces G1/S cell cycle arrest through the ROS/Akt pathway in EOMA cells and inhibits angiogenesis in vivo. Int. J. Oncol..

[17] Lee J.H., Kang G.H., Kim K.C., Kim K.M., Park D.I., Choi B.T., Kang H.S., Lee Y.T., Choi Y.H. (2002). Tetrandrine-induced cell cycle arrest and apoptosis in A549 human lung carcinoma cells. Int. J. Oncol..

[18] Li X., Su B., Liu R., Wu D., He D. (2011). Tetrandrine induces apoptosis and triggers caspase cascade in human bladder cancer cells. J. Surg. Res..

[19] Wang H., Liu T., Li L., Wang Q., Yu C., Liu X., Li W. (2015). Tetrandrine is a potent cell autophagy agonist via activated intracellular reactive oxygen species. Cell Biosci..

[20] Liu W., Kou B., Ma Z.K., Tang X.S., Lv C., Ye M., Chen J.Q., Li L., Wang X.Y., He D.L. (2015). Tetrandrine suppresses proliferation, induces apoptosis, and inhibits migration and invasion in human prostate cancer cells. Asian J. Androl..

[21] Xu W.L., Shen H.L., Ao Z.F., Chen B.A., Xia W., Gao F., Zhang Y.N. (2006). Combination of tetrandrine as a potential-reversing agent with daunorubicin, etoposide and cytarabine for the treatment of refractory and relapsed acute myelogenous leukemia. Leuk. Res..

[22] Fu L.W., Zhang Y.M., Liang Y.J., Yang X.P., Pan Q.C. (2002). The multidrug resistance of tumour cells was reversed by tetrandrine in vitro and in xenografts derived from human breast adenocarcinoma MCF-7/adr cells. Eur. J. Cancer.

[23] Sun Y.F., Wink M. (2014). Tetrandrine and fangchinoline, bisbenzylisoquinoline alkaloids from Stephania tetrandra can reverse multidrug resistance by inhibiting P-glycoprotein activity in multidrug resistant human cancer cells. Phytomedicine.

[24] Wu C.Z., Lai L., Hu X., Lei R.R., Yang Y.F. (2013). Synthesis and antitumor activity of tetrandrine derivatives. J. Asian Nat. Prod. Res..

[25] Niu N., Qu T., Xu J., Lu X., Bodwell G.J., Zhao Z. (2019). Synthesis of 5-alkynyltetrandrine derivatives and evaluation of their anticancer activity on A549 cell lines. Anticancer Agents Med. Chem..

[26] Wei X., Qu T.L., Yang Y.F., Xu J.F., Li X.W., Zhao Z.B., Guo Y.W. (2016). Design and synthesis of new tetrandrine derivatives and their antitumor activities. J. Asian Nat. Prod. Res..

[27] Shi C., Ahmad Khan S., Wang K., Schneider M. (2015). Improved delivery of the natural anticancer drug tetrandrine. Int. J. Pharm..

[28] Hagiwara M., Adachi-Akahane S., Nagao T. (2003). High-affinity binding of [3H] DTZ323 to the diltiazem-binding site of L-type Ca^2+^ channels. Eur. J. Pharm..

[29] Lan J.J., Wang N., Huang L., Liu Y.Z., Ma X.P., Lou H.Y., Chen C., Feng Y.B., Pan W.D. (2017). Design and synthesis of novel tetrandrine derivatives as potential anti-tumor agents against human hepatocellular carcinoma. Eur. J. Med. Chem..

[30] Lan J.J., Huang L., Lou H.Y., Chen C., Liu T.J.J., Hu S.C., Yao Y., Song J.R., Luo J., Liu Y.Z. (2018). Design and synthesis of novel C14-urea-tetrandrine derivatives with potent anti-cancer activity. Eur. J. Med. Chem..

[31] Song J.R., Lan J.J., Chen C., Hu S.C., Song J.R., Liu W., Zeng X.Y., Lou H.Y., Ben-David Y., Pan W.D. (2018). Design, synthesis and bioactivity investigation of tetrandrine derivatives as potential anti-cancer agents. Med. Chem. Comm..

[32] Liu R., Wang S., Fang S., Wang J., Chen J., Huang X., He X., Liu C. (2016). Liquid Crystalline Nanoparticles as an Ophthalmic Delivery System for Tetrandrine: Development, Characterization, and In Vitro and In Vivo Evaluation. Nanoscale Res. Lett..

[33] Tian Y., Yin H., Xu H. (2016). Enhanced Pro-Apoptotic Effect of Tetrandrine Loaded Nanoparticles Against Osteosarcoma Cells. Curr. Drug Deliv..

[34] Vale N., Ferreira A., Matos J., Fresco P., Gouveia M.J. (2018). Amino acids in the development of prodrugs. Molecules.

[35] Gonzalez D.E., Covitz K.M., Sadee W., Mrsny R.J. (1998). An oligopeptide transporter is expressed at high levels in the pancreatic carcinoma cell lines AsPc-1 and Capan-2. Cancer Res..

[36] Buckley S.T., Fischer S.M., Fricker G., Brandl M. (2012). In vitro models to evaluate the permeability of poorly soluble drug entities: Challenges and perspectives. Eur. J. Pharm. Sci..

[37] Nakanishi T., Tamai I., Takaki A., Tsuji A. (2000). Cancer cell-targeted drug delivery utilizing oligopeptide transport activity. Int. J. Cancer.

[38] Vig B.S., Lorenzi P.J., Mittal S., Landowski C.P., Shin H.C., Mosberg H.I., Hilfinger J.M., Amidon G.L. (2003). Amino acid ester prodrugs of floxuridine: Synthesis and effects of structure, stereochemistry, and site of esterification on the rate of hydrolysis. Pharm. Res..

[39] Landowski C.P., Lorenzi P.L., Song X., Amidon G.L. (2006). Nucleoside ester prodrug substrate specificity of liver carboxylesterase. J. Pharm. Exp. Ther..

[40] Vig B.S., Huttunen K.M., Laine K., Rautio J. (2013). Amino acids as promoieties in prodrug design and development. Adv. Drug Deliv. Rev..

[41] Diaz-Padilla I., Siu L.L. (2011). Brivanib alaninate for cancer. Expert Opin. Investig. Drugs..

[42] Li H.Q., Lv P.C., Yan T., Zhu H.L. (2009). Urea derivatives as anticancer agents. Anticancer Agents Med. Chem..

[43] Liu Y.Z., Xia B., Lan J.J., Hu S.C., Huang L., Chen C., Zeng X.Y., Lou H.Y., Lin C.H., Pan W.D. (2017). Design, synthesis and anticancer evaluation of fangchinoline derivatives. Molecules.

[44] Yang J., Hu S.C., Wang C.L., Song J.R., Chen C., Fan Y.H., Ben-David Y., Pan W.D. (2020). Fangchinoline derivatives induce cell cycle arrest and apoptosis in human leukemia cell lines via suppression of the PI3K/AKT and MAPK signaling pathway. Eur. J. Med. Chem..

