# Supplementary material for: Design, Synthesis of Novel Tetrandrine-14-l-Amino Acid and Tetrandrine-14-l-Amino Acid-Urea Derivatives as Potential Anti-Cancer Agents
Source: Molecules. 2020 Apr 9;25(7):1738. doi: 10.3390/molecules25071738 (PMC7180913; doi:10.3390/molecules25071738)
Supplement: Supplementary file 1 [file molecules-25-01738-s001.pdf]

Supplementary Material

Design, Synthesis of Novel Tetrandrine-14-L-Amino Acid and Tetrandrine-14-L-Amino Acid-Urea Derivatives as Potential Anti-cancer Agents

Sheng-Cao Hu [1,2,3,†], Jin Yang [1,3,†], Chao Chen [2,3], Jun-Rong Song [2,3,*] and Wei-Dong Pan [1,2,3,*]

[1] College of Pharmacy, Zunyi Medical University, Zunyi 563000, PR China; hushengcao0221@163.com (S.-C.H.); jinyangtrcwsys@sina.com (J.Y.)

[2] State Key Laboratory of Functions and Applications of Medicinal Plants, Guizhou Medical University, Guiyang 550014, PR China; cc283818640@163.com

[3] The Key Laboratory of Chemistry for Natural Products of Guizhou Province and Chinese Academy of Sciences, Guiyang 550014, PR China

* Correspondence: wdpan@163.com (W.-D.P.), 18275365116@163.com (J.-R.S.), Tel: +86 18985130307 (W.-D.P.)

† These authors contributed equally to this work.

Table of contents

1. ^{1}H-NMR Spectra of **Tet-NO$_2$**, **Tet-NH$_2$** and **1a-3k** 2

2. ^{13}C-NMR Spectra of **Tet-NO$_2$**, **Tet-NH$_2$** and **1a-3k** 15

1. ^{1}H-NMR Spectra of 1a-3k

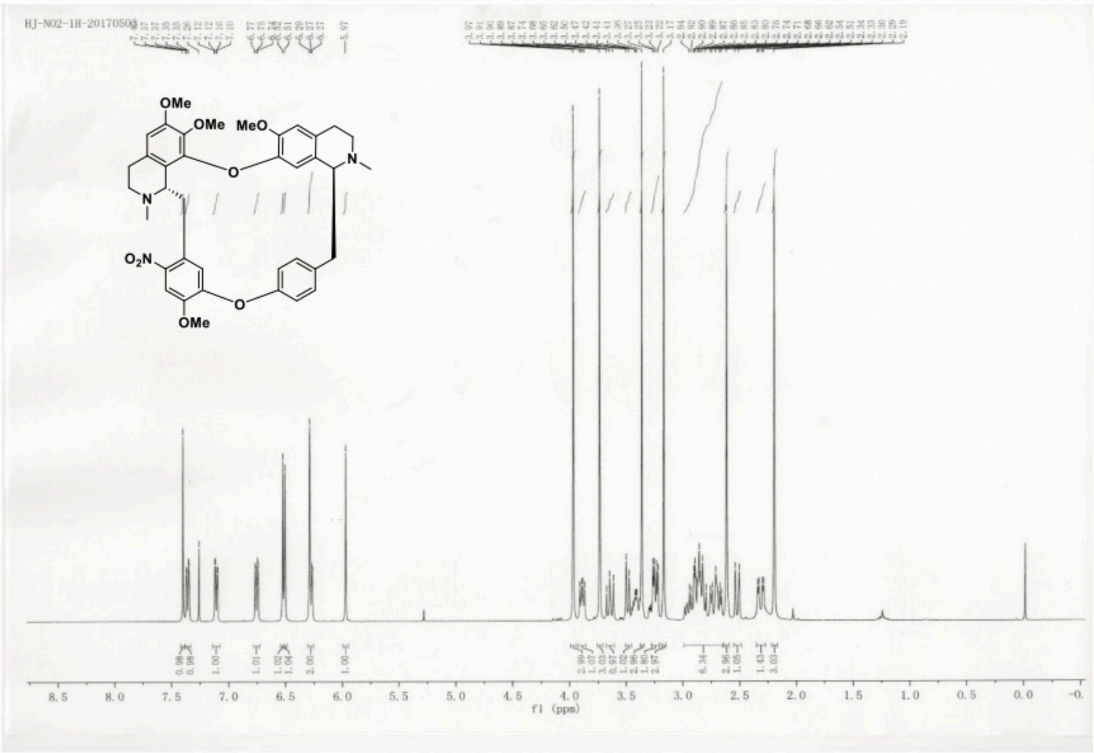

Figure S1. ^{1}H-NMR Spectra of **Tet-NO$_2$** in CDCl$_3$.

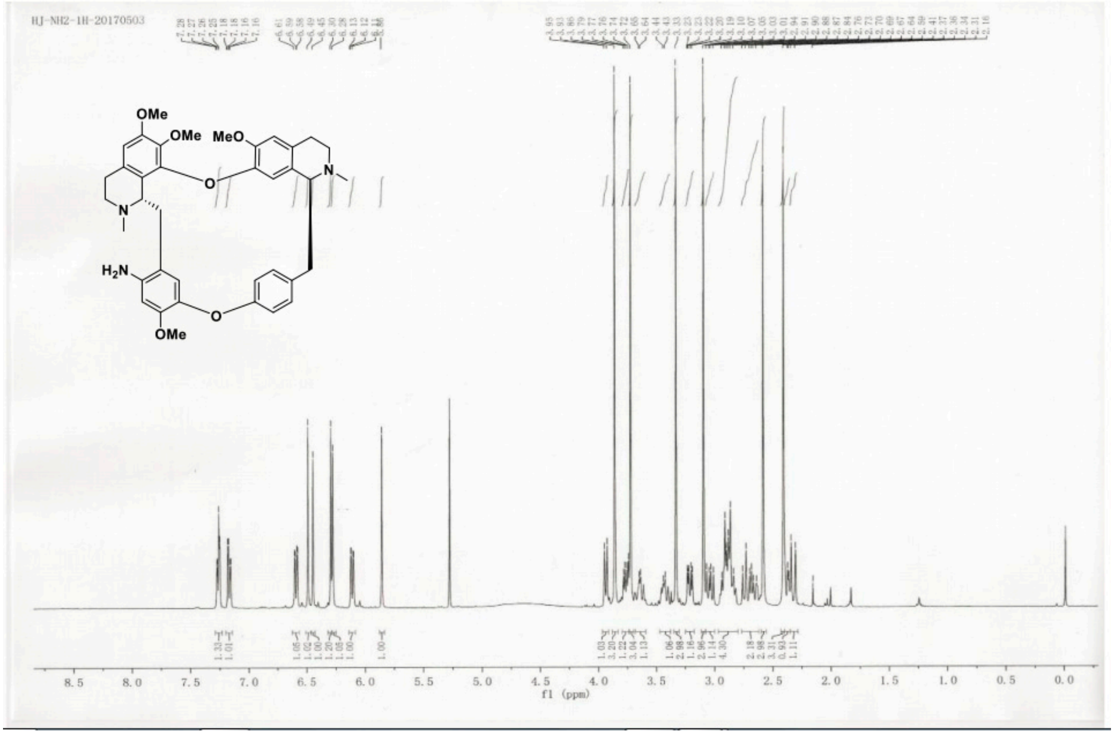

Figure S2. ^{1}H-NMR Spectra of **Tet-NH$_2$** in CDCl$_3$.

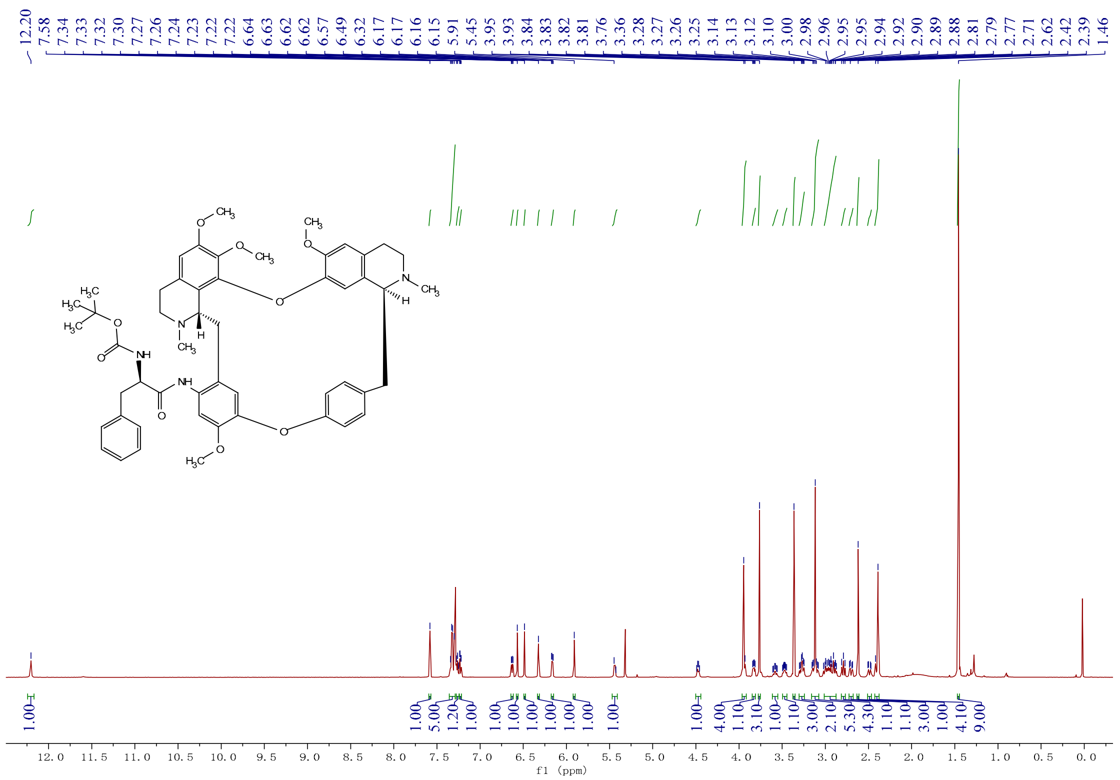

Figure S3. ^{1}H-NMR Spectra of **1a** in $CDCl_3$.

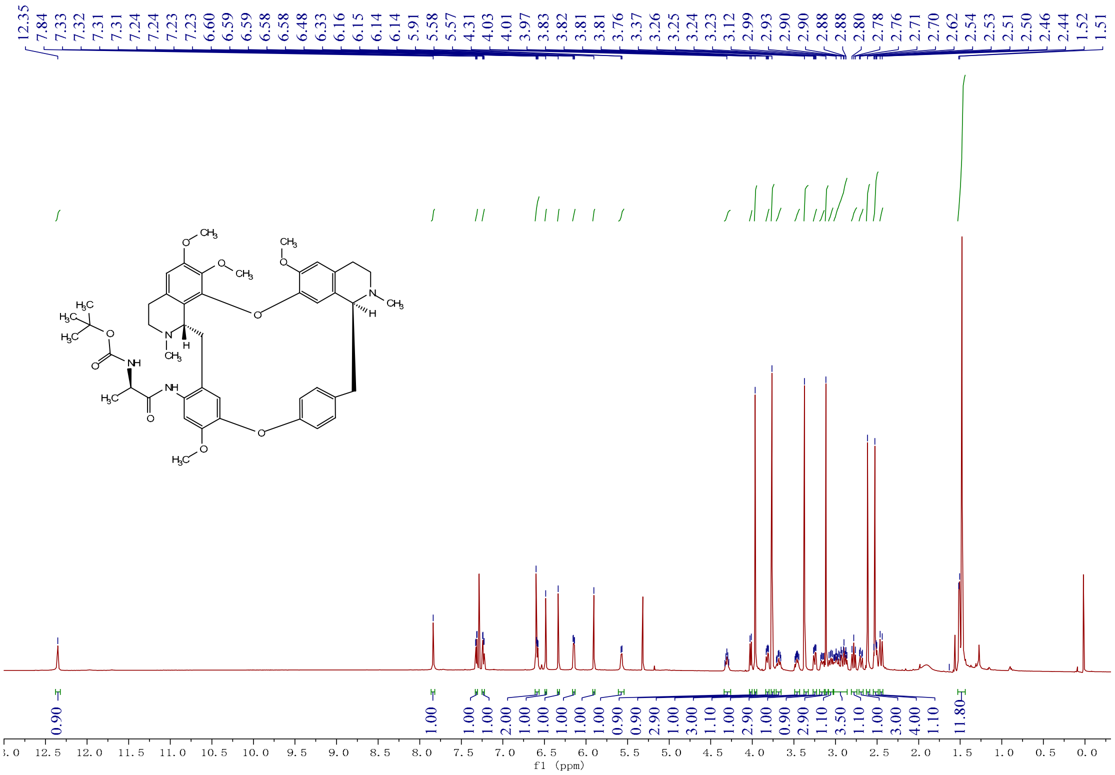

Figure S4. ^{1}H-NMR Spectra of **1b** in $CDCl_3$.

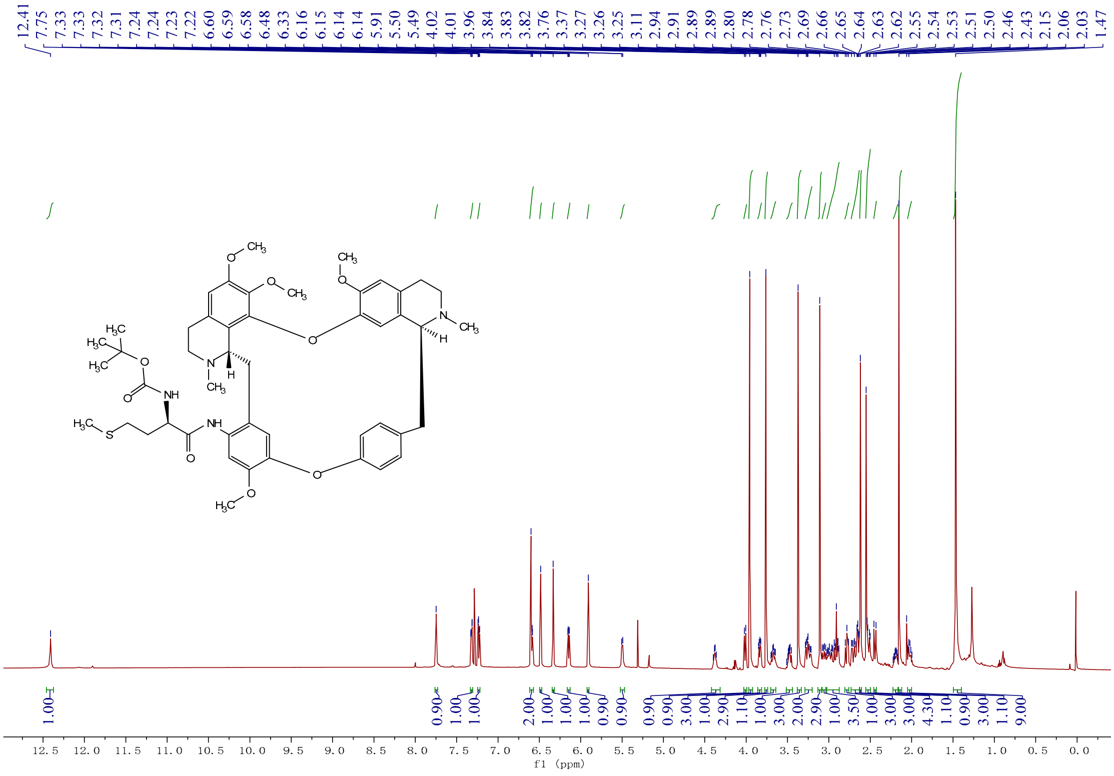

Figure S5. ^{1}H-NMR Spectra of **1c** in $CDCl_3$.

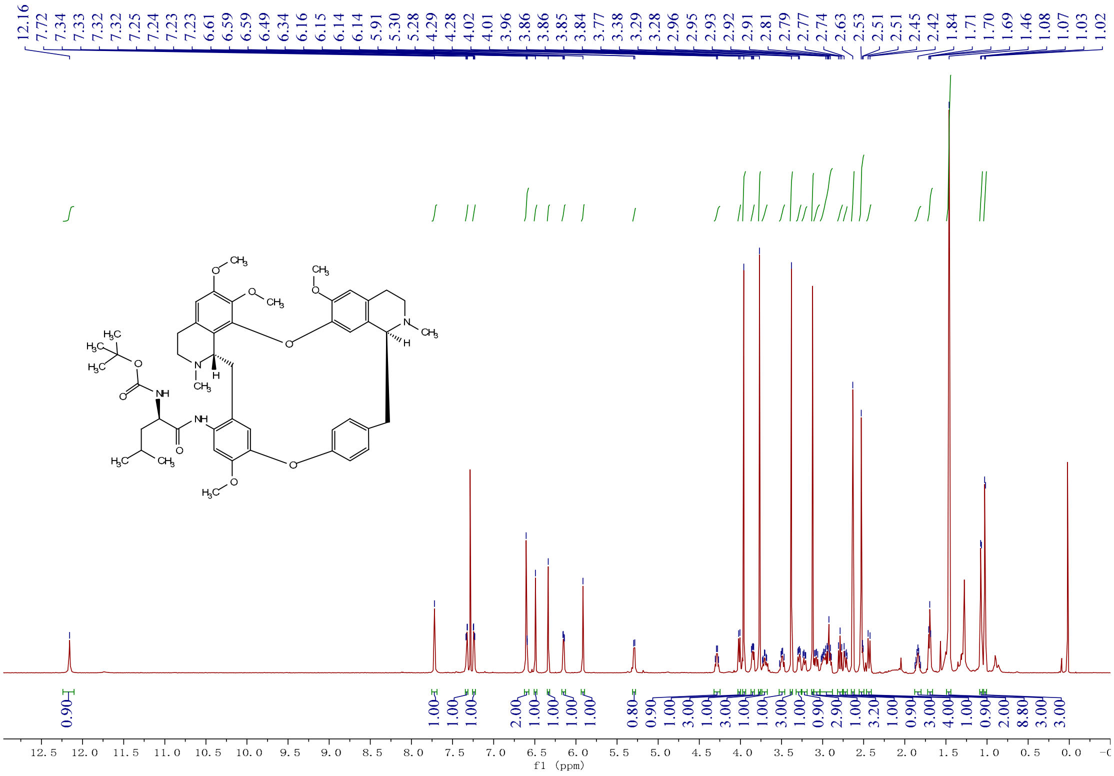

Figure S6. ^{1}H-NMR Spectra of **1d** in $CDCl_3$.

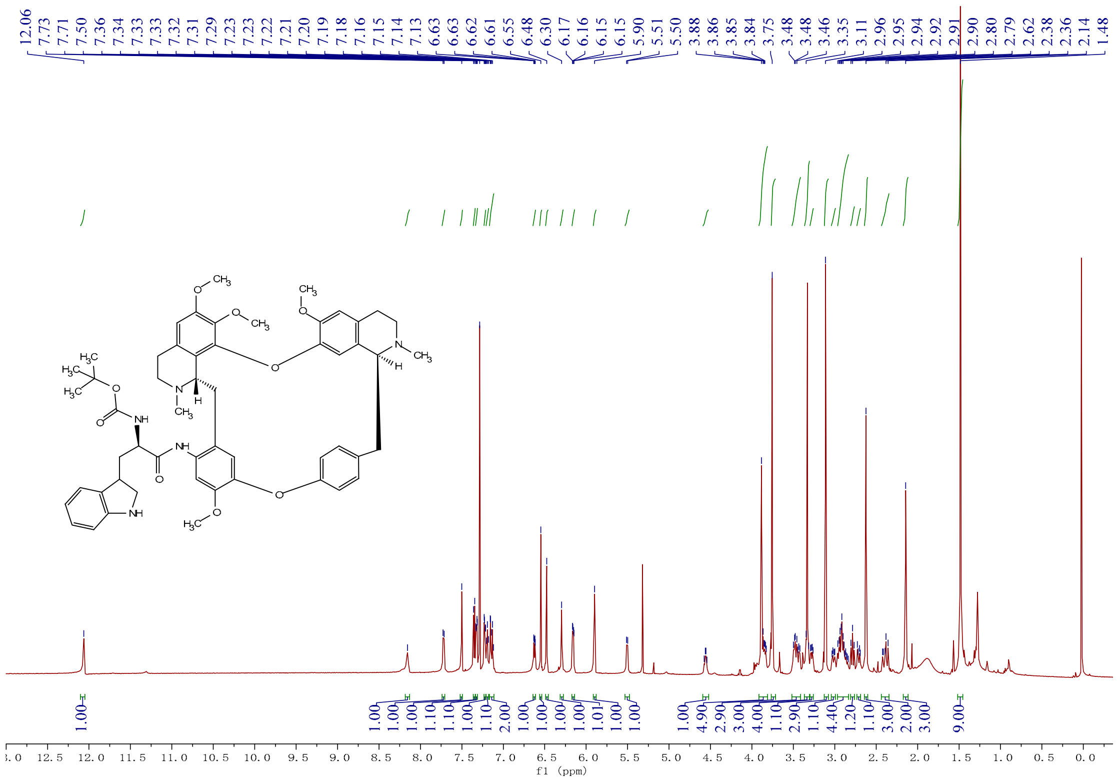

Figure S7. ^{1}H-NMR Spectra of **1e** in $CDCl_3$.

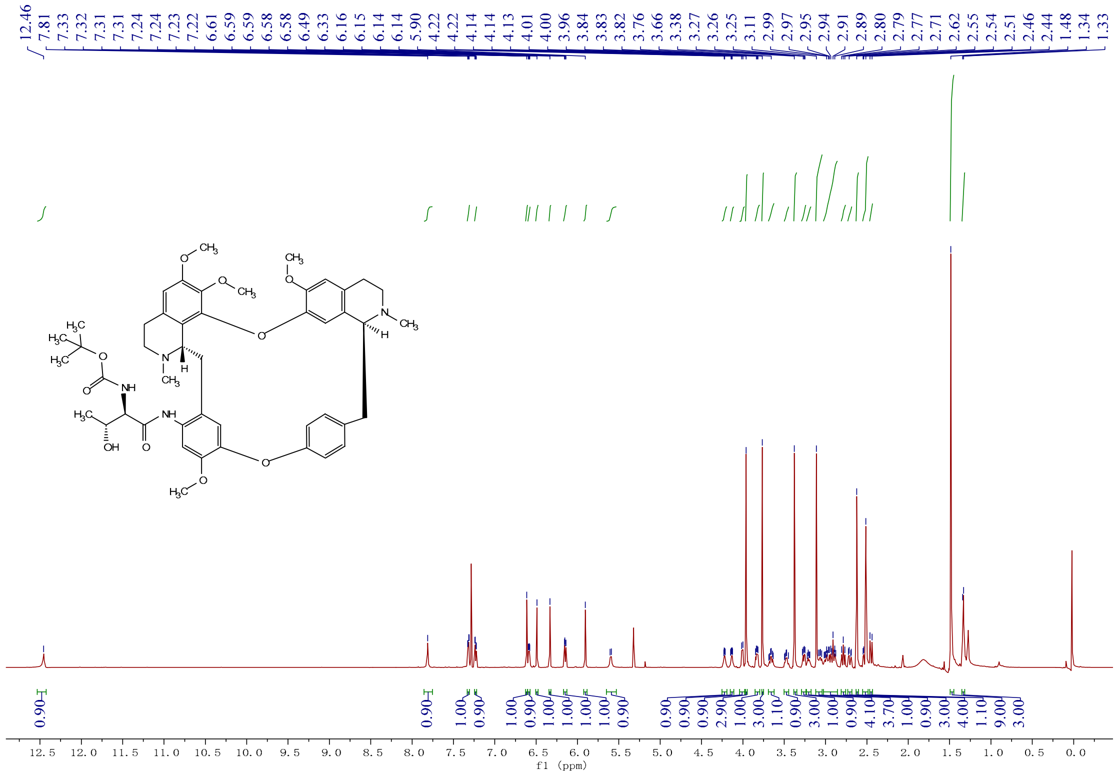

Figure S8. ^{1}H-NMR Spectra of **1f** in $CDCl_3$.

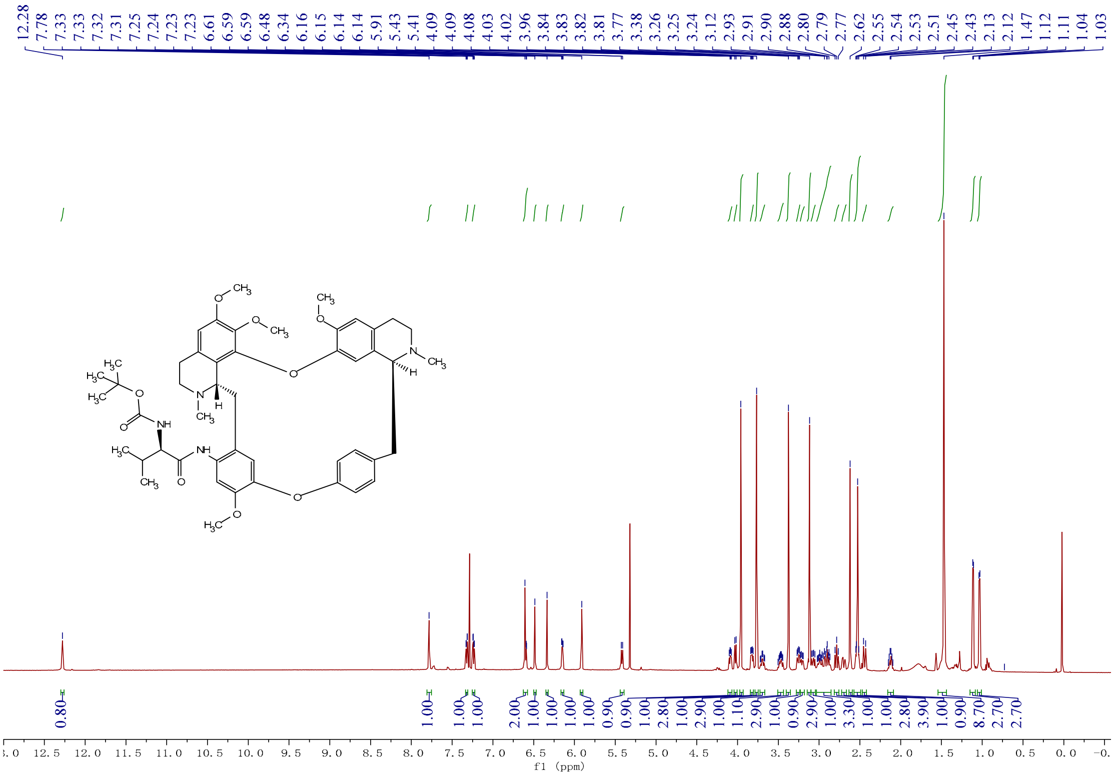

Figure S9. ^{1}H-NMR Spectra of **1g** in $CDCl_3$.

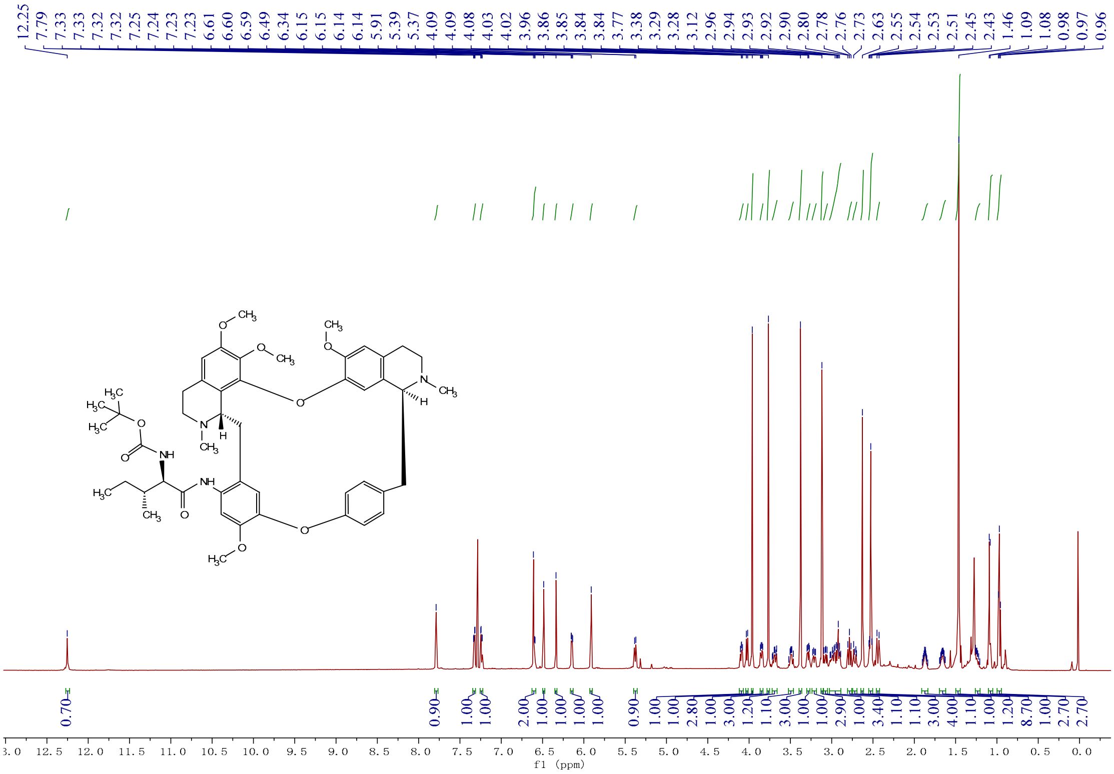

Figure S10. ^{1}H-NMR Spectra of **1h** in $CDCl_3$.

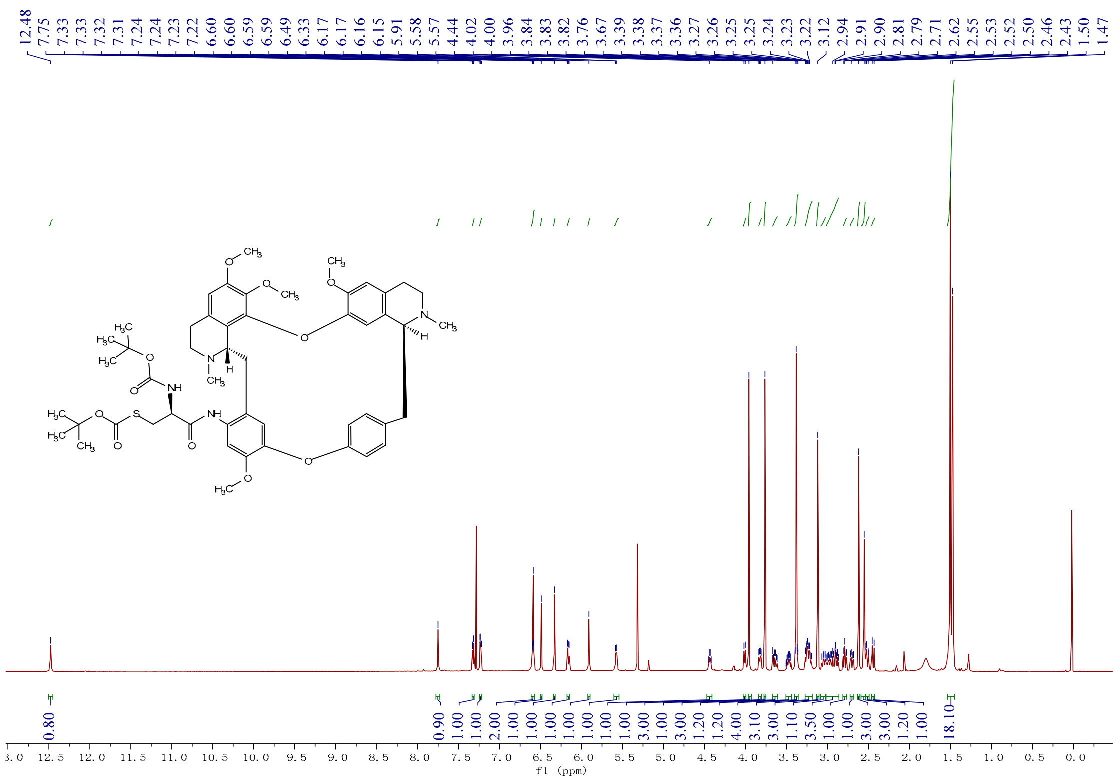

Figure S11. ^{1}H-NMR Spectra of **1i** in $CDCl_3$.

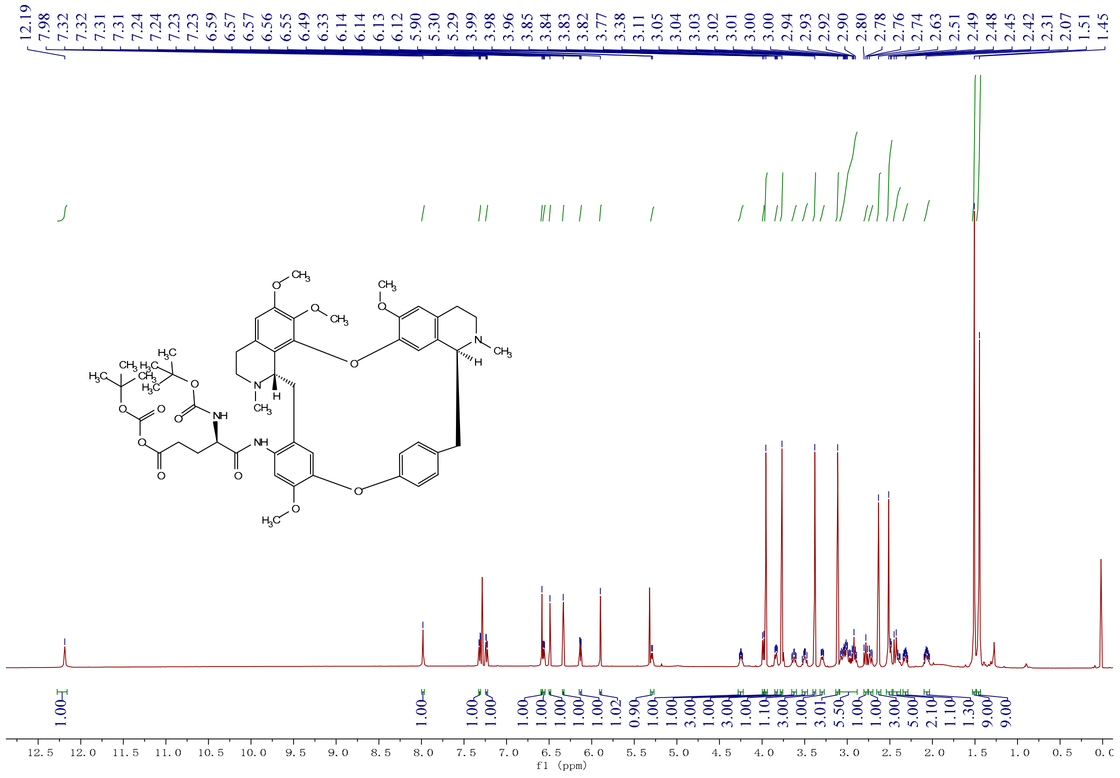

Figure S12. ^{1}H-NMR Spectra of **1j** in $CDCl_3$.

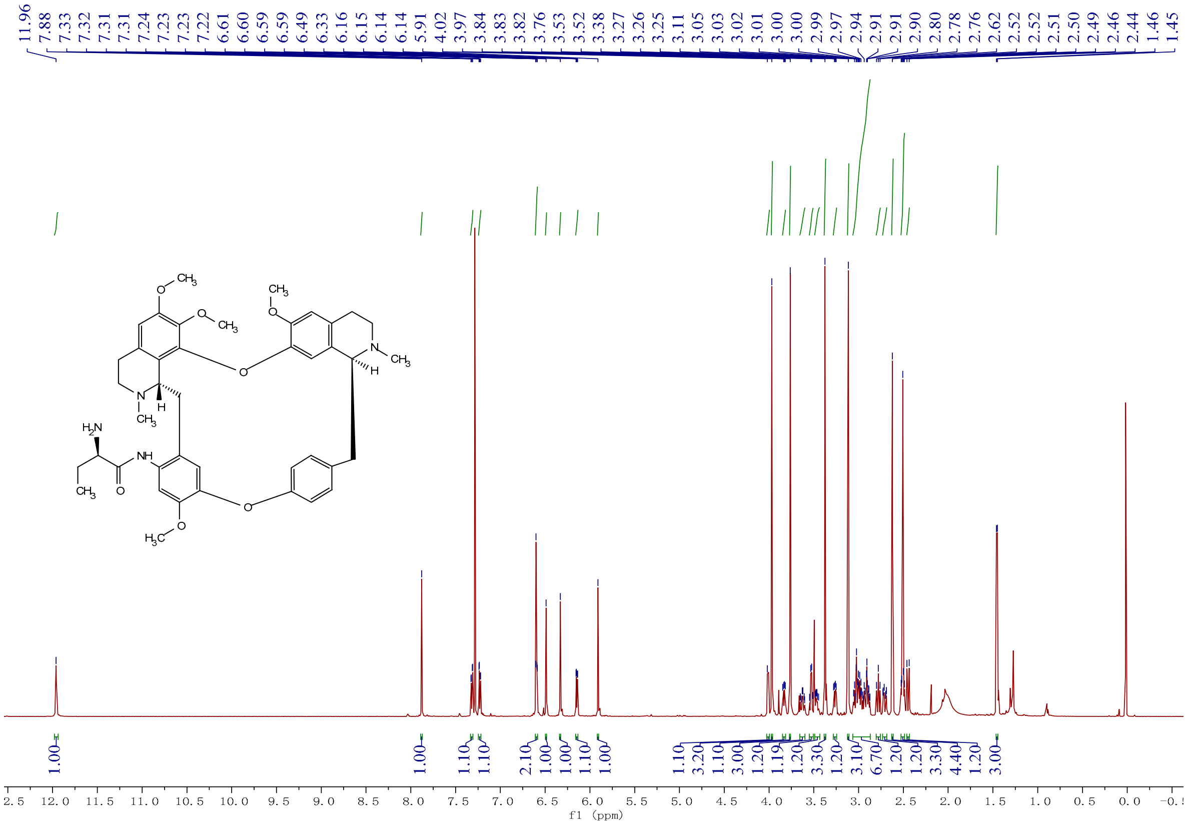

Figure S13. ^{1}H-NMR Spectra of **1k** in $CDCl_3$.

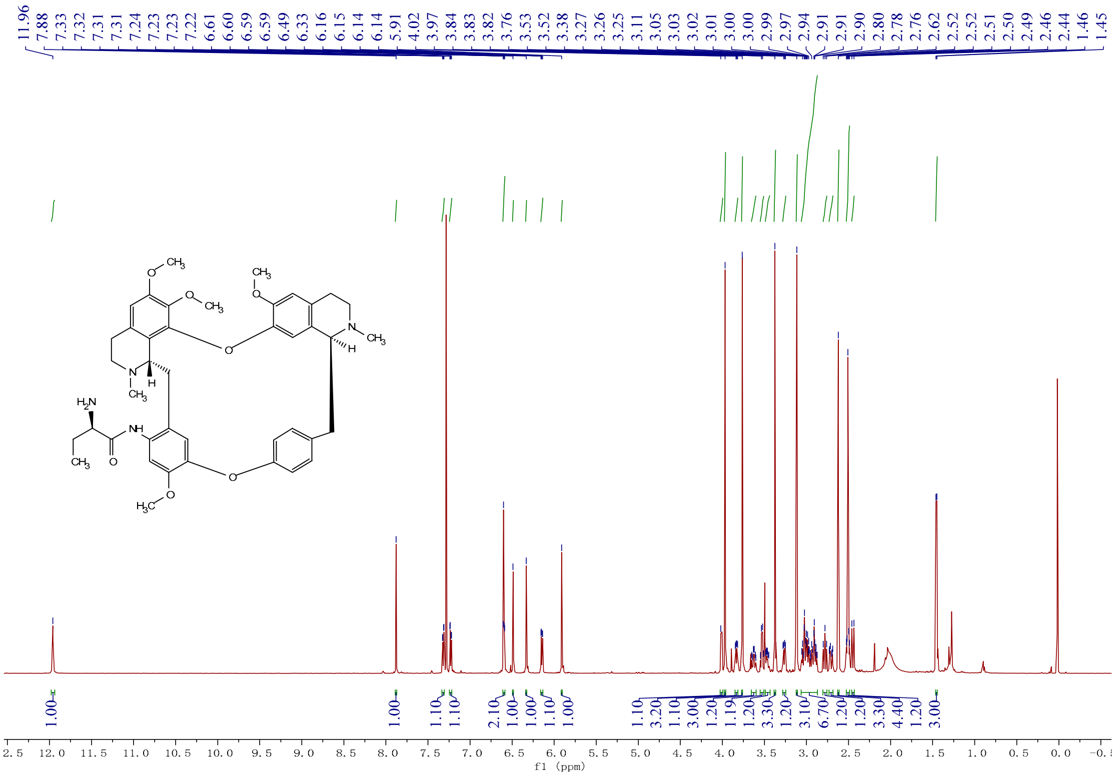

Figure S14. ^{1}H-NMR Spectra of **1l** in $CDCl_3$.

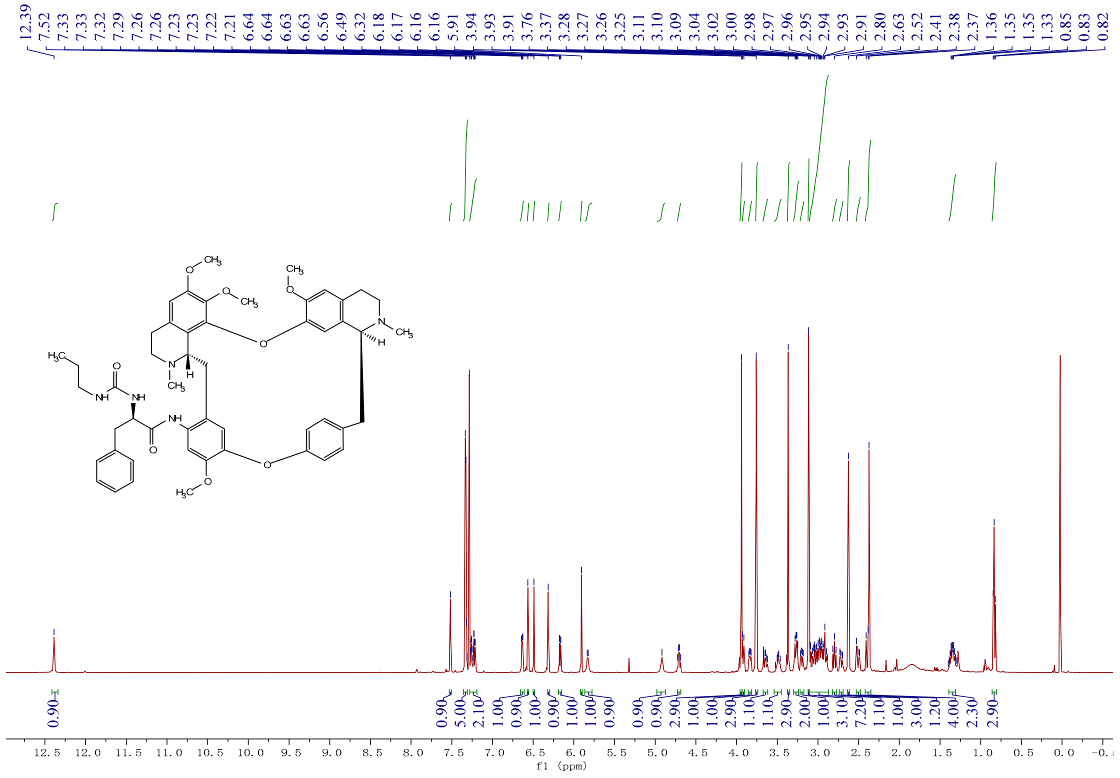

Figure S15. ^{1}H-NMR Spectra of **2a** in $CDCl_3$.

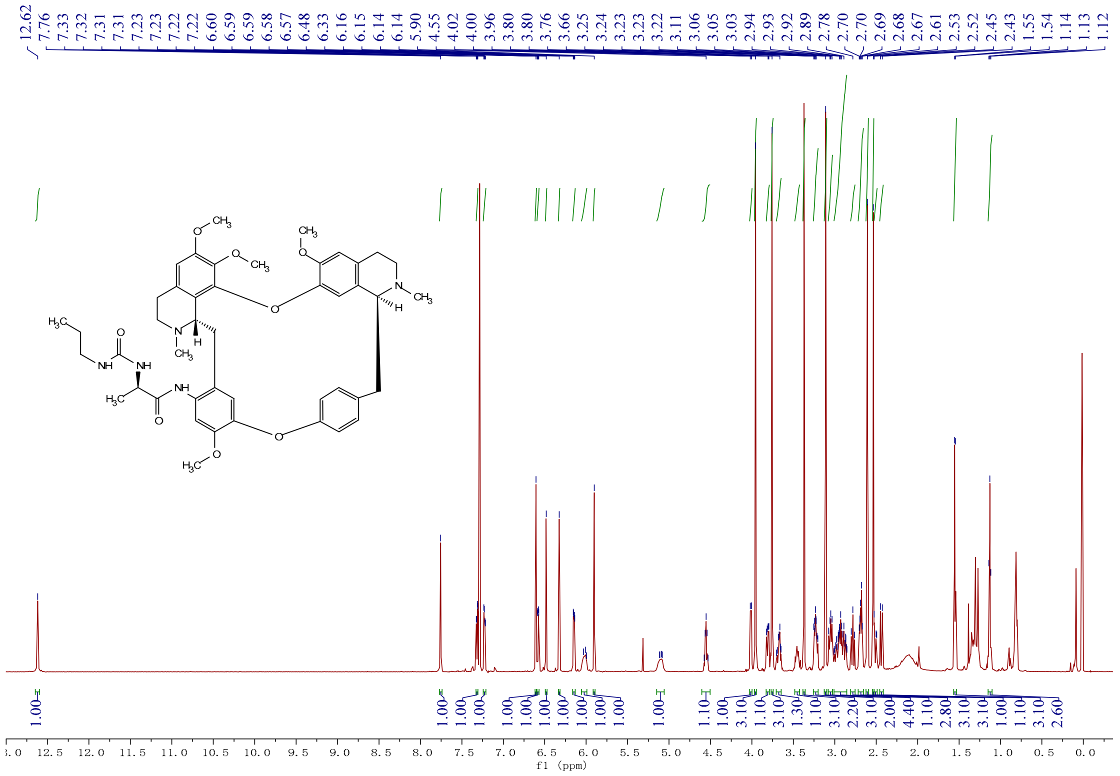

Figure S16. ^{1}H-NMR Spectra of **3a** in $CDCl_3$.

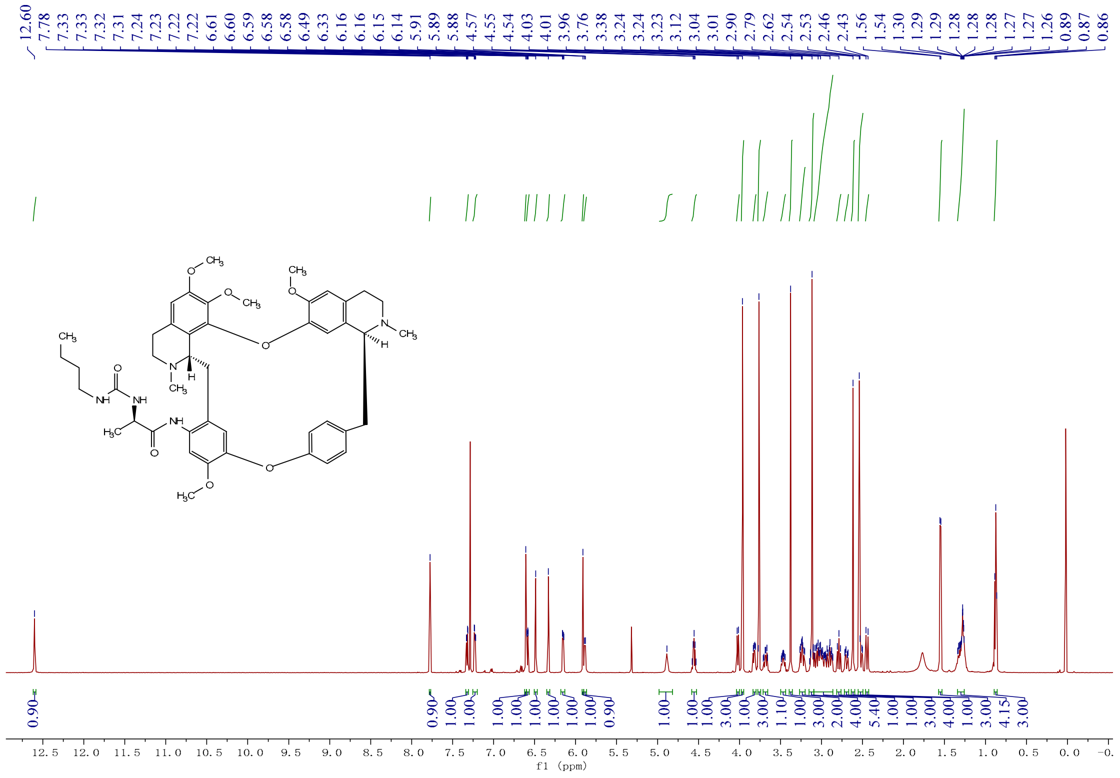

Figure S17. ^{1}H-NMR Spectra of **3b** in $CDCl_3$.

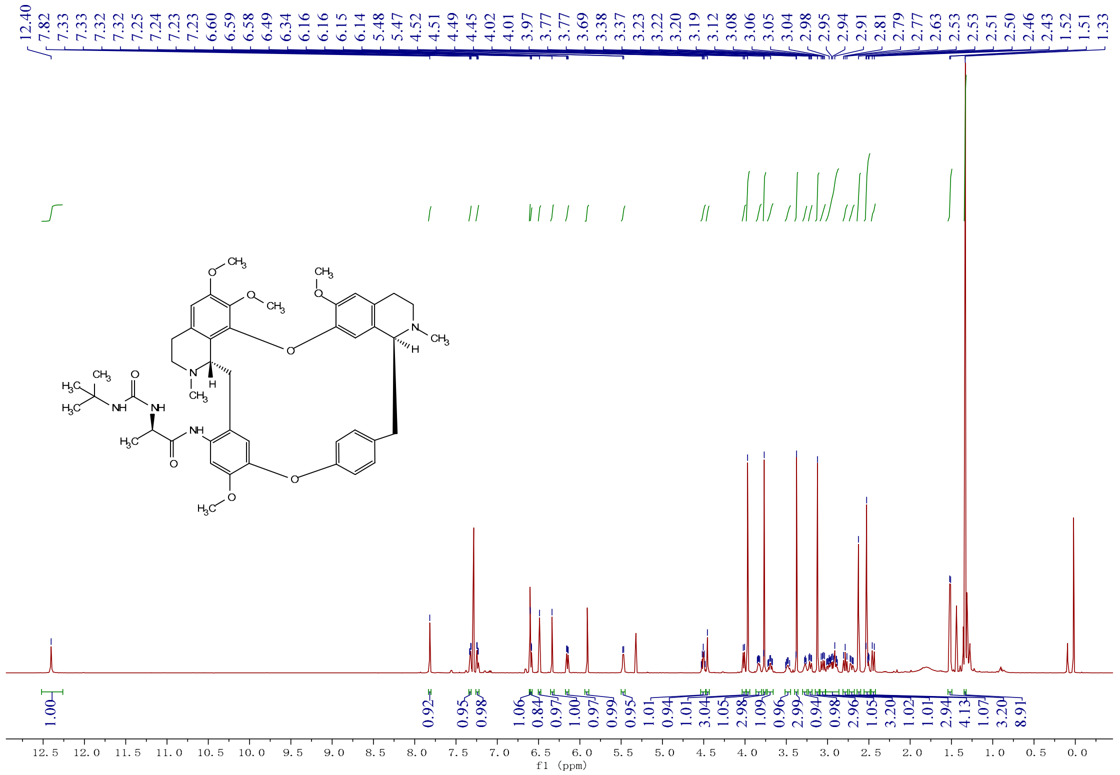

Figure S18. ^{1}H-NMR Spectra of **3c** in $CDCl_3$.

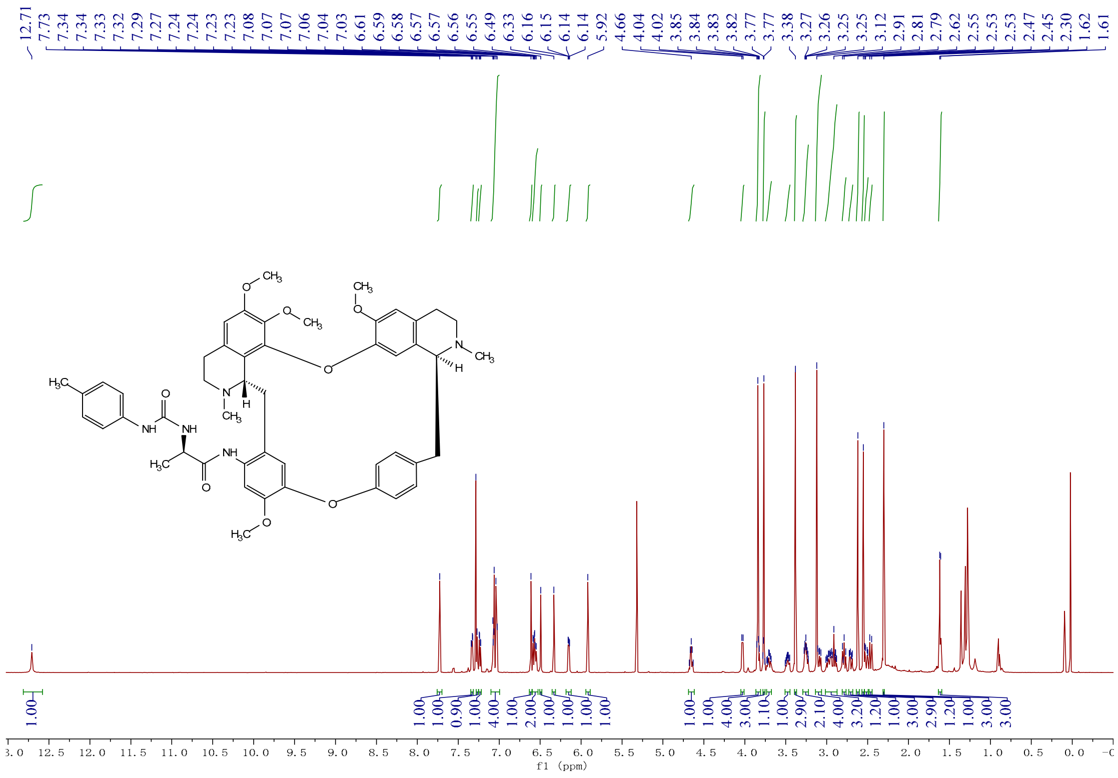

Figure S19. ^{1}H-NMR Spectra of **3d** in $CDCl_3$.

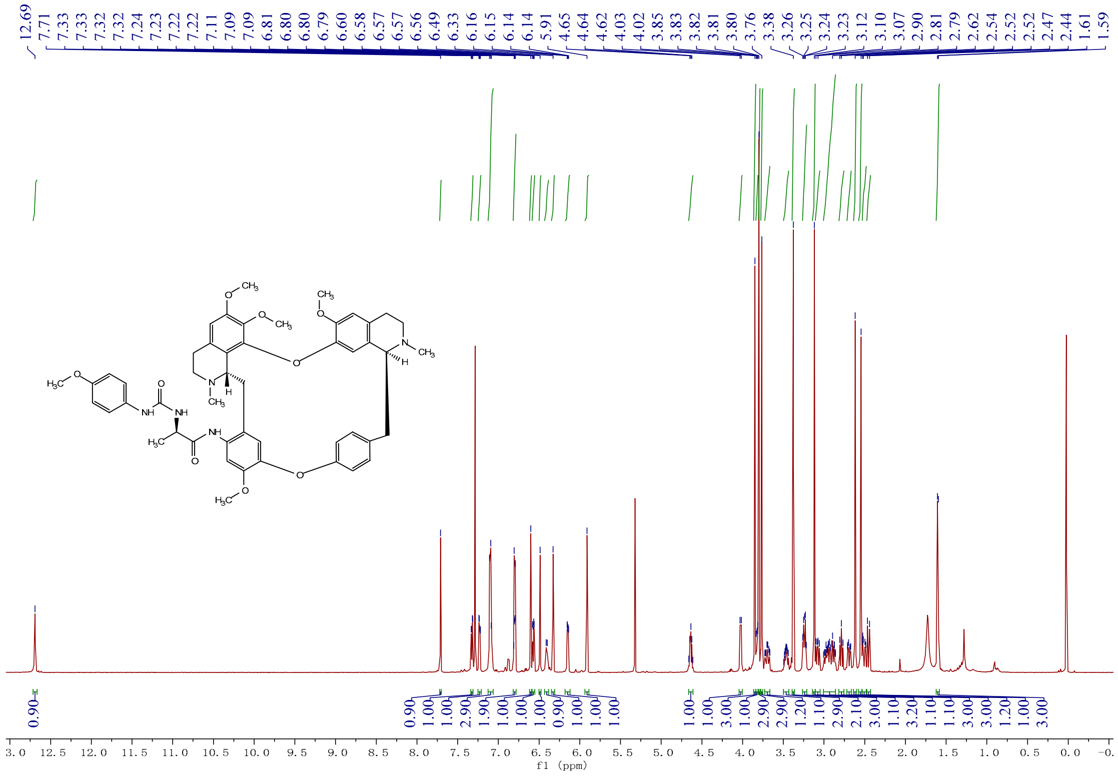

Figure S20. ^{1}H-NMR Spectra of **3e** in $CDCl_3$.

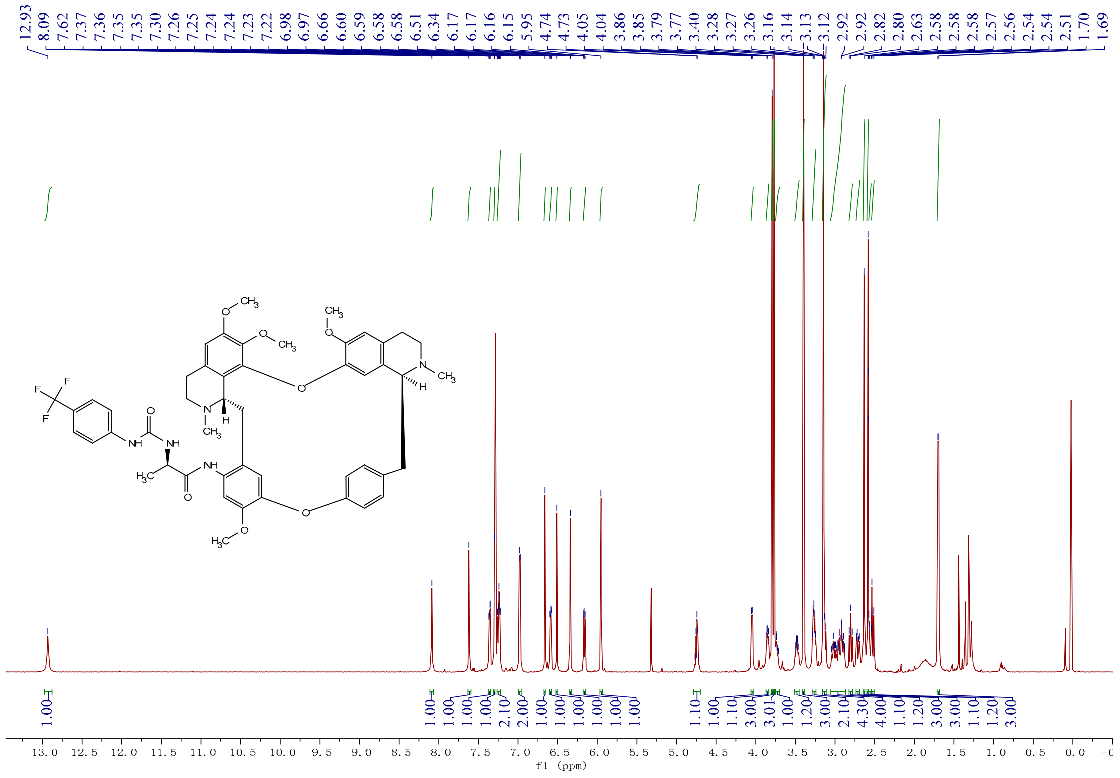

Figure S21. ^{1}H-NMR Spectra of **3f** in $CDCl_3$.

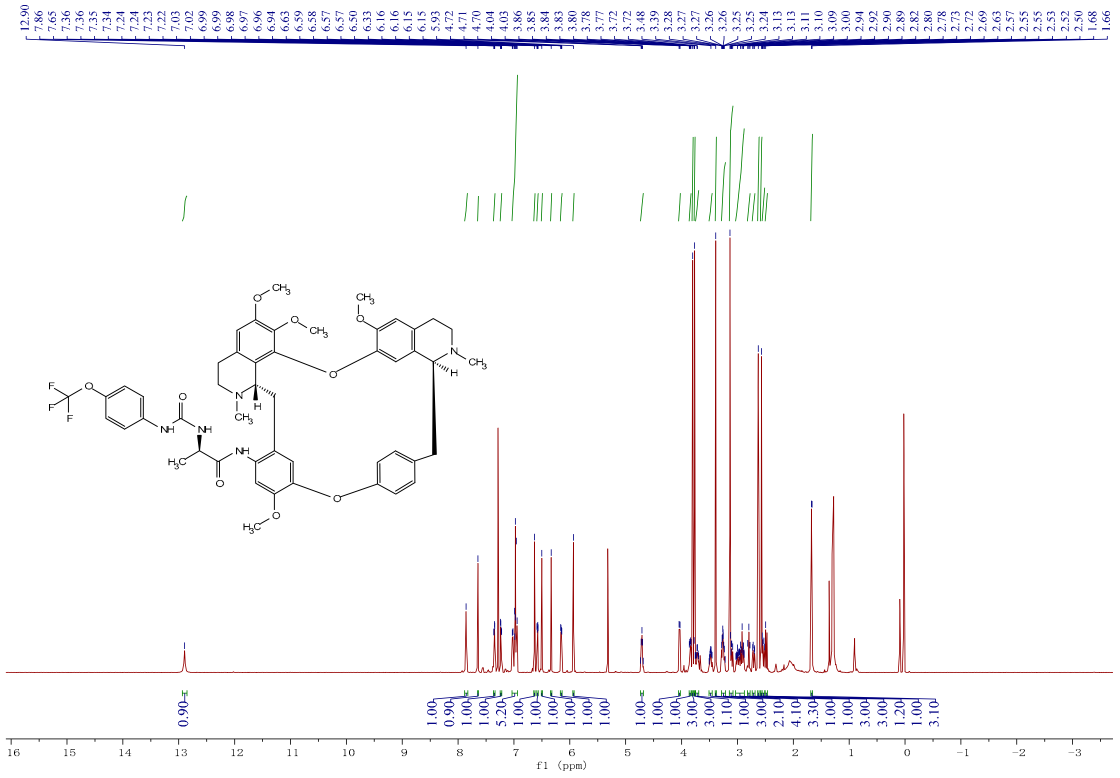

Figure S22. ^{1}H-NMR Spectra of **3g** in $CDCl_3$.

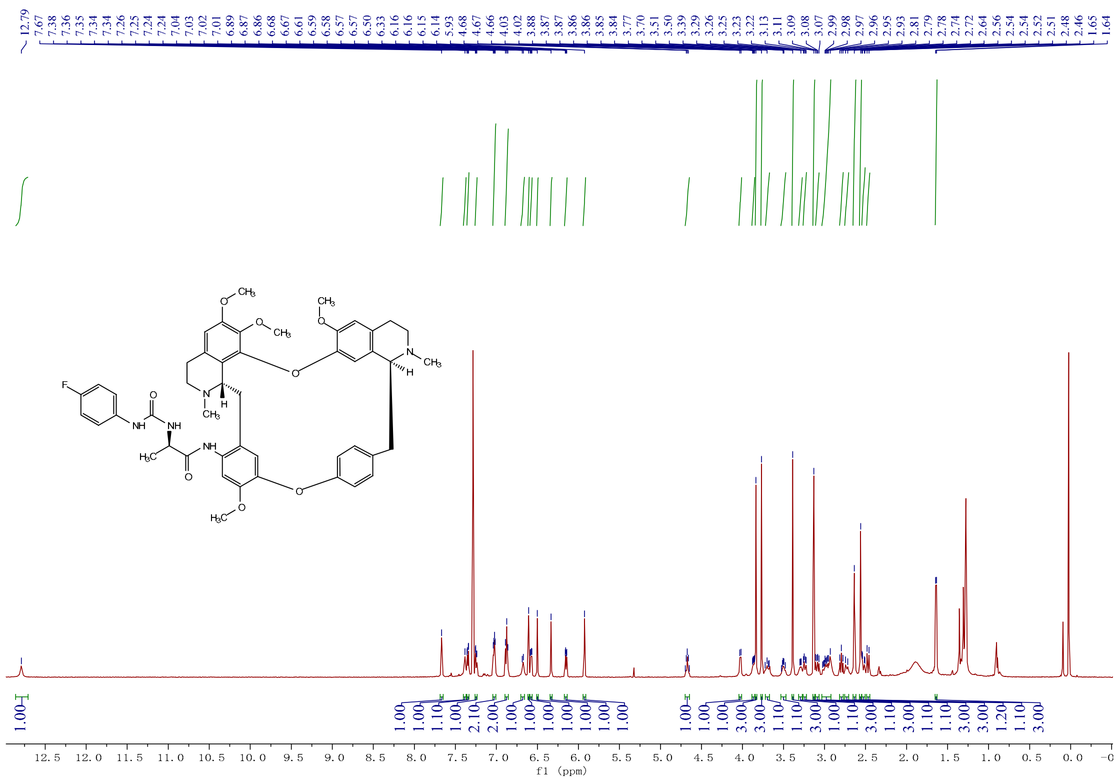

Figure S23. ^{1}H-NMR Spectra of **3h** in $CDCl_3$.

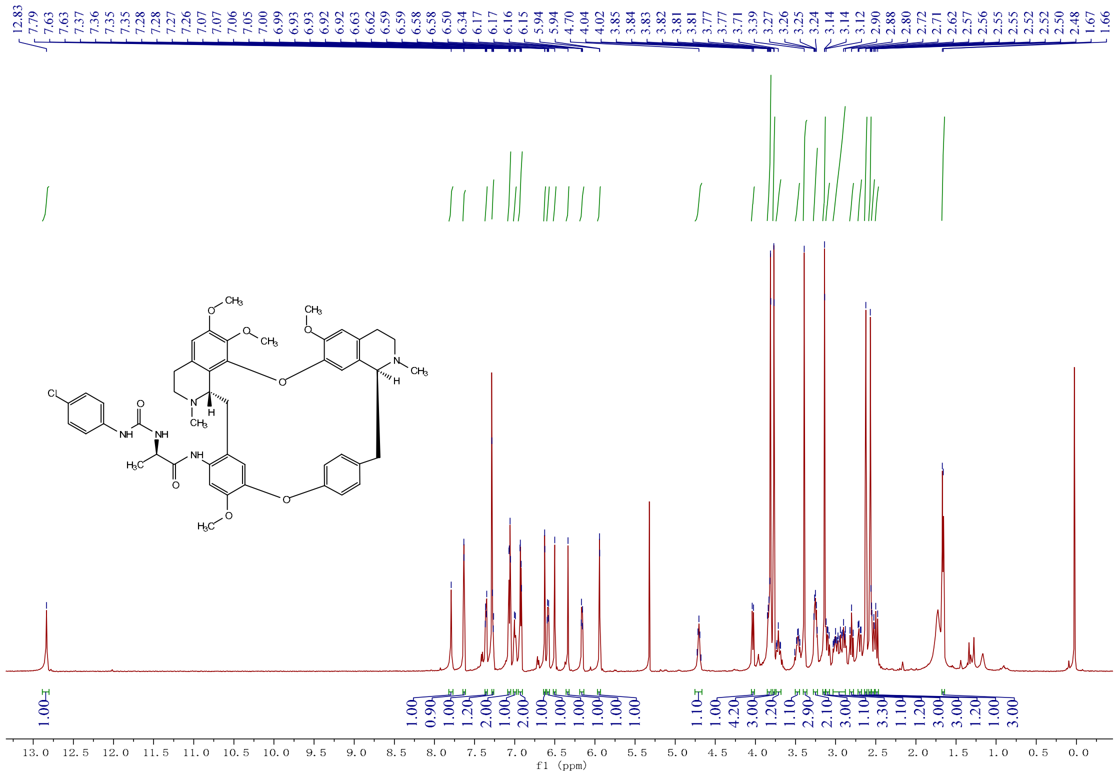

Figure S24. ^{1}H-NMR Spectra of **3i** in $CDCl_3$.

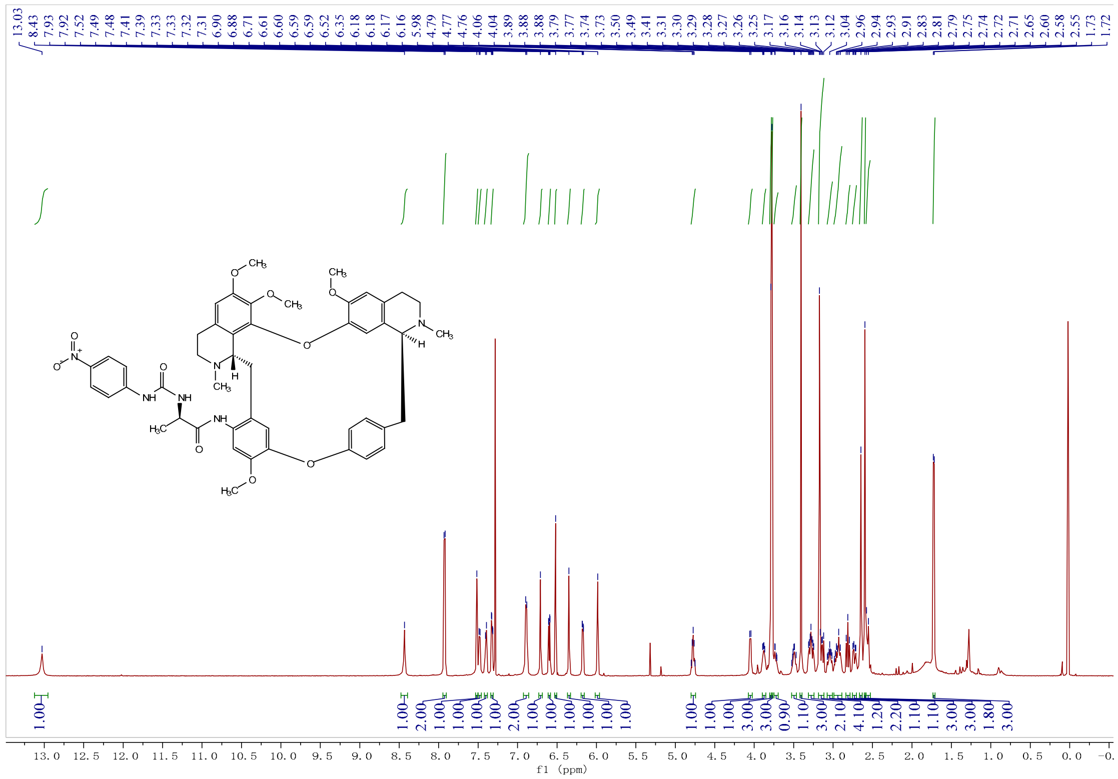

Figure S25. ^{1}H-NMR Spectra of **3j** in $CDCl_3$.

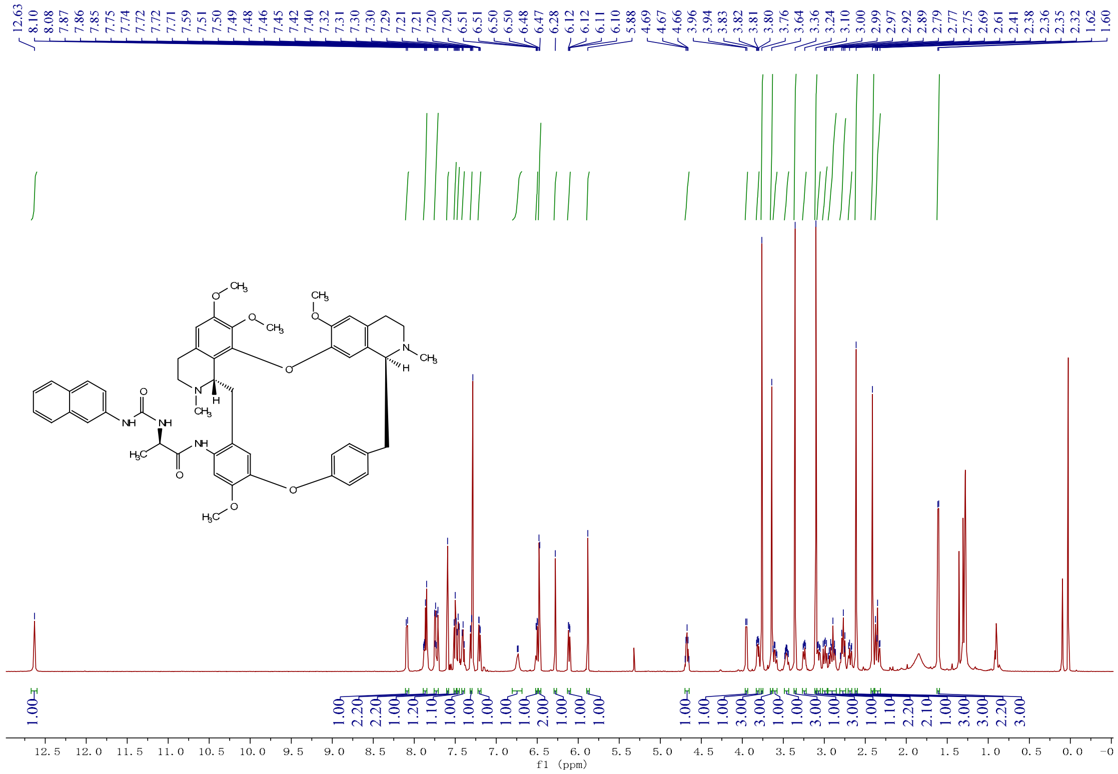

Figure S26. ^{1}H-NMR Spectra of **3k** in $CDCl_3$.

2. ^{13}C-NMR Spectra of 1a-3k

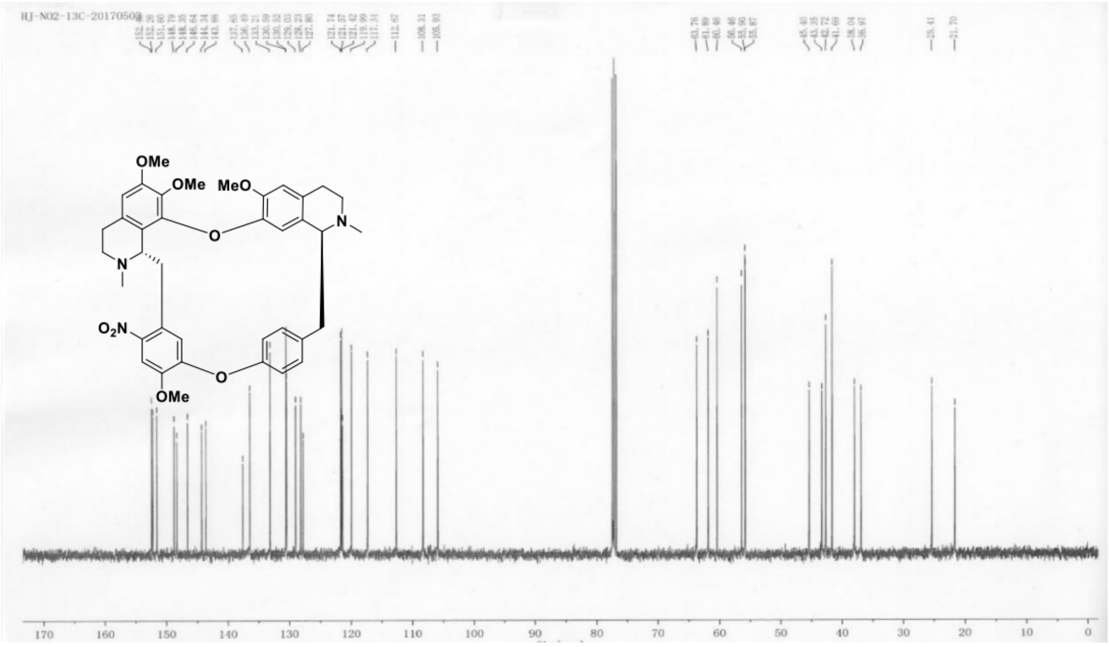

Figure S27. ^{13}C-NMR Spectra of **Tet-NO_2** in $CDCl_3$.

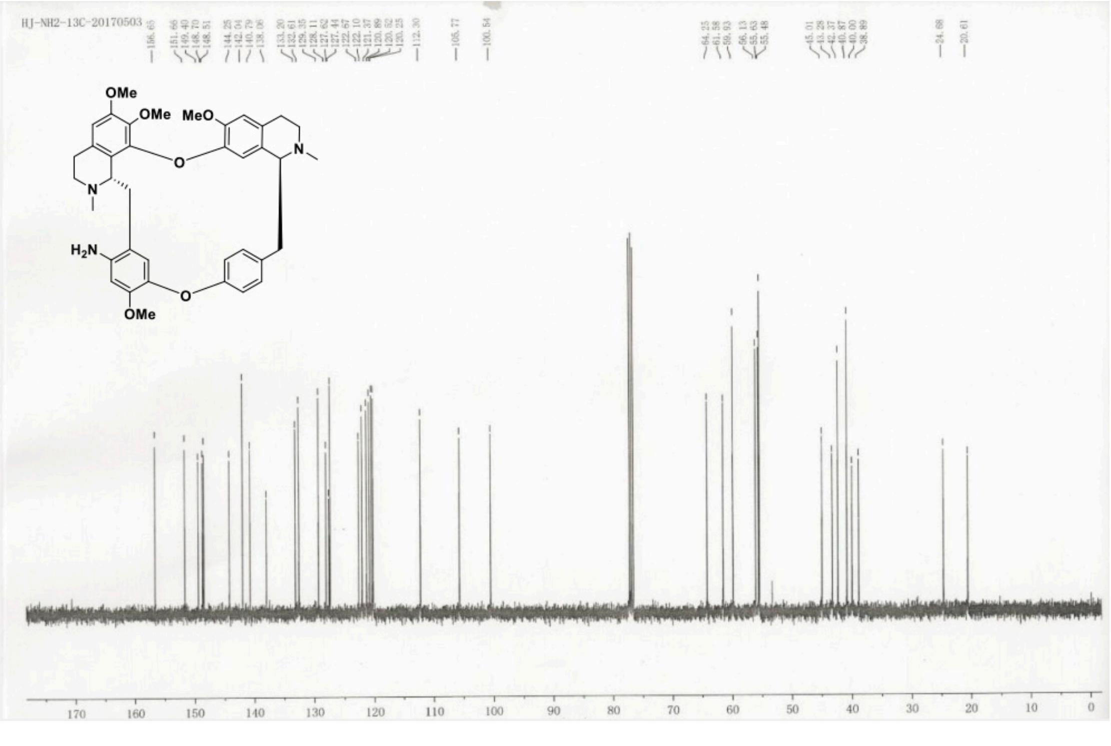

Figure S28. ^{13}C-NMR Spectra of **Tet-NH_2** in $CDCl_3$.

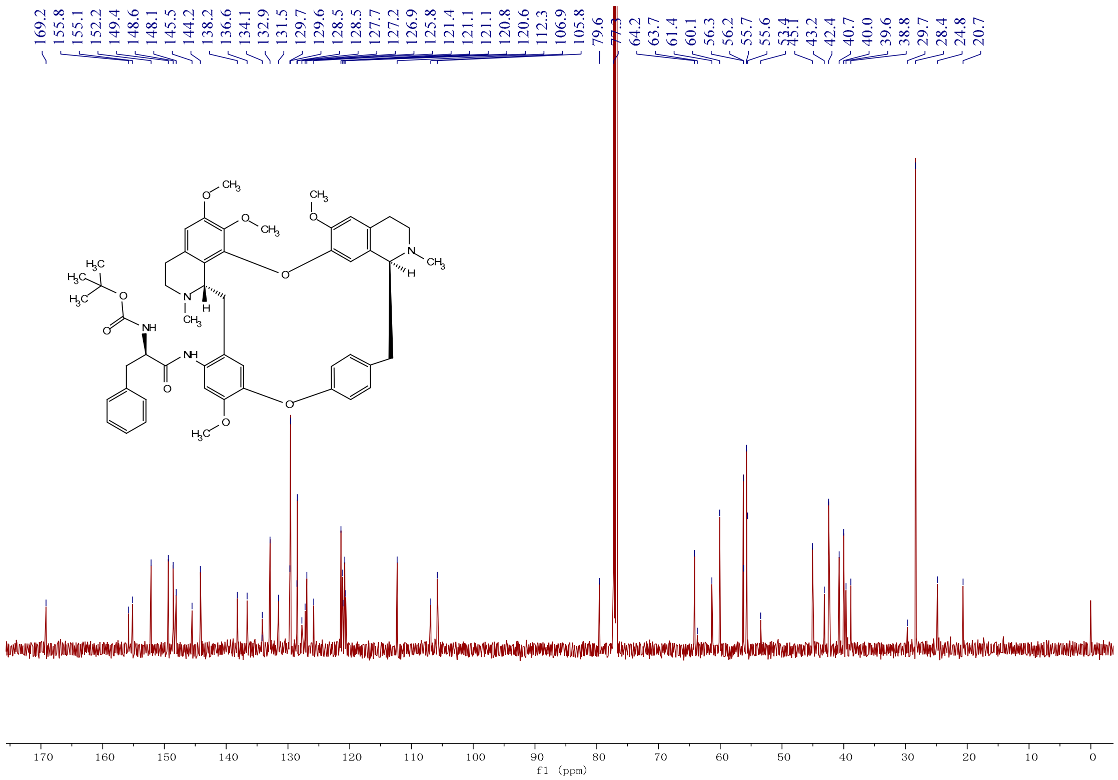

Figure S29. ^{13}C-NMR Spectra of **1a** in $CDCl_3$.

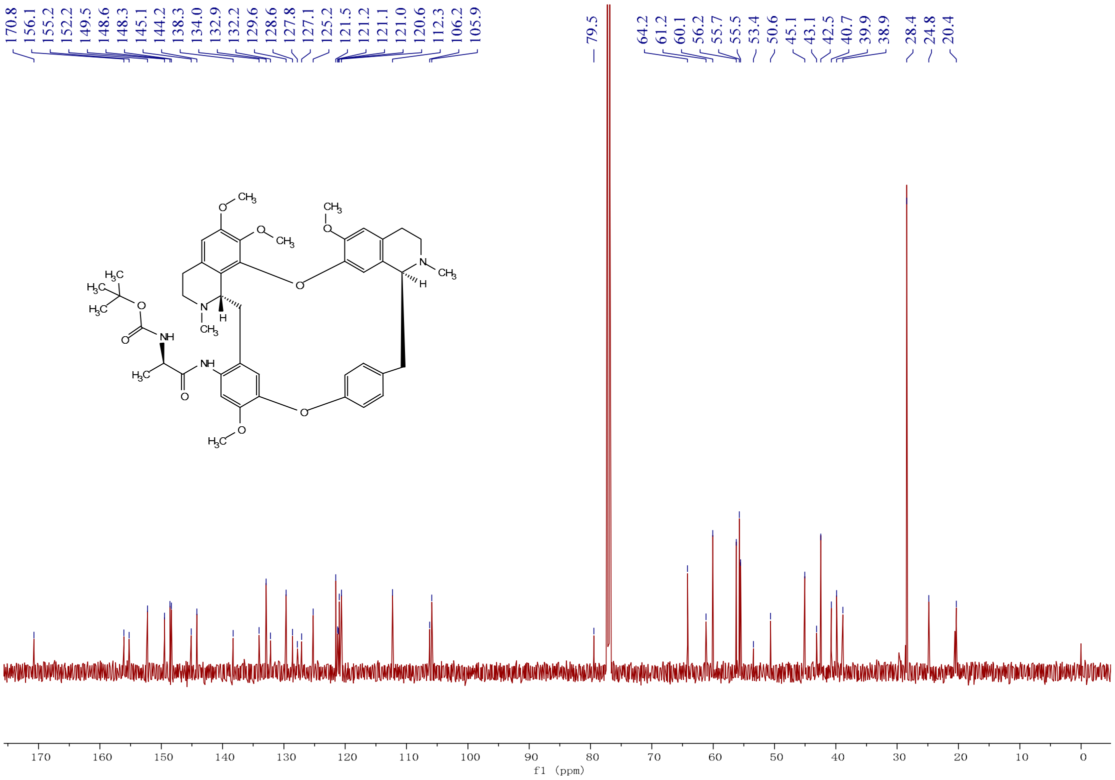

Figure S30. ^{13}C-NMR Spectra of **1b** in $CDCl_3$.

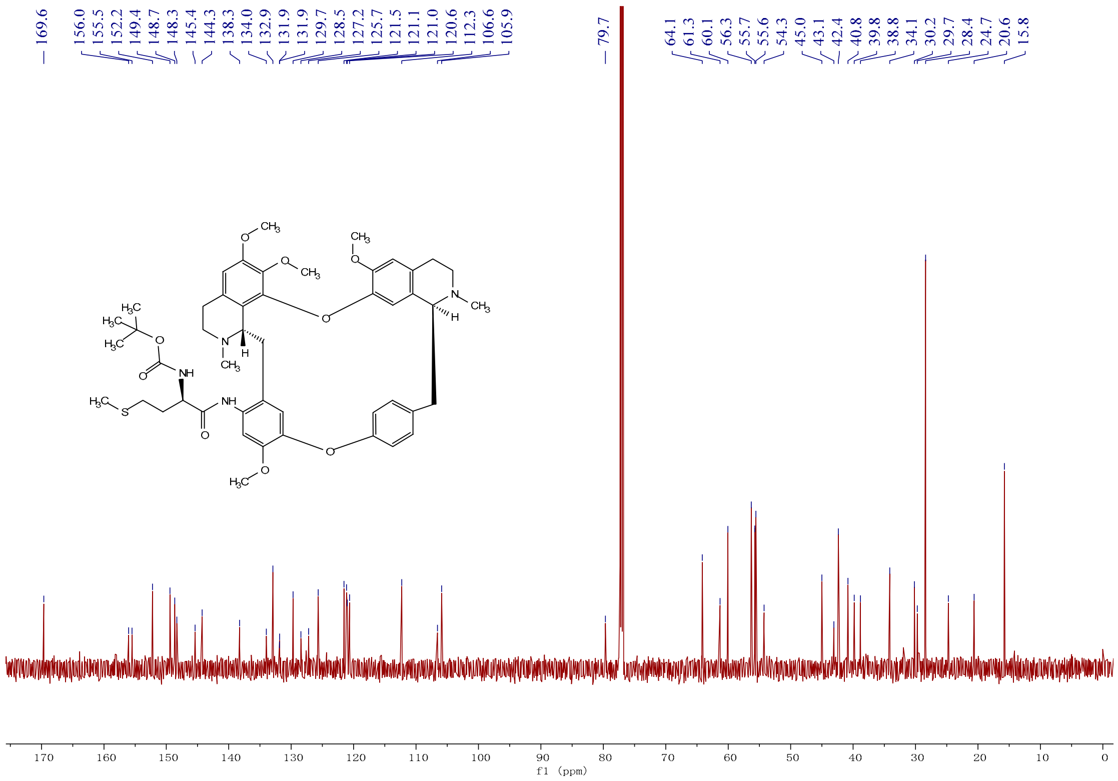

Figure S31. ^{13}C-NMR Spectra of **1c** in $CDCl_3$.

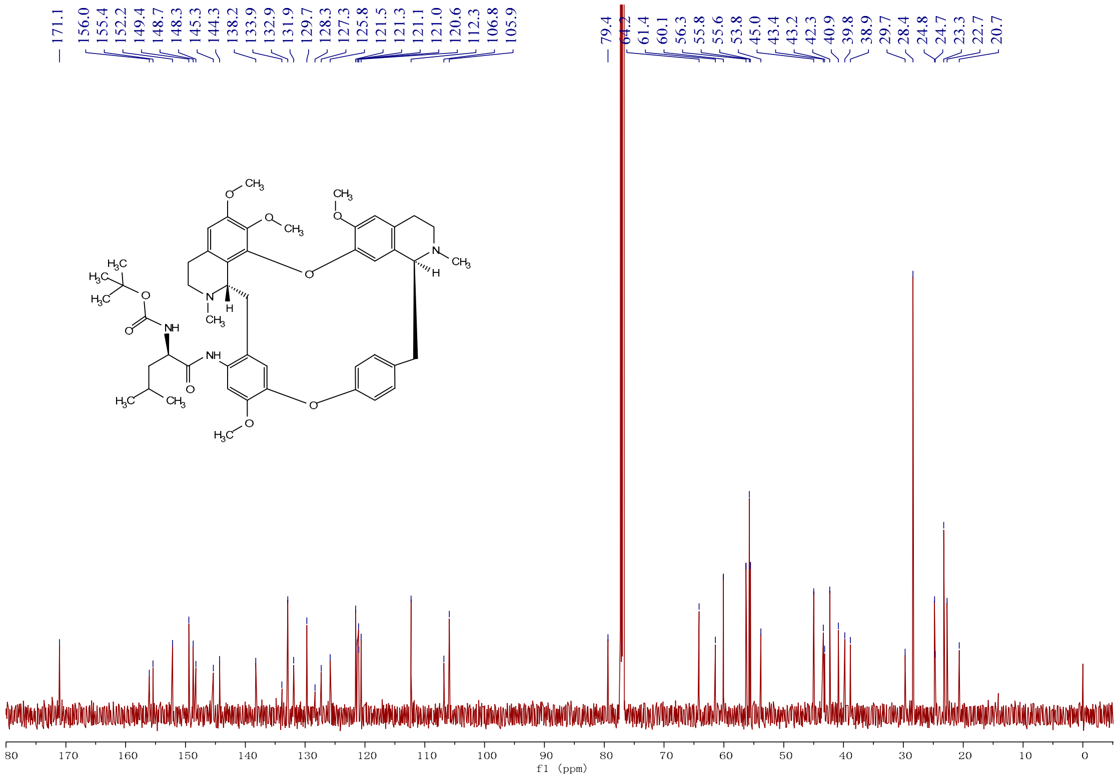

Figure S32. ^{13}C-NMR Spectra of **1d** in $CDCl_3$.

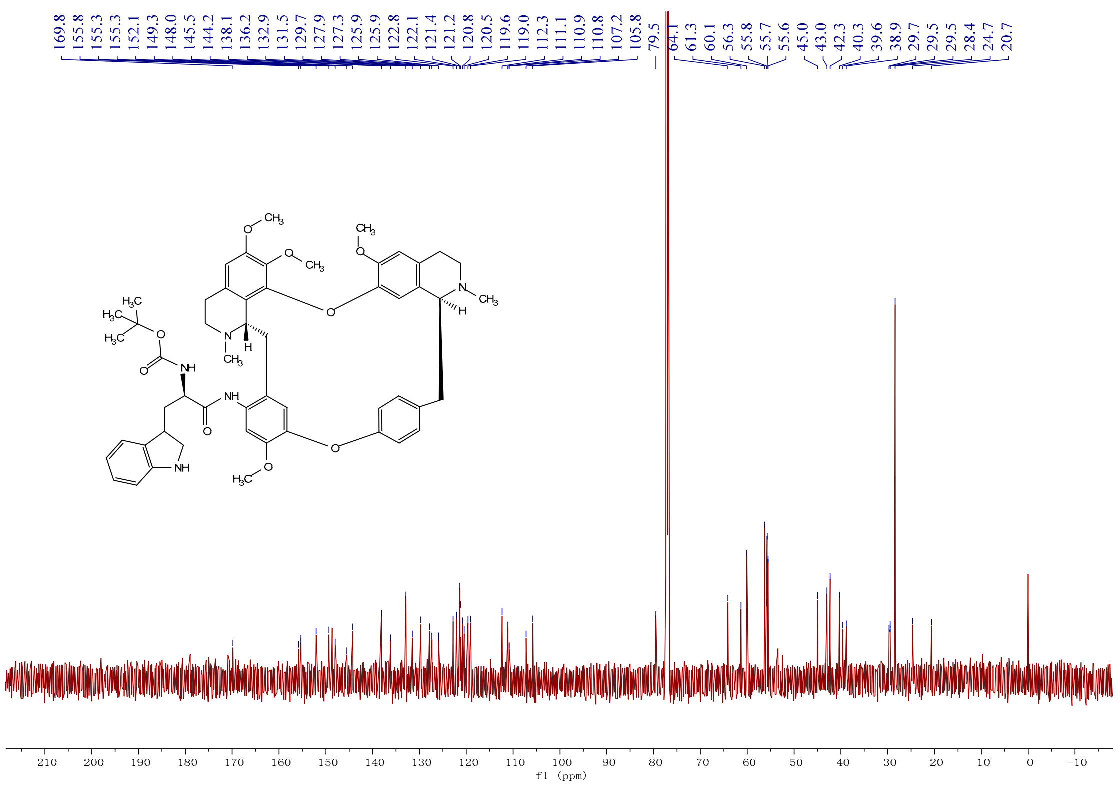

Figure S33. ^{13}C-NMR Spectra of **1e** in $CDCl_3$.

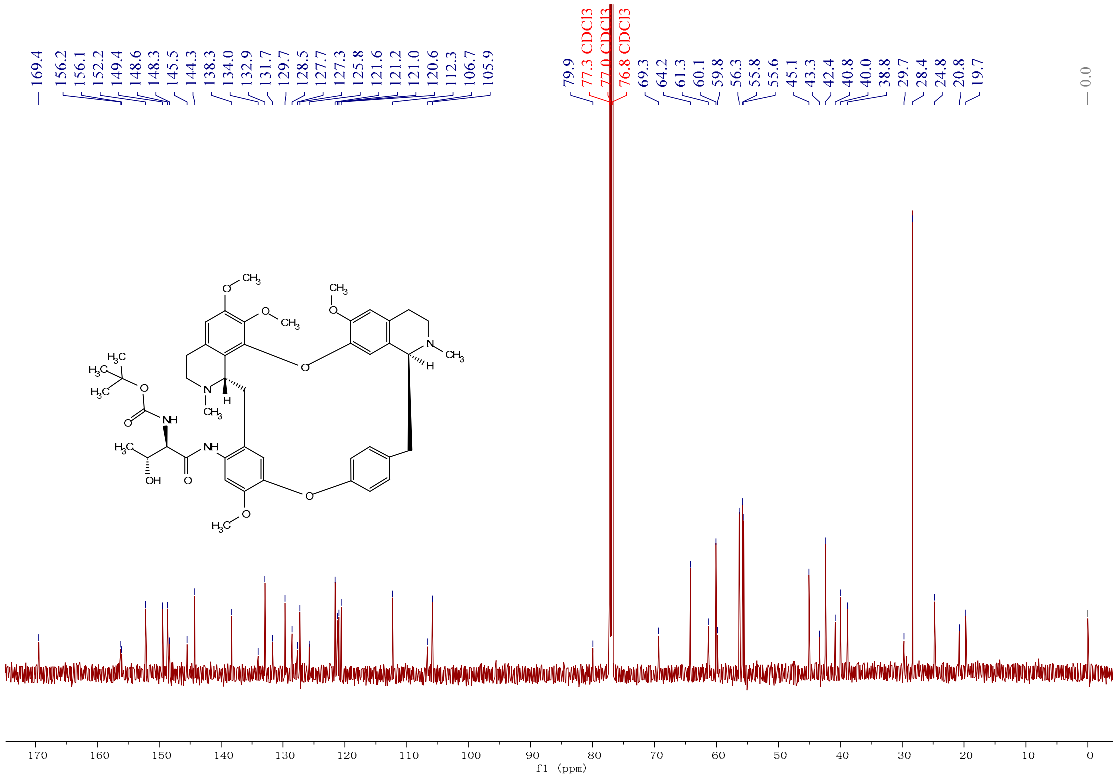

Figure S34. ^{13}C-NMR Spectra of **1f** in $CDCl_3$.

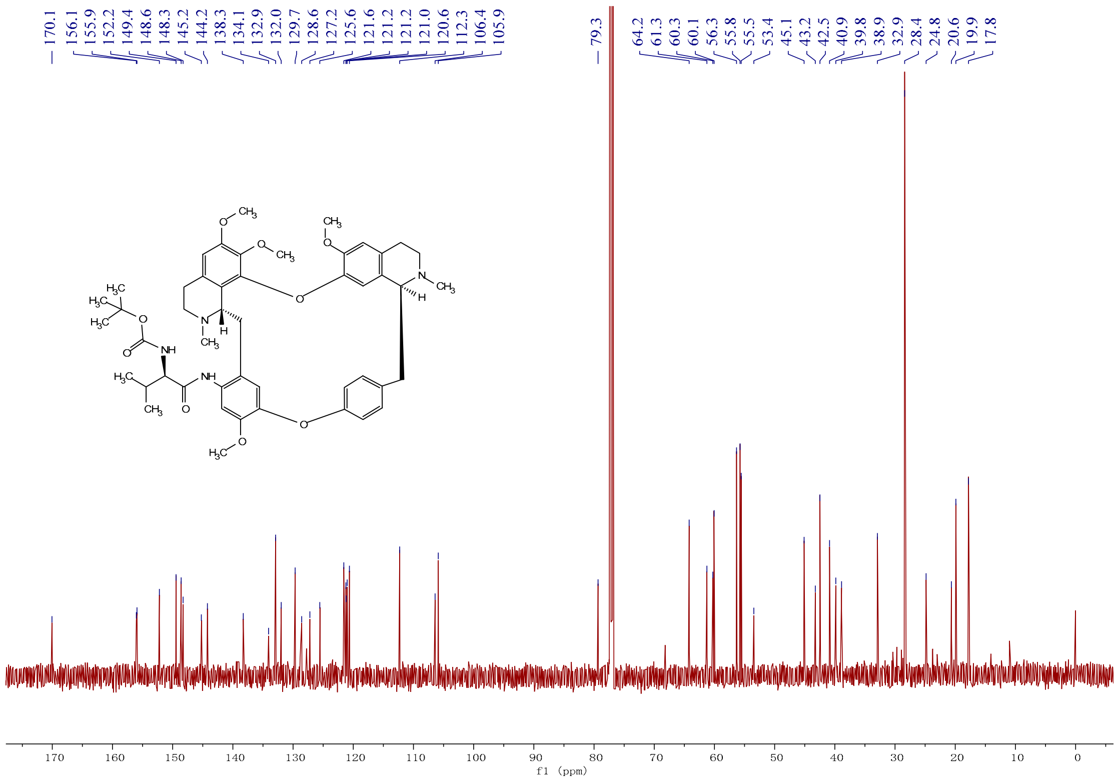

Figure S35. ^{13}C-NMR Spectra of **1g** in $CDCl_3$.

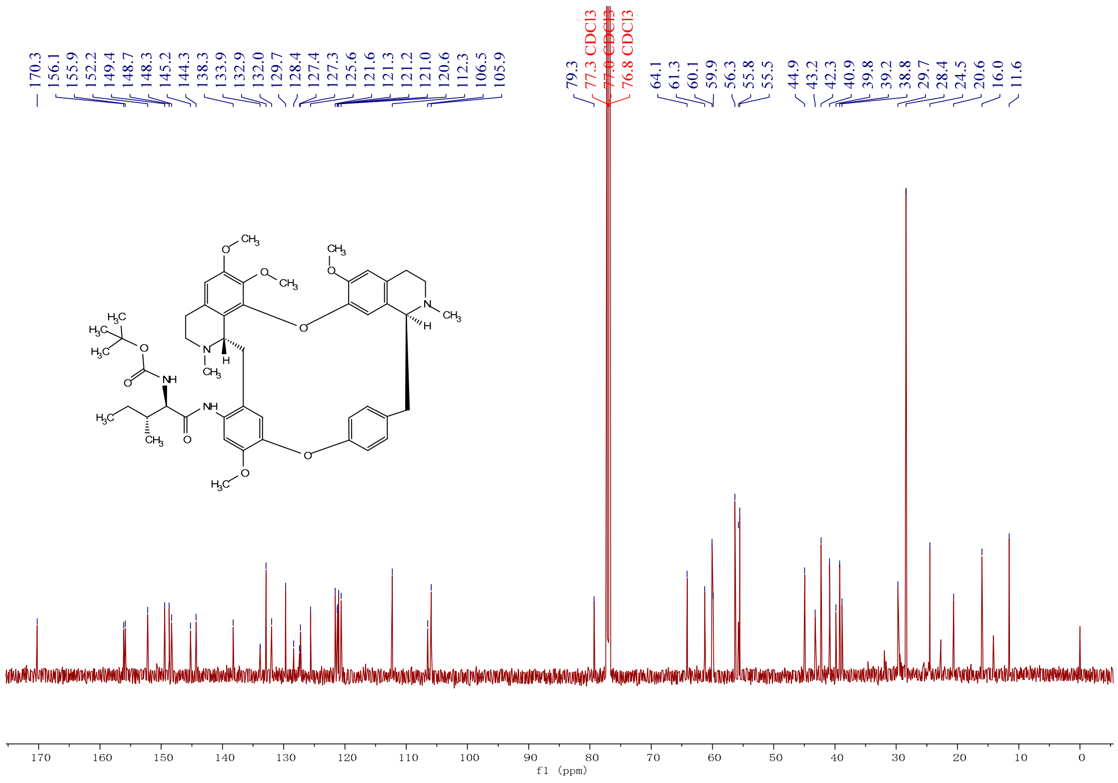

Figure S36. ^{13}C-NMR Spectra of **1h** in $CDCl_3$.

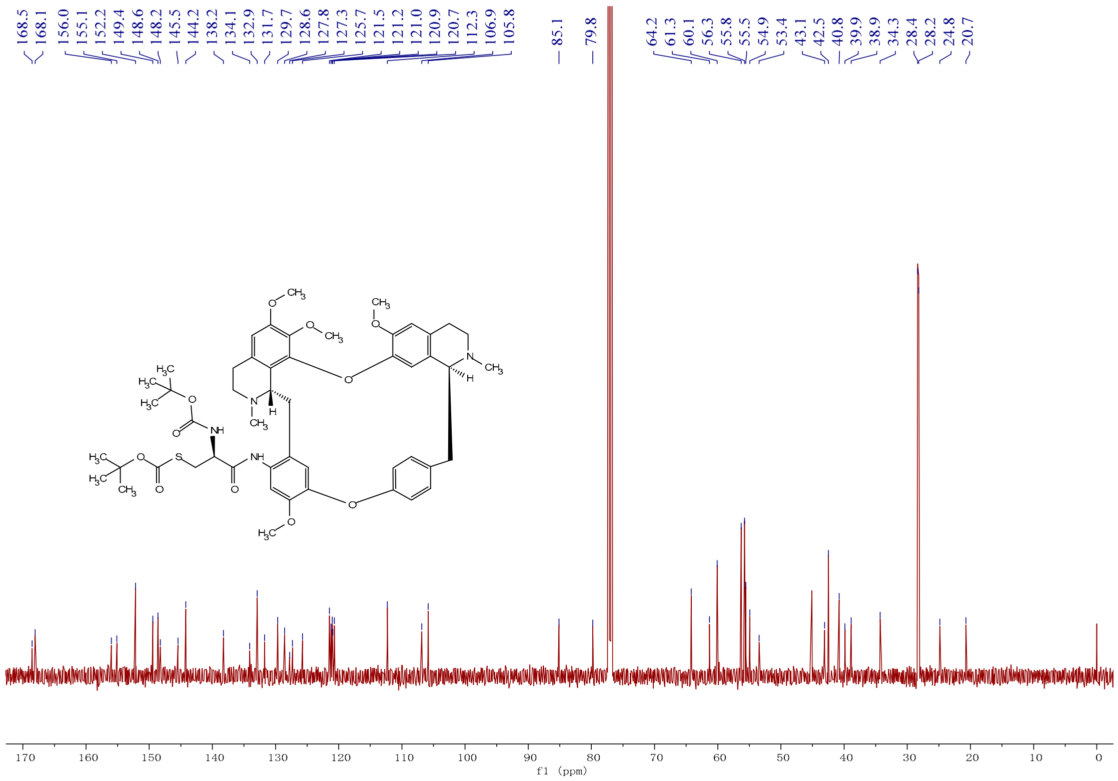

Figure S37. ^{13}C-NMR Spectra of **1i** in $CDCl_3$.

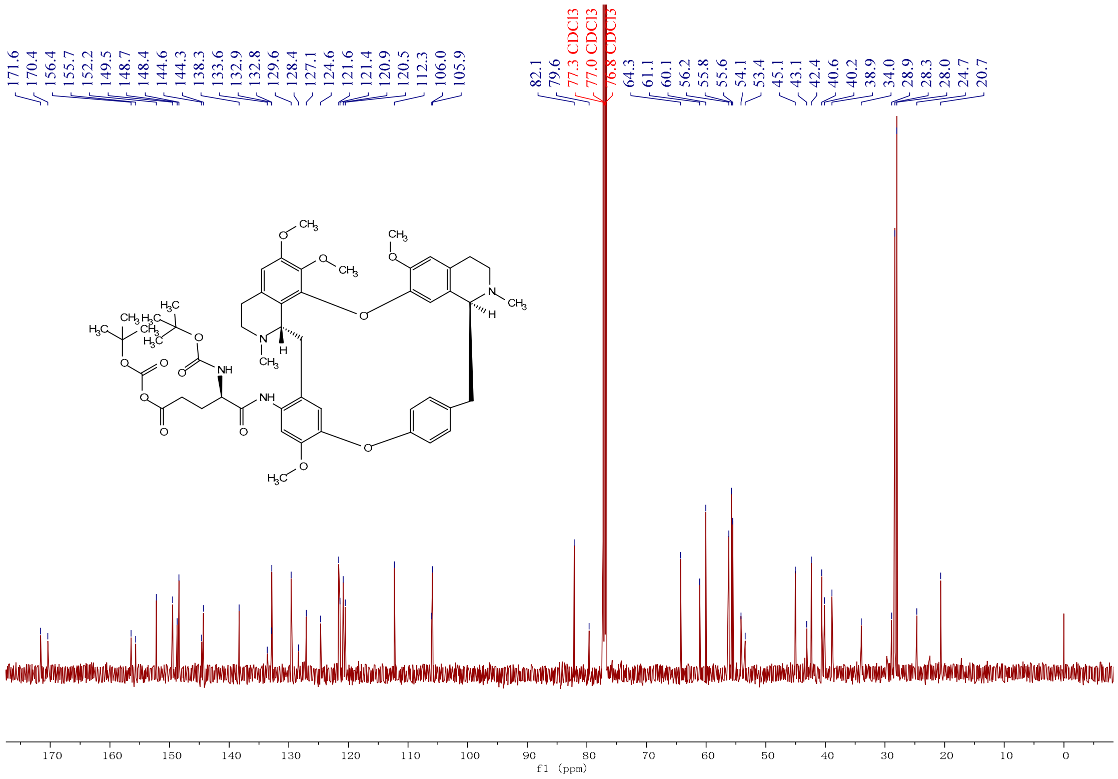

Figure S38. ^{13}C-NMR Spectra of **1j** in $CDCl_3$.

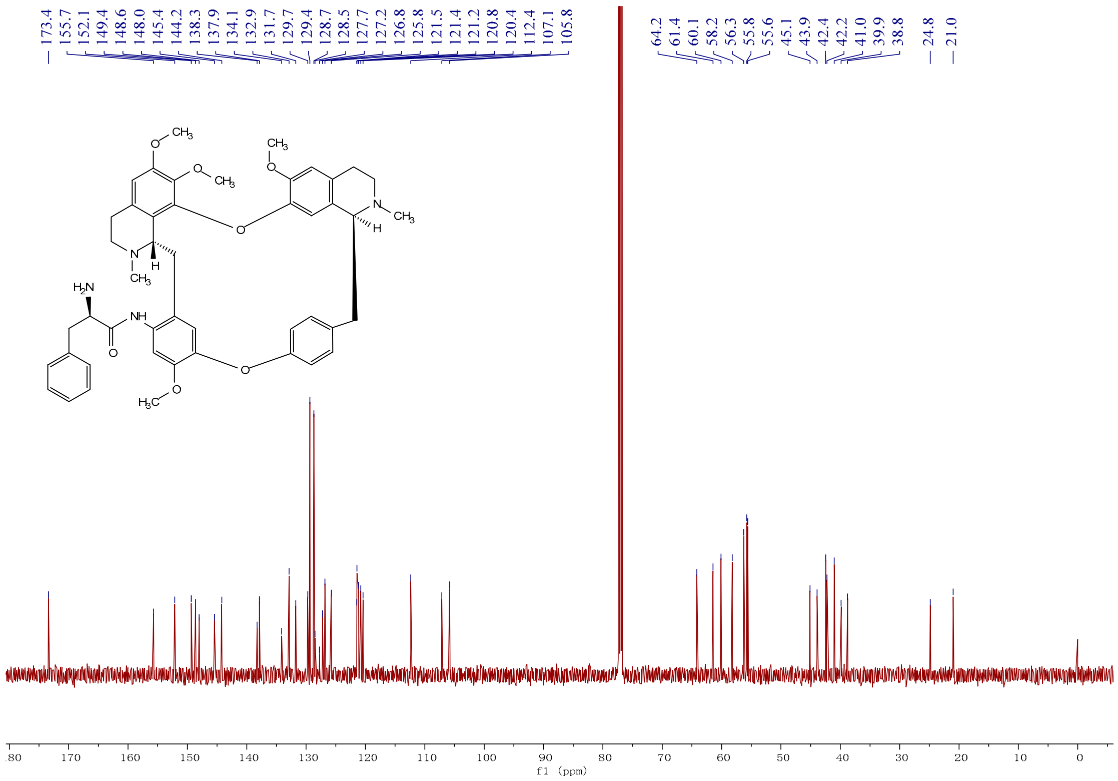

Figure S39. ^{13}C-NMR Spectra of **1k** in $CDCl_3$.

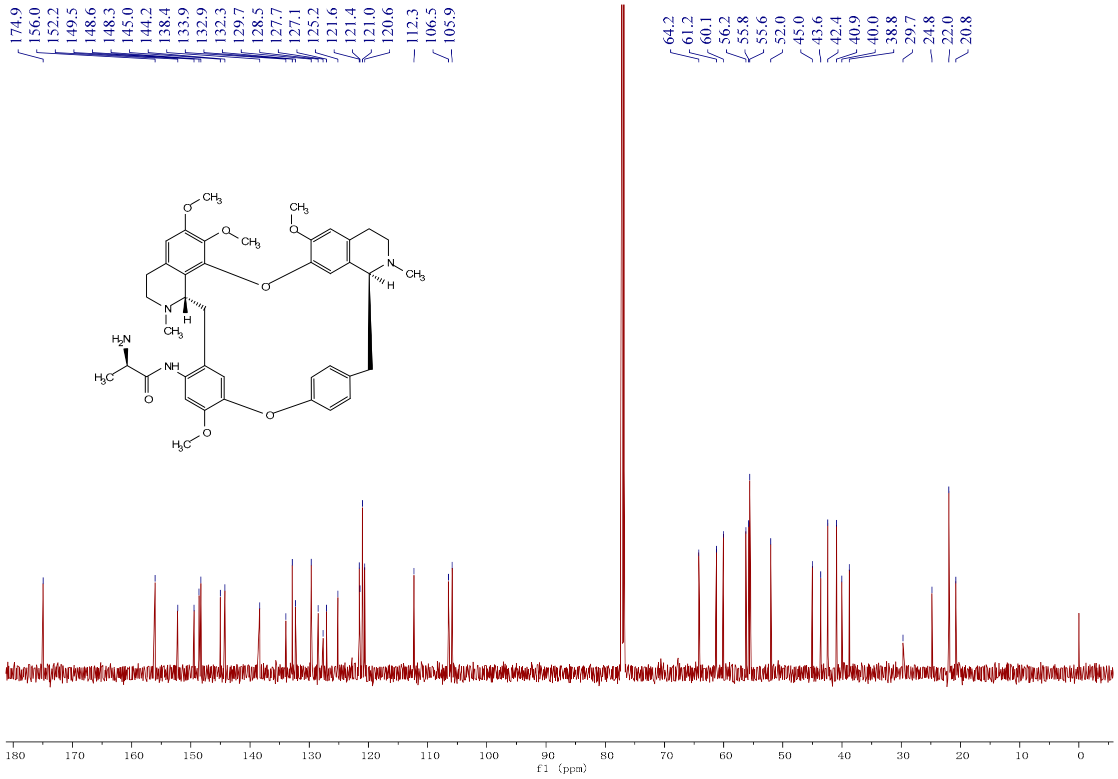

Figure S40. ^{13}C-NMR Spectra of **1l** in $CDCl_3$.

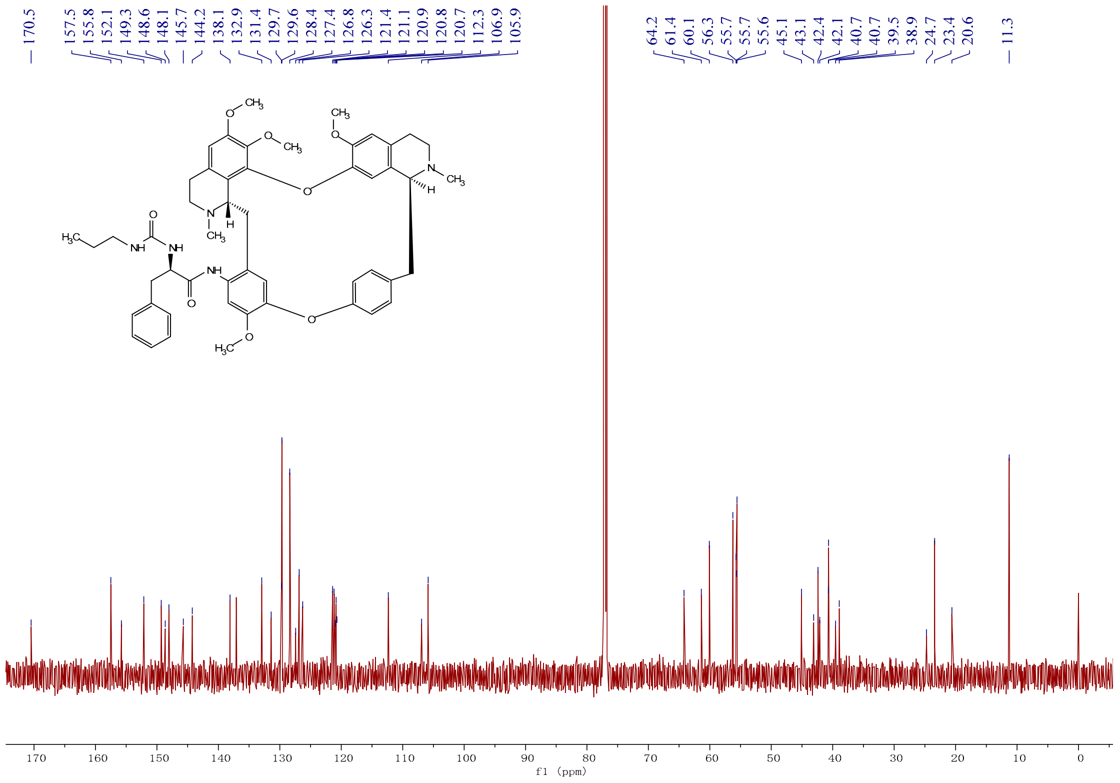

Figure S41. ^{13}C-NMR Spectra of **2a** in $CDCl_3$.

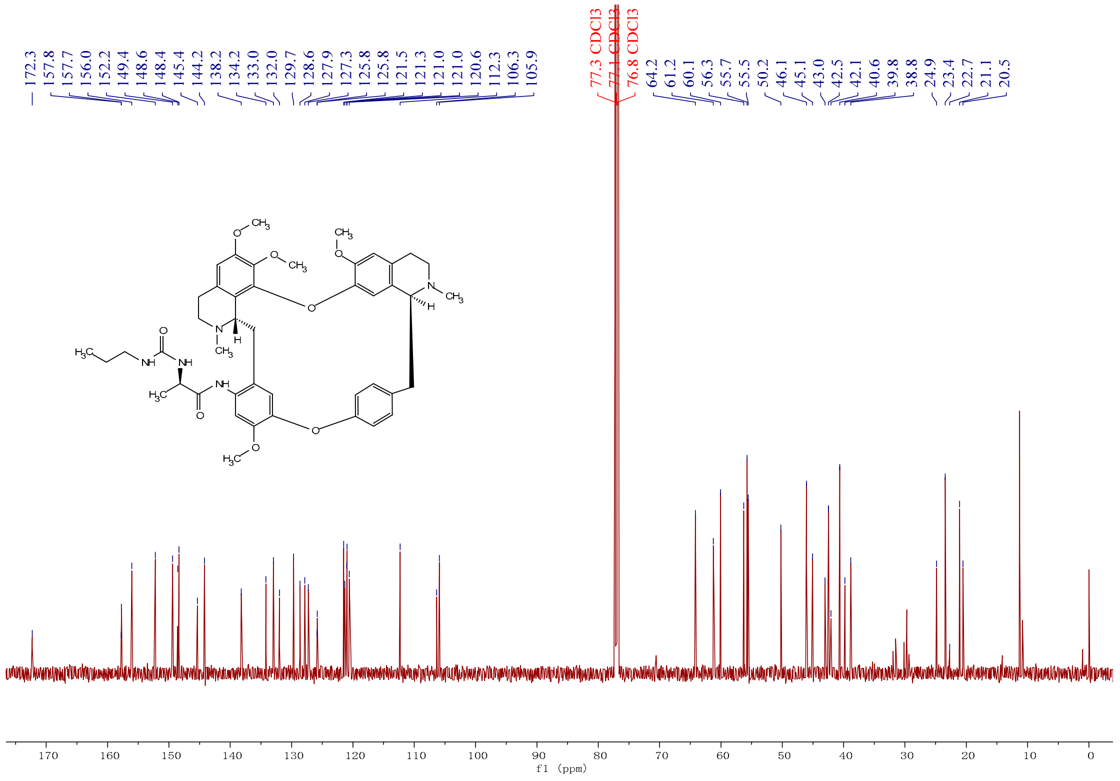

Figure S42. ^{13}C-NMR Spectra of **3a** in $CDCl_3$.

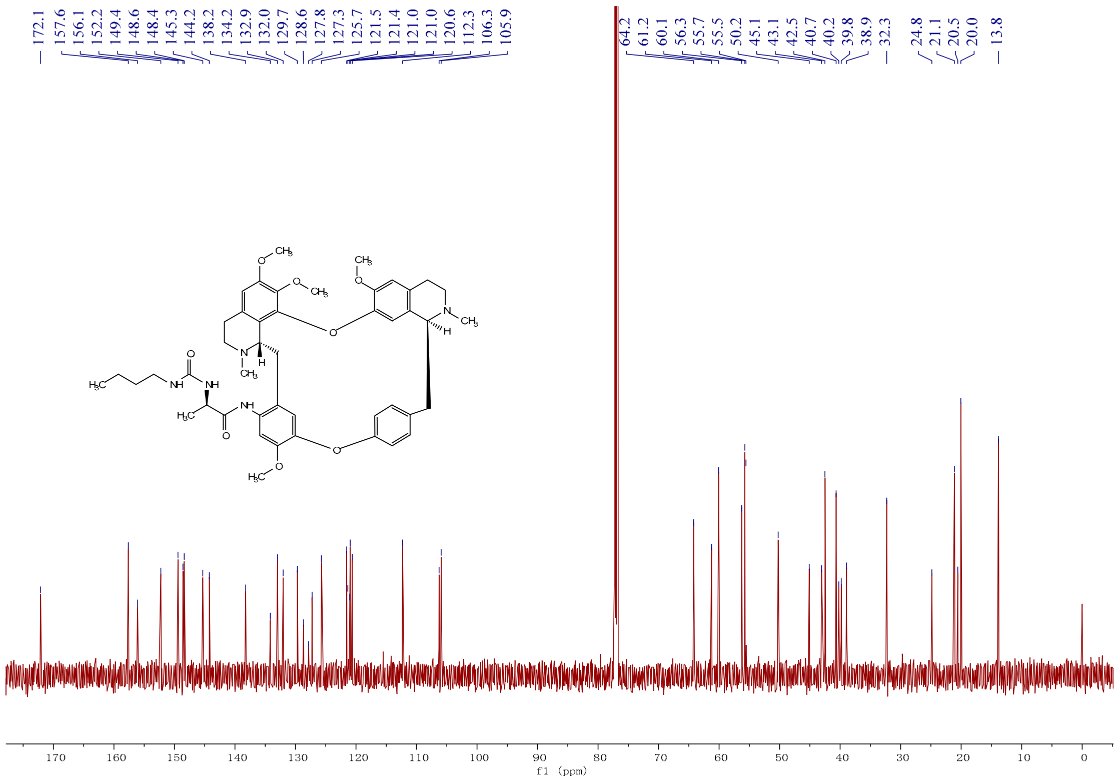

Figure S43. ^{13}C-NMR Spectra of **3b** in $CDCl_3$.

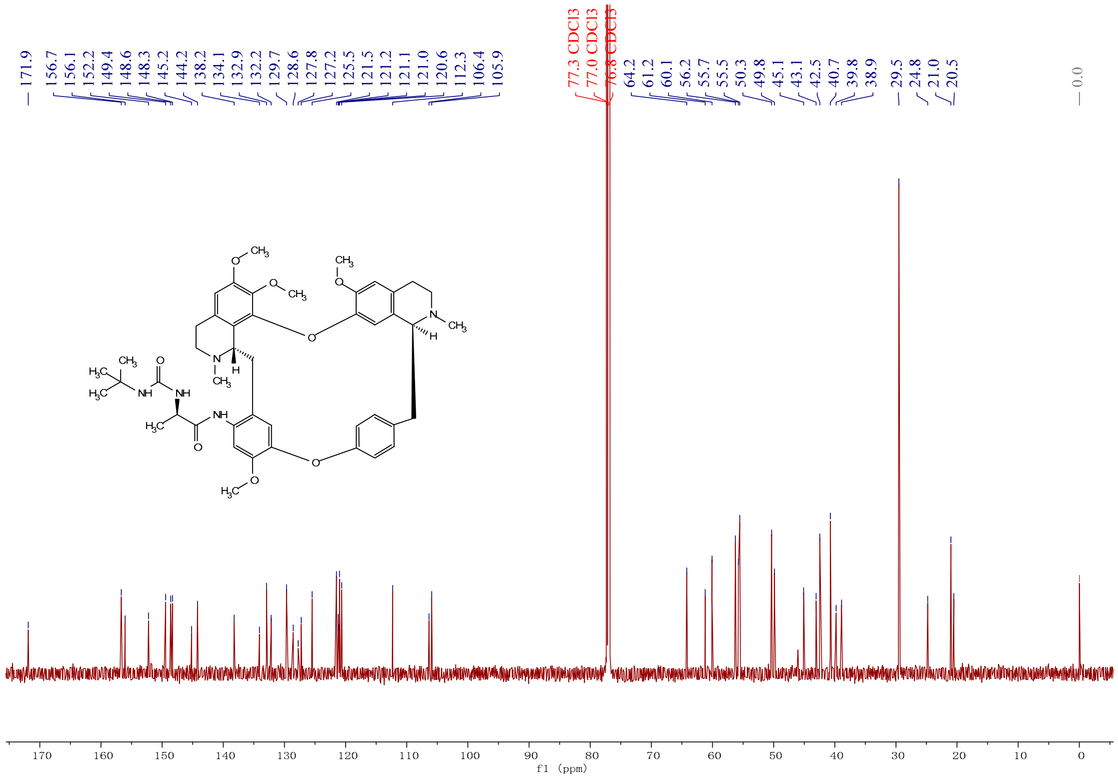

Figure S44. ^{13}C-NMR Spectra of **3c** in $CDCl_3$.

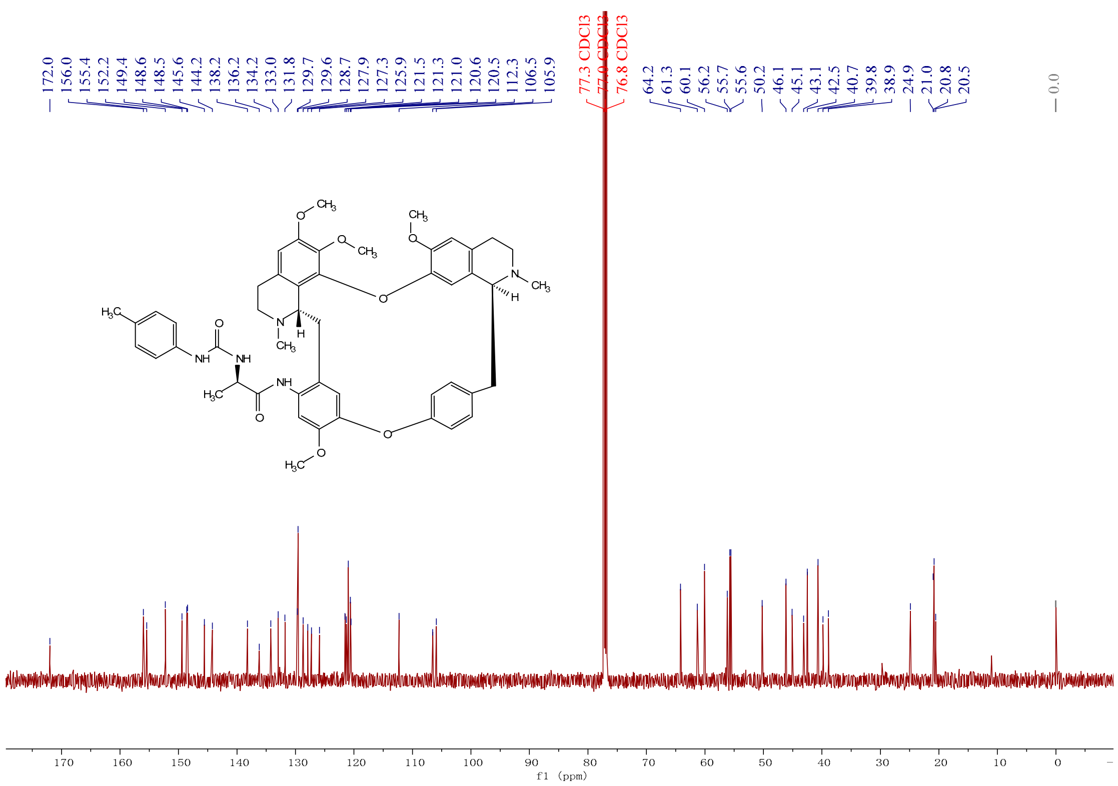

Figure S45. ^{13}C-NMR Spectra of **3d** in $CDCl_3$.

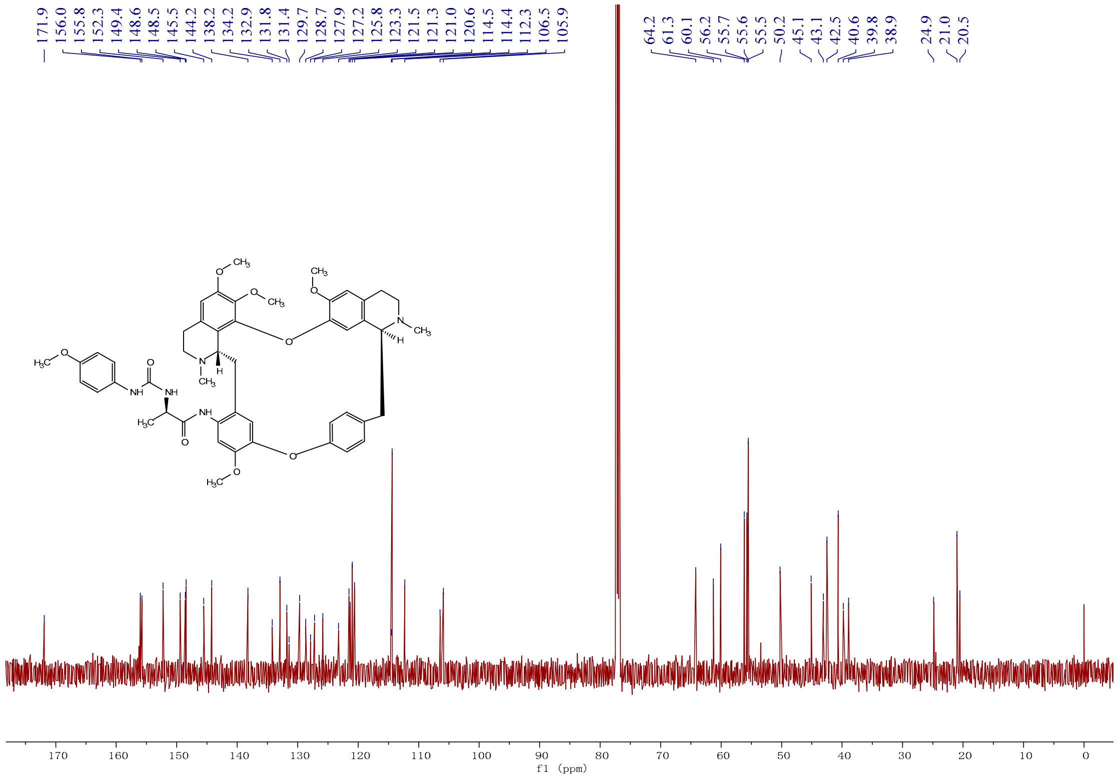

Figure S46. ^{13}C-NMR Spectra of **3e** in $CDCl_3$.

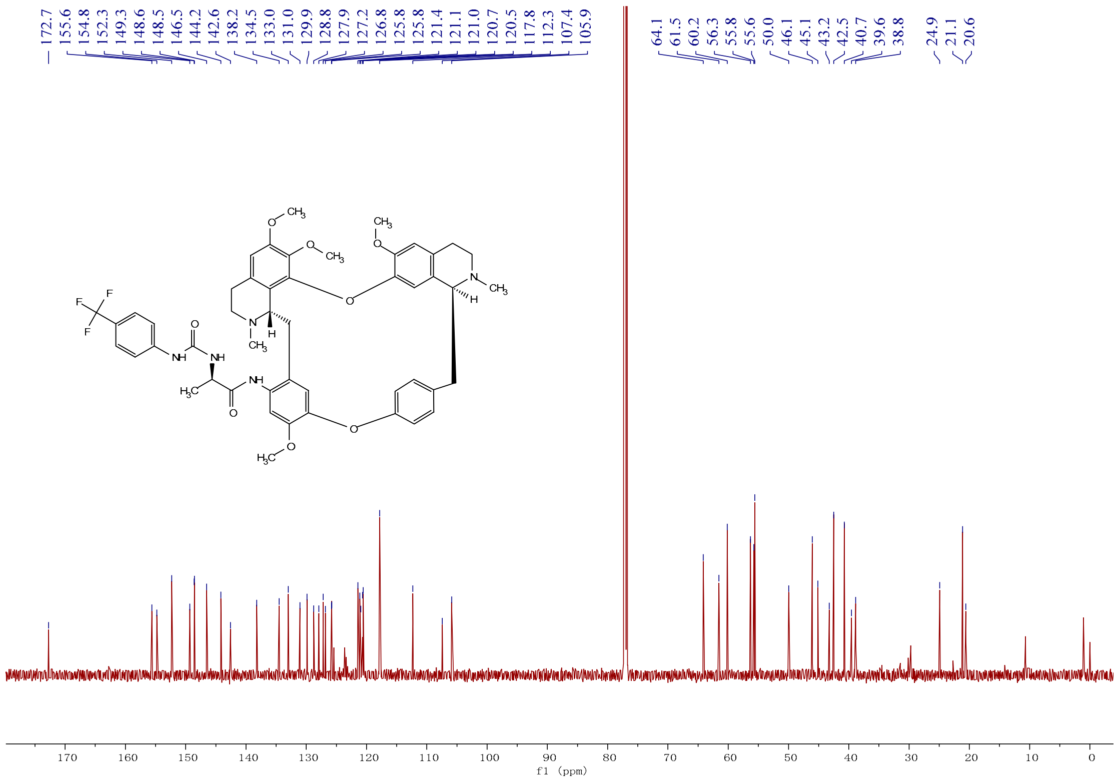

Figure S47. ^{13}C-NMR Spectra of **3f** in $CDCl_3$.

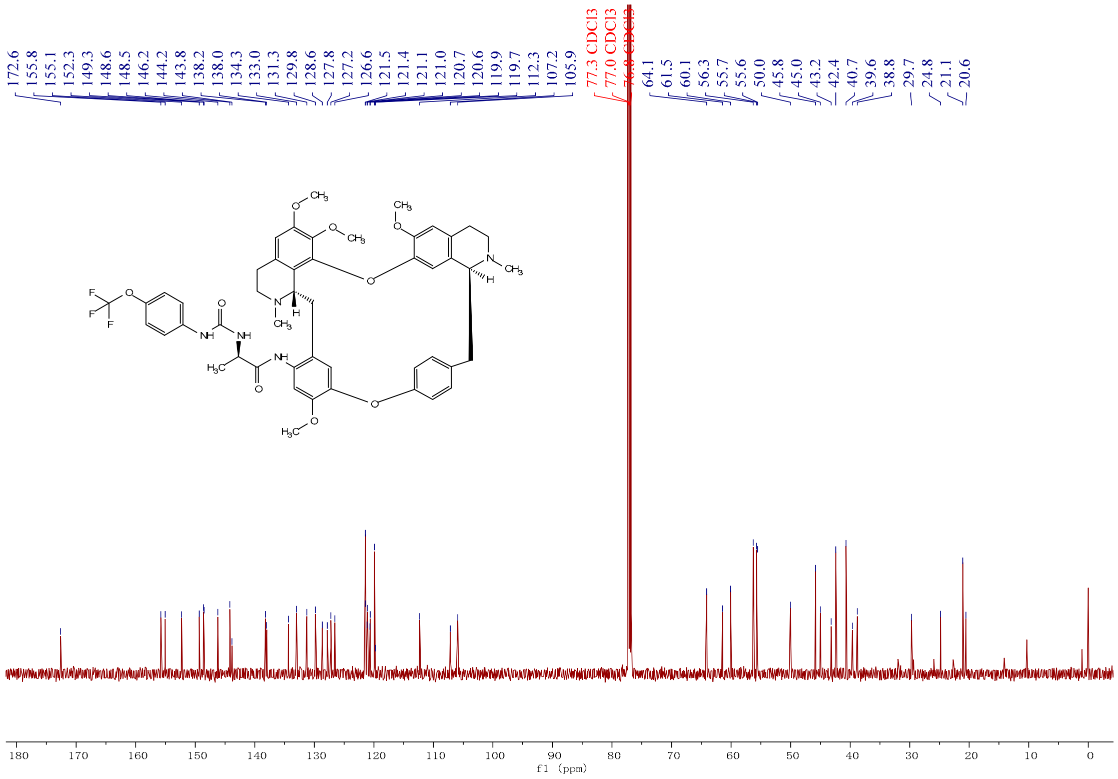

Figure S48. ^{13}C-NMR Spectra of **3g** in $CDCl_3$.

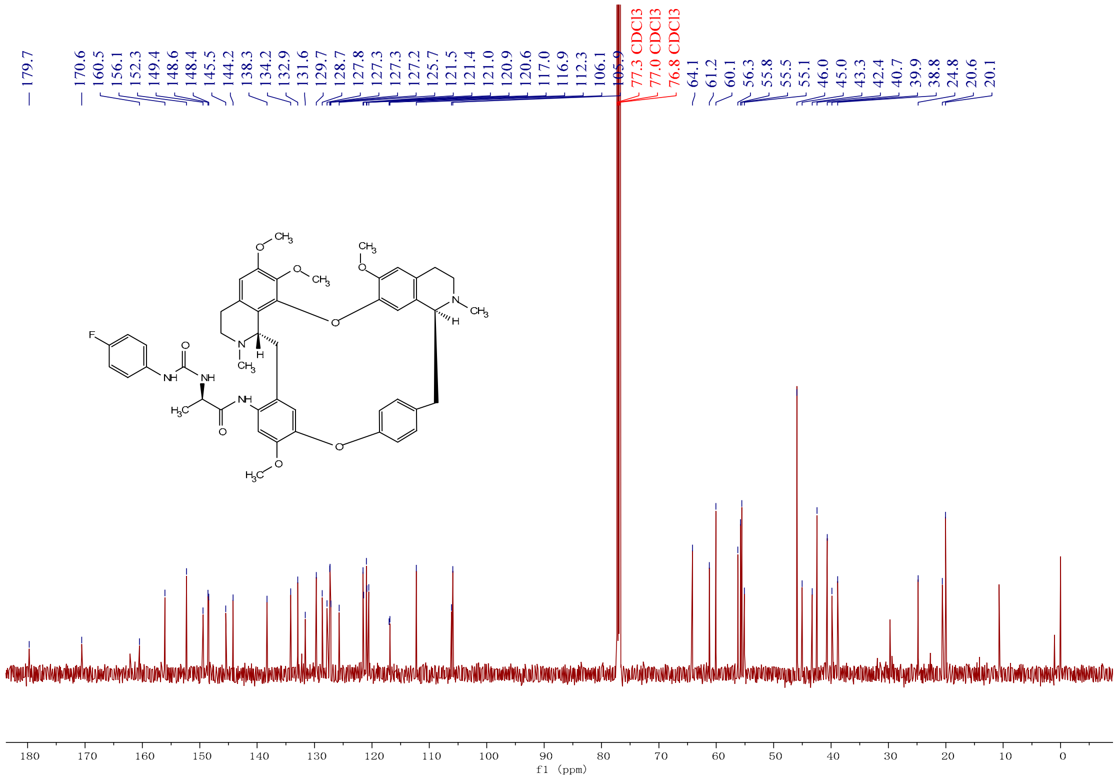

Figure S49. ^{13}C-NMR Spectra of **3h** in $CDCl_3$.

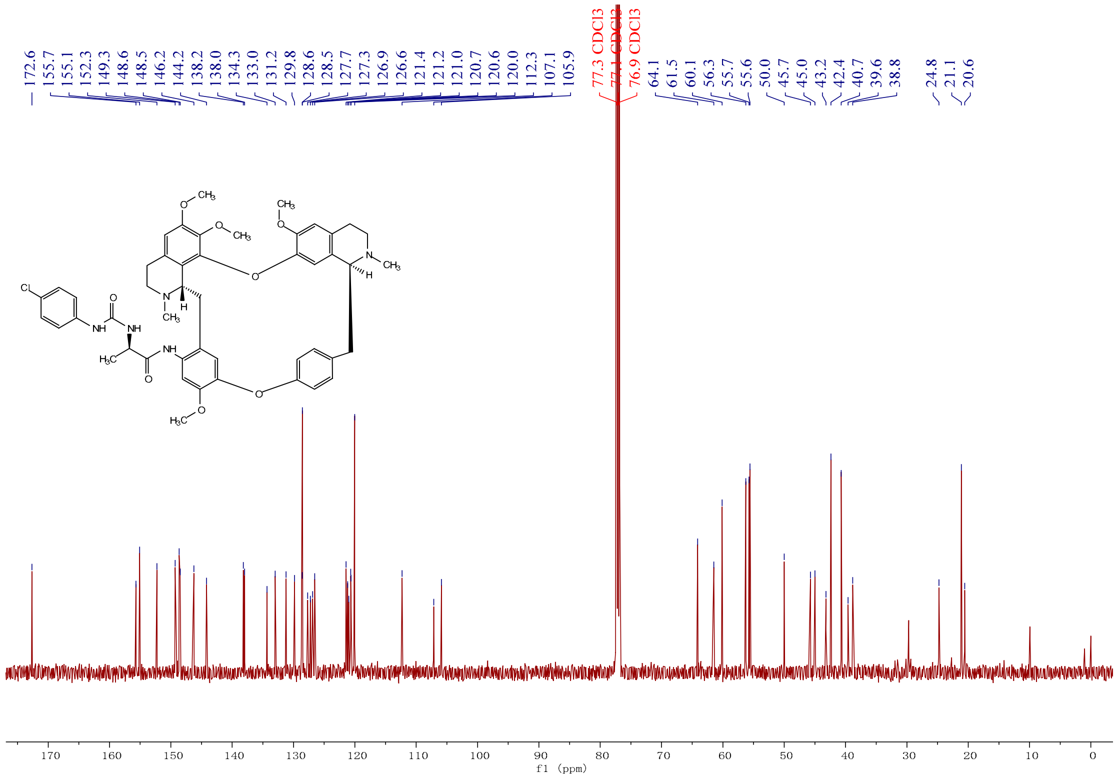

Figure S50. ^{13}C-NMR Spectra of **3i** in $CDCl_3$.

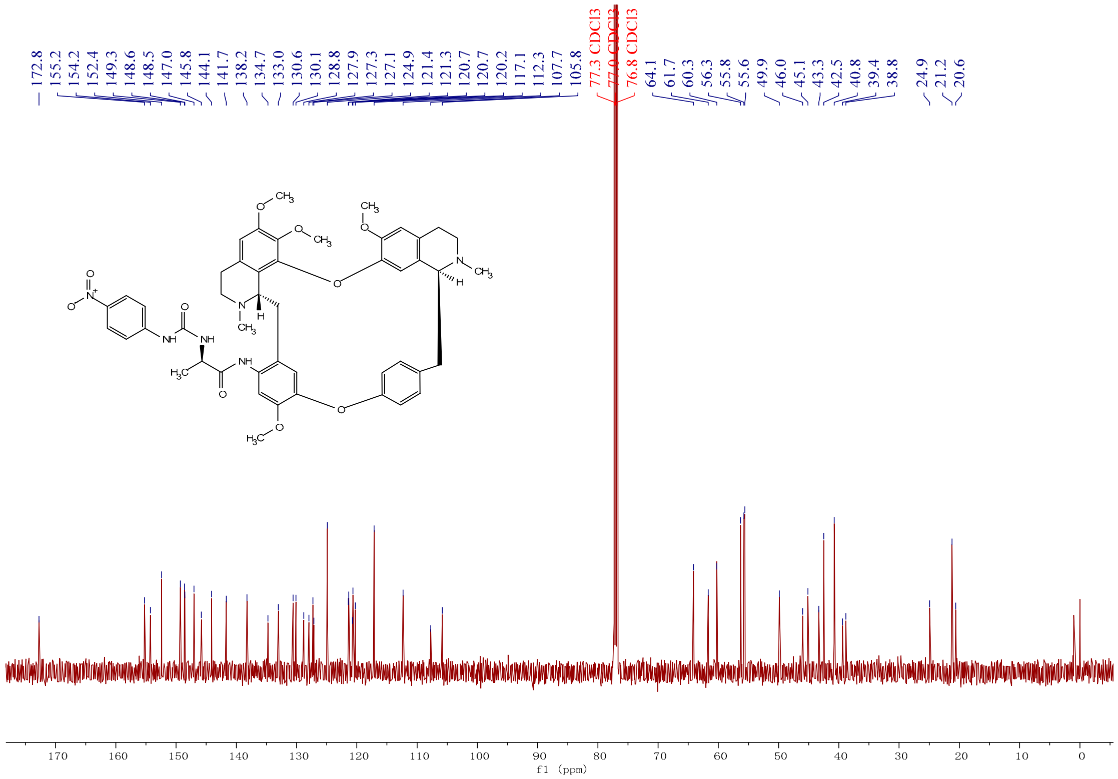

Figure S51. ^{13}C-NMR Spectra of **3j** in $CDCl_3$.

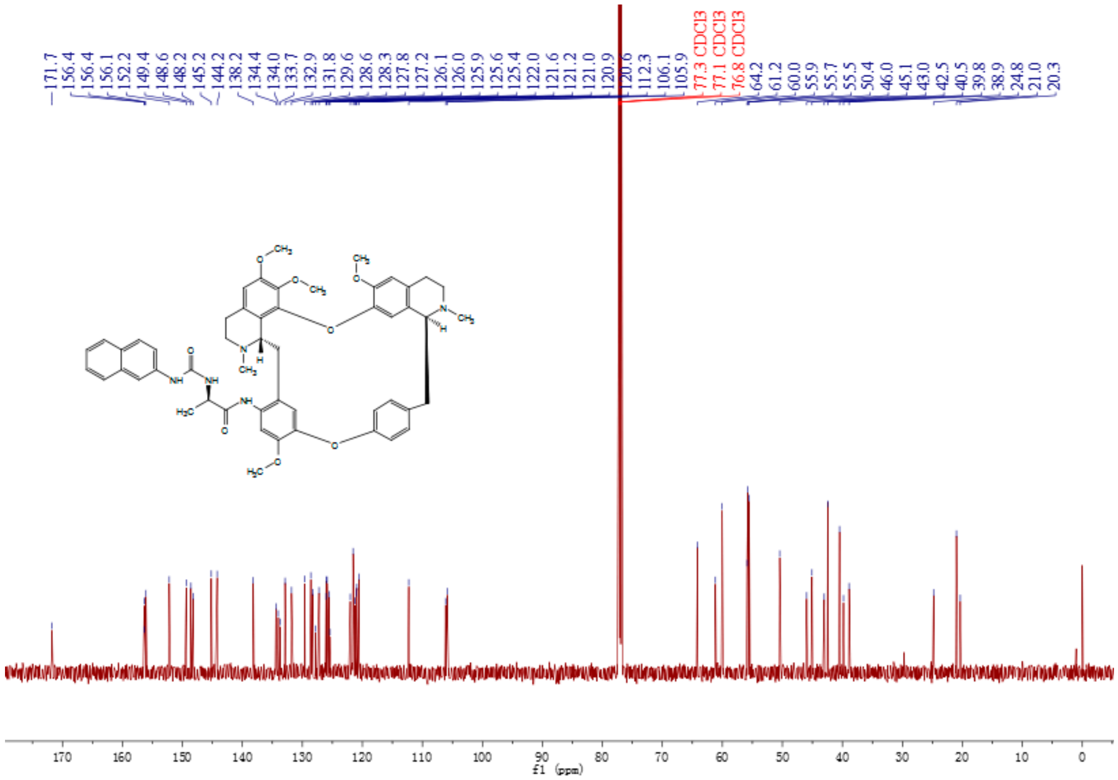

Figure S52. ^{13}C-NMR Spectra of **3k** in $CDCl_3$.
